# miR-494-3p overexpression promotes megakaryocytopoiesis in primary myelofibrosis hematopoietic stem/progenitor cells by targeting SOCS6

**DOI:** 10.18632/oncotarget.15226

**Published:** 2017-02-09

**Authors:** Sebastiano Rontauroli, Ruggiero Norfo, Valentina Pennucci, Roberta Zini, Samantha Ruberti, Elisa Bianchi, Simona Salati, Zelia Prudente, Chiara Rossi, Vittorio Rosti, Paola Guglielmelli, Giovanni Barosi, Alessandro Vannucchi, Enrico Tagliafico, Rossella Manfredini

**Affiliations:** ^1^ Centre for Regenerative Medicine, Life Sciences Department, University of Modena and Reggio Emilia, Modena, Italy; ^2^ Center for The Study of Myelofibrosis, Biotechnology Research Area, IRCCS Policlinico S. Matteo Foundation, Pavia, Italy; ^3^ CRIMM-Center for Research and Innovation for Myeloproliferative Neoplasms, AOU Careggi, and Department of Experimental and Clinical Medicine, University of Florence, Florence, Italy; ^4^ Center for Genome Research, University of Modena and Reggio Emilia, Modena, Italy

**Keywords:** miR-494-3p, SOCS6, primary myelofibrosis, megakaryocytopoiesis, CD34+ cells

## Abstract

Primary myelofibrosis (PMF) is a chronic Philadelphia-negative myeloproliferative neoplasm characterized by hematopoietic stem cell-derived clonal myeloproliferation, involving especially the megakaryocyte lineage. To better characterize how the altered expression of microRNAs might contribute to PMF pathogenesis, we have previously performed the integrative analysis of gene and microRNA expression profiles of PMF hematopoietic stem/progenitor cells (HSPCs), which allowed us to identify miR-494-3p as the upregulated microRNA predicted to target the highest number of downregulated mRNAs.

To elucidate the role of miR-494-3p in hematopoietic differentiation, in the present study we demonstrated that miR-494-3p enforced expression in normal HSPCs promotes megakaryocytopoiesis. Gene expression profiling upon miR-494-3p overexpression allowed the identification of genes commonly downregulated both after microRNA overexpression and in PMF CD34+ cells. Among them, suppressor of cytokine signaling 6 (SOCS6) was confirmed to be a miR-494-3p target by luciferase assay. Western blot analysis showed reduced level of SOCS6 protein as well as STAT3 activation in miR-494-3p overexpressing cells. Furthermore, transient inhibition of SOCS6 expression in HSPCs demonstrated that SOCS6 silencing stimulates megakaryocytopoiesis, mimicking the phenotypic effects observed upon miR-494-3p overexpression. Finally, to disclose the contribution of miR-494-3p upregulation to PMF pathogenesis, we performed inhibition experiments in PMF HSPCs, which showed that miR-494-3p silencing led to SOCS6 upregulation and impaired megakaryocyte differentiation.

Taken together, our results describe for the first time the role of miR-494-3p during normal HSPC differentiation and suggest that its increased expression, and the subsequent downregulation of its target SOCS6, might contribute to the megakaryocyte hyperplasia commonly observed in PMF patients.

## INTRODUCTION

Philadelphia-negative chronic myeloproliferative neoplasms (MPNs) are a heterogeneous spectrum of clonal hematological malignancies that include polycythemia vera (PV), essential thrombocythemia (ET) and myelofibrosis, which can be primary (PMF) or secondary to PV or ET [1]. MPNs arise from the mutation of a single hematopoietic stem cell (HSC), which following clonal expansion leads to the overt clinical phenotype [2, 3]. While PV is mainly characterized by an overproduction of erythrocytes, ET and PMF patients show increased numbers of dysplastic megakaryocytes in the bone marrow (BM) [4, 5]. In addition, in PMF patients the BM hematopoietic tissue is progressively substituted by extracellular matrix and fibroblasts over the course of the disease, eventually resulting in BM fibrosis and failure [5]. In particular, megakaryocytes (MKs) have been shown to be mainly responsible for the overproduction of various cytokines that drive the formation of the fibrous scar [6].

Despite the clinical heterogeneity of MPN patients, PV, ET and PMF share common genomic lesions. The JAK2V617F somatic mutation occurs in PMF and ET and almost all of PV patients, while mutations in the thrombopoietin receptor gene, MPL, and the calreticulin gene, CALR, are frequent in ET and PMF patients [7–10]. Nevertheless, triple-negative patients, who do not carry any of the three “driver” mutations, represent almost 10% of ET and PMF cases. In addition, several other identified lesions (i.e. loss-of-function mutations in EZH2, ASXL1, TET2, JARID2) are less frequent and usually co-occur alongside the afore-mentioned hits [11].

Although analysis of the pathogenetic role of such mutations has been a topic of extensive research, none of the genomic lesions described is able alone to entirely explain all the pathological features observed in PMF. In fact, fewer studies address the importance of aberrantly expressed coding and non-coding RNAs that may affect the behavior of mutant HSCs. Of note, several gene expression profiling studies highlighted the central role of the activation of the JAK/STAT pathway in the disease pathogenesis, in keeping with previous observations reporting the activation of this signaling pathway induced by the three “driver” mutations (i.e. JAK2, MPL and CALR mutations) [12–14]. Indeed, Rampal and colleagues demonstrated that MPN patients display a transcriptional signature consistent with the activation of JAK2 signaling regardless of their mutational status [15]. Furthermore, non-coding RNA altered expression has already been reported in MPN patients in both granulocytes and hematopoietic stem and progenitor CD34+ cells (HSPCs) [16–20]. Recently, our group has shown that HSPCs from PMF patients aberrantly express several microRNAs (miRNAs), which cooperate to fine tune the expression of a number of target mRNAs. In particular, integrative analysis of dysregulated miRNAs and mRNAs allowed the identification of several aberrantly regulated miRNA-mRNA target pairs organized in complex interaction networks where either different miRNAs contribute to regulate the expression of a particular gene, or a single miRNA can target a high number of mRNAs [21].

Here we focus our attention on miR-494-3p, which is upregulated in PMF HSPCs and is predicted to target the highest number of aberrantly expressed targets, among all the upregulated miRNAs. We describe for the first time the biological function of miR-494-3p in normal HSPC differentiation, demonstrating that its enforced expression leads to increased production of megakaryocytic cells and providing evidence that its phenotypic effects are mediated by SOCS6 downregulation. Finally, we show that miR-494-3p silencing in PMF HSPCs affects MK production.

Collectively, our data highlight miR-494-3p as a key player in normal HSPC differentiation and suggest that the deregulation of miR-494-3p/SOCS6 axis might act as a new pathogenetic mechanism contributing to the overproduction of MKs in the bone marrow of PMF patients.

## RESULTS

### miR-494-3p is upregulated in PMF HSPCs

**Figure 1:**
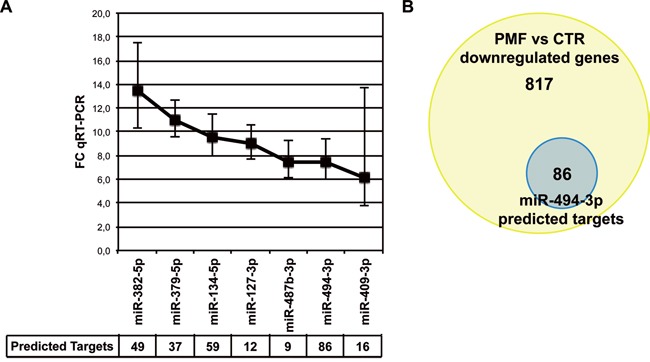
miR-494-3p is overexpressed in PMF CD34+ cells **A.** Line graph showing the most upregulated miRNAs in PMF CD34+ cells defined as showing more than 2-fold increase in the microarrays (false discovery rate<0.05) and more than 5-fold increase in quantitative reverse transcription polimerase chain reaction (qRT-PCR) (p-value<0.05). qRT-PCR analysis was performed in CD34+ cells on a cohort of 10 PMF patients and 8 healthy donors, as reported in our previous work (p<0.05) [21]. The table at the bottom of the graph represents the number of predicted downregulated targets for each miRNA in PMF CD34+ cells according to integrative analysis of gene and miRNA expression profiles. **B.** Venn diagram displaying the number of PMF downregulated genes targeted by at least one overexpressed miRNA and the number of miR-494-3p predicted targets. Abbreviations: FC, fold change.

As recently reported by our group, we performed miRNA and gene expression profiling of CD34+ HSPCs isolated from PMF patients and healthy controls [21]. Integrative analysis of these profiles by means of Ingenuity Pathway Analysis unveiled a network involving the most upregulated miRNAs in PMF CD34+ cells (Figure 1A) and their common downregulated predicted targets (Supplementary Figure 1).

Interestingly, our study revealed the up-regulation of a previously poorly characterized miRNA, miR-494-3p, in CD34+ cells from PMF patients. Of note, among the most upregulated miRNAs, miR-494-3p was associated to the highest number of downregulated target mRNAs (Figure 1A, Supplementary Table 1). In detail, out of 903 downregulated genes associated with at least 1 overexpressed miRNA, 86 were predicted targets of miR-494-3p, amounting to 9.5% of all miRNA-downregulated genes (Figure 1B; Supplementary Table 2).

### miR-494-3p overexpression affects HSPC differentiation

In order to understand the biological role of miR-494-3p during hematopoietic commitment and differentiation, we overexpressed it in cord blood-derived (CB) HSPCs and assessed its biological effects by means of several *in vitro* differentiation assays.

**Figure 2:**
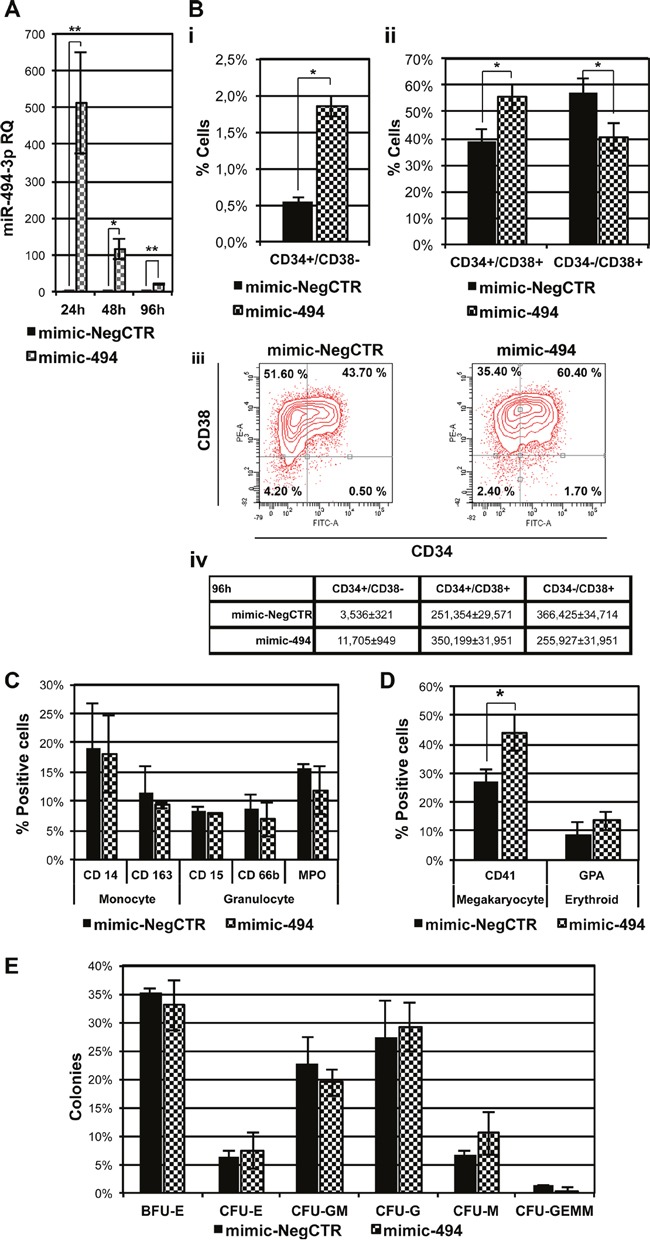
Effect of miR-494-3p on HSPCs differentiation **A.** Expression levels of miR-494-3p in CB CD34+ cells were evaluated 24, 48 and 96 hours after the last nucleofection by means of qRT-PCR. Data are reported as RQ mean ± S.E.M of 5 independent experiments. Results were normalized to mimic-NegCTR sample and U6 was selected as endogenous control. **B.** Subpanels *i* and *ii* represent statistical analysis of flow cytometry evaluation of CD34 and CD38 protein expression in CB CD34+ cells cultured in multilineage conditions in the presence of HS at 96 hours upon mimic nucleofection (n=2). Subpanel *iii* shows the flow cytometry analysis of a representative experiment. Subpanel *iv* represents the absolute numbers of cells belonging to the three different populations: CD34+/CD38-, CD34+/CD38+ and CD34-/CD38+. Absolute cell numbers were calculated, according to the percentage of cells for each population, starting from the average total cell number in each sample. **C-D.** Flow cytometry analysis of expression of monocytic (CD14, CD163), granulocytic (CD15, CD66b, MPO), megakaryocytic (CD41) and erythroid (GPA) differentiation markers in CB CD34+ cells overexpressing miR-494-3p maintained in multilineage conditions in the presence of HS (C) or the serum substitute BIT 9500 (D) at day 11 of cell culture. (n=3) **E.** Results of the statistical analysis of methylcellulose clonogenic assay of CB CD34+ cells overexpressing miR-494-3p. Cells were plated 24 hours after mimic nucleofection and colonies were scored at day 14 (n=3). Results are reported as mean ± S.E.M. *, p≤0.05 Abbreviations: CFU, colony-forming unit; BFU, burst-forming unit; E, erythroid; GM, granulo-monocyte; G, granulocyte; M, monocyte; GEMM, granulocyte, erythrocyte, macrophage, megakaryocyte.

A significant miR-494-3p overexpression was detected by quantitative reverse transcription polymerase chain reaction (qRT-PCR) after 24, 48 and 96 hours upon transfection of CD34+ cells with miR-494-3p miRNA mimic (mimic-494), as depicted in Figure 2A.

Interestingly, flow cytometry evaluation of the co-expression of the CD34 and CD38 antigens in cells cultured in multilineage conditions revealed that the more immature CD34+/CD38- cell fraction was significantly expanded in mimic-494 sample compared to the control at 96 hours after transfection. Furthermore, we also observed the amplification of the CD34+/CD38+ population at the expense of the more mature CD34-/CD38+ cell fraction, as demonstrated by the increase in the percentage and absolute cell number of double positive fraction (Figure 2B).

Moreover, in order to study the influence of miR-494-3p overexpression on HSPC differentiation towards the myeloid lineage, we measured the expression of several markers in transfected cells cultured in the presence of human serum (HS), monitoring the expression levels of CD14 and CD163 for monocyte/macrophage differentiation and CD15, CD66b and MPO expression for granulocyte differentiation. As shown in Figure 2C, miR-494-3p overexpression does not have any influence on the cell fraction expressing either monocyte or granulocyte specific antigens. Since the presence of HS inhibits erythroid and MK differentiation of HSPCs *in vitro*, we also explored the effects of miR-494-3p overexpression on erythroid and MK differentiation, in multilineage culture conditions in the presence of a serum substitute, by assessing the expression of an erythroid marker (GPA) as well as a megakaryocytic marker (CD41). Flow cytometry analysis revealed that an increased fraction of CD41+ cells was associated with miR-494-3p overexpression, while no statistically significant differences in GPA expression were observed (Figure 2D). Accordingly, methylcellulose-based clonogenic assays did not show significant differences in myeloid or erythroid colonies (CFU-G, CFU-M, CFU-GM, BFU-E, CFU-E) upon miR-494-3p overexpression (Figure 2E). Therefore, these results suggest that miR-494-3p overexpression in CD34+ cells promotes megakaryocytopoiesis, while it does not affect differentiation towards myeloid and erythroid lineages in multilineage culture conditions.

**Figure 3:**
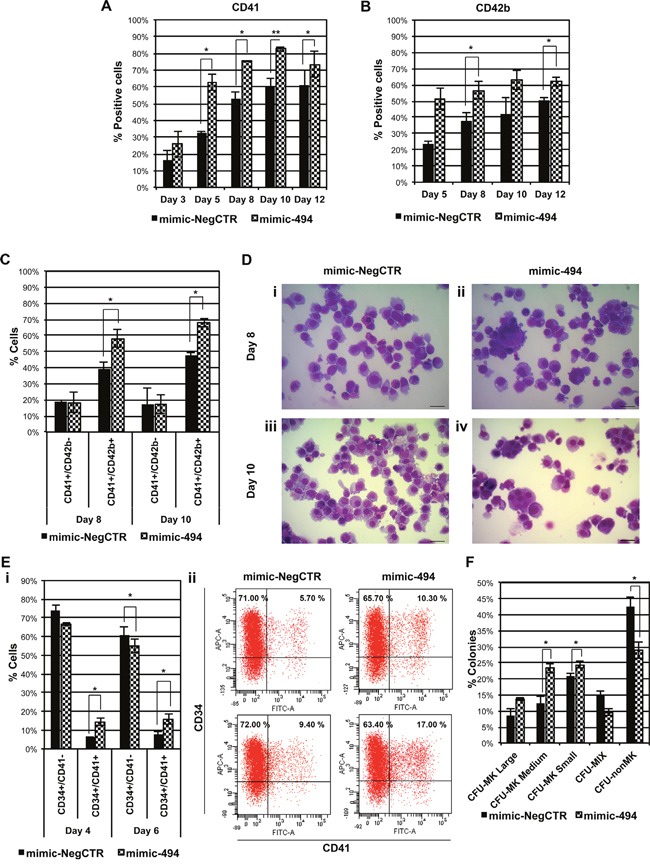
miR-494-3p overexpression affects MK differentiation of HSPCs **A-B.** Bar-graphs represent the statistical analysis of the flow cytometry evaluation of CD41 (A) and CD42b (B) cell markers in CB CD34+ cells overexpressing miR-494-3p. Marker expression was assessed at days 3, 5, 8, 10 and 12 of serum-free MK unilineage cell culture (n=3). **C.** Flow cytometry analysis of co-expression of CD41 and CD42b surface antigens at days 8 and 10 of MK unilineage culture after mimic nucleofection in CB CD34+ cells (n=3). **D.** Representative morfological analysis of mimic-NegCTR (i-iii) and mimic-494 (ii-iv) samples after May-Grünwald-Giemsa staining at days 8 and 10 of MK unilineage culture. Magnification, x400. Scale bar: 100 μm. **E.** Subpanel *i* represents the statistical analysis of flow cytometry analysis of co-expression of CD34 and CD41 surface antigens at days 4 and 6 post mimic electroporation in serum free multilineage culture (n=3). Subpanel *ii* shows corresponding dot plot graphs of a representative experiment. **F.** Results of the statistical analysis of collagen-based clonogenic assay of CB CD34+ cells overexpressing miR-494-3p. Cells were seeded in semisolid culture medium 24 hours after the last nucleofection and colonies were scored after 11 days (n=3). Results are reported as mean ± S.E.M. **, p≤0.01; *, p≤0.05 Abbreviations: CFU, colony-forming unit; MK, megakaryocyte; MIX, mixed; nonMK, other than megakaryocyte.

Then, in order to better characterize the role of miR-494-3p in MK differentiation, we performed overexpression experiments in CD34+ cells cultured in MK unilineage culture conditions. Again, the immunophenotype confirmed that the percentage of cells expressing CD41 or the late MK antigen CD42b were significantly higher in mimic-494 samples (Figure 3A & 3B). Furthermore, flow cytometry analysis indicated that the percentage of CD41+/CD42b+ expressing cells, which are late MKs, were significantly higher in miR-494-3p overexpressing cells while no changes in the percentage of the CD41+/CD42b- fraction were detected (Figure 3C). Accordingly, upon miR-494-3p overexpression, morphological analysis showed an expansion of the MK lineage, as demonstrated by the increase of precursors and hyperdiploid megakaryocytic cells (Figure 3D). Additionally, to check whether miR-494-3p could exert an effect also on early stages of MK commitment we evaluated the co-expression of the stem/progenitor marker CD34 and the MK marker CD41. Flow cytometry analysis indicated that miR-494-3p overexpression led to the expansion of the double-positive population at days 4 and 6 of liquid culture, while the percentage of CD34+/CD41- cells was decreased accordingly (Figure 3E). Collagen-based clonogenic assay confirmed these results showing a significant increase in the percentage of medium-sized MK colony forming units (CFU-MK Medium), which arise from more primitive MK progenitors. Of note, the percentage of small CFU-MK, which derive from more mature MK progenitors, were similarly increased after miR-494-3p overexpression (Figure 3F). As a whole, these results demonstrated that miR-494-3p overexpression promotes megakaryocytopoiesis acting both on early and late stages of the differentiation process in normal CD34+ cells.

### miR-494-3p-overexpression alters HSPC gene expression

**Figure 4:**
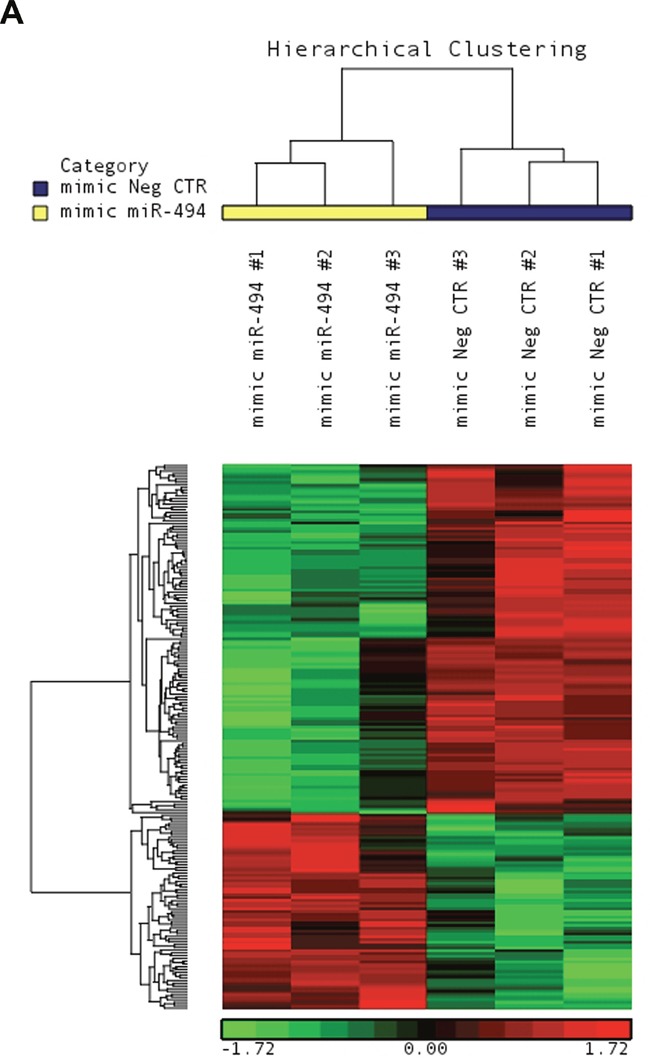
Microarray analysis upon miR-494-3p overexpression **A.** Hierarchical clustering of mimic-NegCTR (mimic Neg CTR) and mimic-494 (mimic miR-494) samples based on the list of 196 differentially expressed transcripts.

To better characterize the molecular mechanisms underlying the effects of miR-494-3p on HSPC differentiation, we carried out a microarray-based gene expression analysis at 24 hours after the last nucleofection to compare miR-494-3p overexpressing cells vs control cells. The list of 196 differentially expressed transcripts is showed in Figure 4 and Supplementary Table 3.

Among the downregulated genes, suppressor of cytokine signaling 6 (SOCS6) resulted to be the miR-494-3p predicted target associated to the most favorable context+ score according to TargetScanHuman database (Supplementary Table 4). Intriguingly, SOCS6 was also downregulated in PMF HSPCs, which showed miR-494-3p upregulation as well [21]. Moreover, SOCS6 is a key player in the negative regulation of the JAK/STAT signaling pathway [22], whose constitutive activation is one of the most important molecular mechanisms underlying MPN pathogenesis. These observations, along with our findings, prompted us to further investigate the interaction between miR-494-3p and SOCS6 and their possible role in HSPC differentiation.

### SOCS6 is a miR-494-3p target

**Figure 5:**
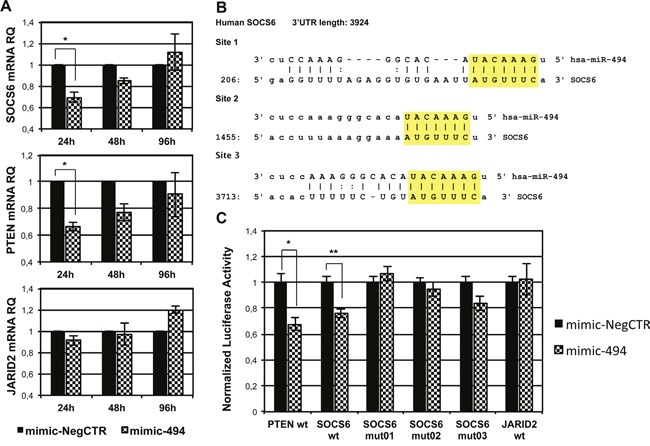
Validation of miR-494-3p/SOCS6 3′UTR interaction **A.** Evaluation of SOCS6, PTEN and JARID2 mRNA expression in CB CD34+ cells overexpressing miR-494-3p by means of qRT-PCR. Transcript levels were assessed 24, 48 and 96 hours after the last nucleofection. Results were normalized to mimic-NegCTR sample and 18s rRNA was selected as endogenous control. Data are reported as RQ mean ± S.E.M of 3 independent experiments. **B.** Representation of the three miR-494-3p predicted binding sites in SOCS6 3′UTR sequence as reported by TargetScanHuman and microRNA.org (www.microrna.org). The seed region of the miRNA is highlighted. **C.** The bar graph represents the results of 3′UTR luciferase reporter assay performed in K562 cell line. Normalized luciferase activity in cells co-nucleofected with miR-494-3p miRNA mimic or miRNA mimic Negative Control and the indicated 3′UTR reporter vector is represented. A sample nucleofected with plasmid containing PTEN 3′UTR was included as positive control, while mutated SOCS6 3′UTR and wild-type JARID2 3′UTR were selected as negative controls. (n=3). Results are reported as mean ± S.E.M. **, p≤0.01; *, p≤0.05

SOCS6 encodes for a protein that belongs to the suppressor of cytokine signaling protein family, a group of molecules involved in the negative regulation of cytokine and of growth factor receptor signaling. In particular, SOCS6 regulates cytokine signaling by interfering with transduction pathways activated by several hematopoietic receptor tyrosine kinases, such as KIT (Mast/stem cell growth factor receptor Kit) and FLT3 (Receptor-type tyrosine-protein kinase FLT3), leading to impaired activation of members of the MAPK pathway [23, 24]. qRT-PCR results showed a significant reduction of SOCS6 mRNA levels in miR-494-3p overexpressing HSPCs 24 hours after the last nucleofection. Similarly, the mRNA levels of phosphatase and tensine homolog (PTEN), chosen as positive control as it is a known miR-494-3p target [25], was decreased in mimic-494 samples, while the expression of jumonji and AT-rich interaction domain containing 2 (JARID2), which was selected as a negative control, was not changed (Figure 5A).

As shown in Figure 5B, SOCS6 3′ untraslated region (3′UTR) displays three putative miRNA recognition elements (MRE), as reported by the target prediction tools TargetScanHuman andmicroRNA.org (www.microrna.org). To validate the direct interaction between miR-494-3p and SOCS6, we employed a reporter vector where the Firefly luciferase gene sequence is located upstream of the wild-type 3′UTR sequence of SOCS6 (SOCS6 wt). As negative controls, we used three different reporter constructs containing a 3 nucleotide substitution in each of the MREs present in SOCS6 3′UTR (i.e. SOCS6 mut01, SOCS6 mut02, SOCS6 mut03) and one reporter plasmid containing JARID2 3′UTR sequence (JARID2 wt). 3′UTR luciferase reporter assays performed with these vectors showed a statistically significant reduction in normalized luciferase activity in K562 cells co-nucleofected with miR-494-3p miRNA mimic and SOCS6 wt reporter construct. This reduction was comparable to that observed in the positive control sample transfected with the plasmid containing PTEN 3′UTR [26] (Figure 5C). Consistently, miR-494-3p failed to induce a significant reduction in normalized luciferase activity when K562 cells were co-nucleofected with SOCS6 3′UTR mutated sequences or JARID2 3′UTR (Figure 5C).

**Figure 6:**
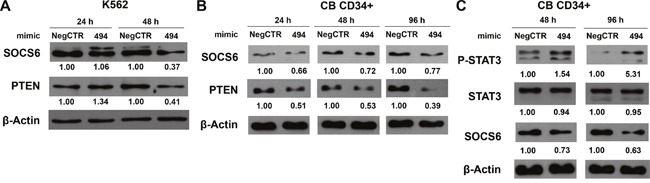
miR-494-3p overexpression reduces SOCS6 expression and enhances STAT3 phosphorilation **A-B.** Western blot analysis of SOCS6 protein levels in whole cell lysates from K562 (A) and CB CD34+ (B) cells overexpressing miR-494-3p at different time-points after mimic nucleofection. SOCS6 protein levels in miR-494-3p overexpressing cells were compared with control samples nucleofected with mimic Negative Control (Neg CTR). β-actin was included as loading control while PTEN was selected as positive control. **C.** Immunoblots representing phospho-STAT3 increase in CB CD34+ cells overexpressing miR-494-3p compared with negative control (Neg CTR) 48 and 96 hours after nucleofection. β-actin was included as loading control.

In order to confirm the downregulation of SOCS6 protein after miR-494-3p overexpression, we performed western blot analysis both in K562 and CB CD34+ cells. While SOCS6 protein was reduced only 48 hours after mimic nucleofection in K562 cells (Figure 6A), SOCS6 protein expression decreased 24, 48 and 96 hours post electroporation in CB CD34+ cells, further supporting the hypothesis that SOCS6 is a direct target of miR-494-3p (Figure 6B). Since SOCS6 has already been shown to act as a negative regulator of signal transducer and activator of transcription 3 (STAT3) signaling [22], we wondered if miR-494-3p overexpression was able to interfere with STAT3 activity. In order to answer this question, we performed western blot analysis, which revealed that miR-494-3p overexpressing cells showed reduced levels of SOCS6 protein as well as higher levels of phospho-STAT3, as depicted in Figure 6C. Altogether, these results demonstrated the direct interaction between miR-494-3p and SOCS6 3′UTR and suggested that miR-494-3p overexpression interferes with STAT3 signaling by inhibiting SOCS6 expression in CD34+ HSPCs.

### SOCS6 silencing supports MK differentiation of HSPCs

Next, we investigated whether SOCS6 downregulation was able to phenocopy the effects on megakaryocytopoiesis seen upon miR-494-3p overexpression in CD34+ cells. To this aim, we inhibited SOCS6 expression by transfecting CD34+ cells with either SOCS6 small interfering RNA (siRNA) (siSOCS6 sample) or negative control siRNA (siNeg CTR). Real-Time qRT-PCR analysis, performed 24 hours after treatment, confirmed the downregulation of mRNA levels in siSOCS6 samples compared to controls (RQ ± S.E.M, 0,4506 ± 0,0430, p<.01).

**Figure 7:**
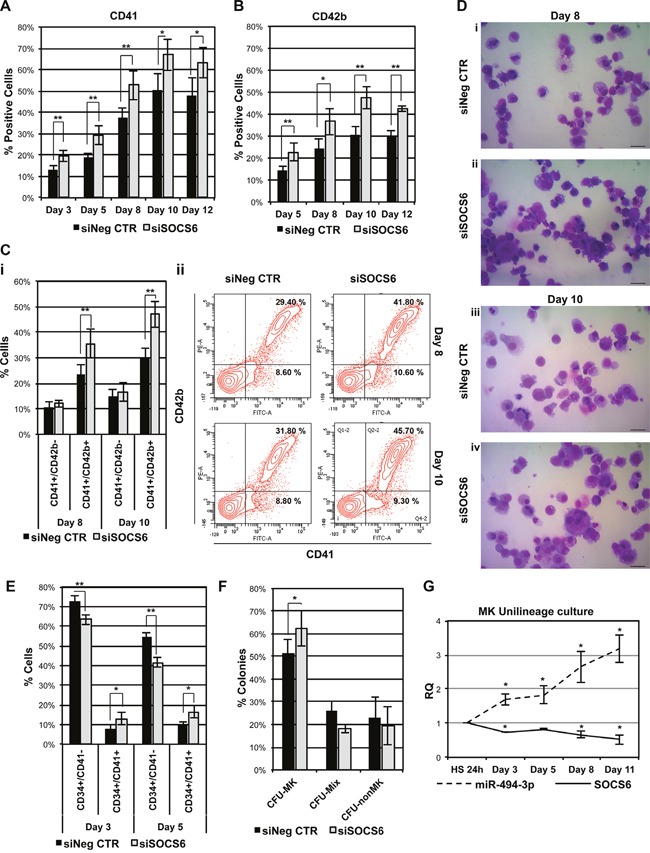
SOCS6 silencing affects MK differentiation of HSPCs **A-B.** Results of the statistical analysis of the percentages of CD41+ and CD42b+ cells evaluated by means of flow cytometry upon SOCS6 silencing in CB CD34+ cells. Marker expression was assessed at days 3, 5, 8, 10 and 12 in cells cultured in serum-free MK unilineage conditions (n=4). **C.** Flow cytometry analysis of cells co-expressing CD41 and CD42b surface antigens at days 8 and 10 of MK unilineage culture (i) (n=4). Sub-panel *ii* displays flow cytometry graphs of a representative experiment. **D.** Morphological analysis of siNeg CTR (i-iii) and siSOCS6 (ii-iv) samples after May-Grünwald-Giemsa staining at days 8 and 10 of MK unilineage culture in a representative experiment. Magnification, x400. Scale bar: 100 μm. **E.** Results of flow cytometry analysis of cells expressing both CD34 and CD41 surface antigens at days 3 and 5 post electroporation in serum free multilineage culture (n=4). **F.** Results of the statistical analysis of collagen-based clonogenic assay of CB CD34+ cells upon SOCS6 silencing. Cells were seeded in semisolid culture medium 24 hours after the last nucleofection and colonies were scored after 11 days (n=3). **G.** miR-494-3p and SOCS6 expression kinetic during MK differentiation. Gene and miRNA expression levels were normalized to cells cultured in the presence of HS for 24 hours after purification (HS 24 sample). 18s rRNA and U6 were used as endogenous controls for gene and miRNA expression evaluation respectively (n=4). Results are reported as mean ± S.E.M. **, p≤0.01; *, p≤0.05 Abbreviations: CFU, colony-forming unit; MK, megakaryocyte; MIX, mixed, nonMK, other than megakaryocyte.

Flow cytometry analysis and methylcellulose-based clonogenic assay demonstrated that SOCS6 downregulation did not affect myeloid or erythroid differentiation of HSPCs cultured in multilineage conditions (data not shown), in agreement with the results obtained upon miR-494-3p overexpression. Since miR-494-3p overexpression promoted MK differentiation in CD34+ cells in unilineage MK cultures, we tested whether SOCS6 silencing could reproduce the same effects. Flow cytometry analysis revealed that SOCS6 silencing was able to induce a significant increase in the percentage of both CD41+ and CD42b+ cell fractions (Figure 7A & 7B). Interestingly, we highlighted an expansion of CD41+/CD42b+ population in siSOCS6 samples, while no variation in CD41+/CD42b- cell fraction was observed (Figure 7C). Accordingly, morphological analysis showed that SOCS6-silenced CD34+ cells generated a higher number of MK precursors compared with the control (Figure 7D). Moreover, SOCS6 silencing caused the expansion of CD34+/CD41+ MK progenitors (Figure 7E), coupled to the increase of the percentage of MK colonies (Figure 7F). Collectively, these results demonstrated that SOCS6 silencing is able to promote MK differentiation of HSPCs, in agreement with the results obtained in miR-494-3p overexpression experiments.

Furthermore, in order to confirm the role of miR-494-3p/SOCS6 axis in MK differentiation, we analyzed their expression levels in CD34+ cells maintained in megakaryocytic unilineage culture conditions. qRT-PCR analysis showed steady upregulation of miR-494-3p expression levels during MK differentiation, which was associated with a reduction of SOCS6 mRNA (Figure 7G).

### miR-494-3p inhibition impairs MK differentiation of PMF CD34+ cells

**Figure 8:**
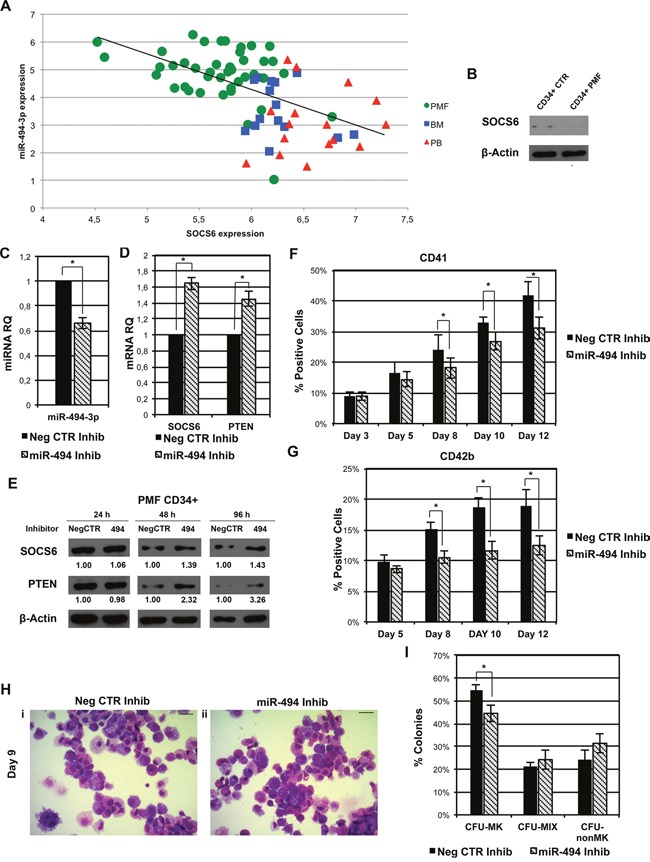
miR-494-3p inhibition impairs MK differentiation of PMF CD34+ cells **A.** Scatter plot representing the correlation between miR-494-3p and SOCS6 expression levels according to the microarray analysis in our initial dataset of 42 PMF samples and 31 healthy donors. The Spearman rank correlation coefficient (r) was used to measure and identify the degree of linear dependence between the ranked variables (r = −0.57, p = 1.12 × 10^−7^). Green circles = PMF CD34+ cells, Red triangles = PB CD34+ cells, Blue squares = BM CD34+ cells. Abbreviations: PMF, primary myelofibrosis; PB, peripheral blood; BM, bone marrow; CTR, control. **B.** Western blot analysis of SOCS6 protein levels in PMF CD34+ cells compared to CB CD34+ cells. β-actin was included as loading control. **C-D.** Expression levels of miR-494-3p, SOCS6, and PTEN in PMF CD34+ cells after miRNA inhibition evaluated by means of qRT-PCR. Data are reported as RQ mean ± S.E.M (n=3). **E.** Immunoblots representing SOCS6 protein levels in whole cell lysates from PMF CD34+ cells after miR-494-3p inhibition. SOCS6 protein levels were evaluated 24, 48 and 96 hours after miR-494-3p inhibitors nucleofection and were compared with control samples electroporated with Negative Control inhibitors (NegCTR). β-actin was included as loading control while PTEN was selected as positive control. **F-G.** Results of flow cytometry analysis of the percentages of CD41+ (F) and CD42b+ (G) cells evaluated at days 3, 5, 8, 10 and 12 in serum-free MK unilineage conditions (n=3). **H.** Morfological analysis of Neg CTR Inhib (i) and miR-494 Inhib (ii) samples after MGG staining at day 9 of MK unilineage culture in a representative experiment. Magnification, x400. Scale bar: 100 μm. **I.** Results of the statistical analysis of collagen-based clonogenic assay of PMF CD34+ cells upon miR-494-3p inhibition. Cells were seeded in semisolid culture medium 24 hours after the last nucleofection and colonies were scored after 11 days (n=3). Results are reported as mean ± S.E.M. *, p≤0.05. Abbreviations: CFU, colony-forming unit; MK, megakaryocyte; MIX, mixed; nonMK, other than megakaryocyte.

Finally, to gain further insight into the role of miR-494-3p/SOCS6 axis in the pathogenesis of PMF, we looked for a possible correlation between miR-494-3p and SOCS6 expression levels in our dataset of 42 PMF samples and 31 healthy donors. Interestingly, we found that SOCS6 expression was negatively correlated to miR-494-3p expression (r = −0.57, p = 1.12 × 10^−7^) (Figure 8A) and western blot analysis confirmed SOCS6 protein downregulation in PMF CD34+ cells (Figure 8B).

Furthermore, in order to investigate if the downregulation of miR-494-3p could reduce the production of MKs in PMF CD34+ cells, we transfected them with a miR-494-3p inhibitor (miR-494 Inhib) or a negative control inhibitor (Neg CTR Inhib). The downregulation of miR-494-3p (Figure 8C) caused the increase in SOCS6 and PTEN expression both at mRNA and protein levels (Figure 8D & 8E).

Flow cytometry analysis showed that the downregulation of miR-494-3p was able to reduce both the CD41+ and CD42b+ fractions (Figure 8F & 8G), whereas a reduction in MK precursors was observed by morphological analysis after miR-494-3p inhibition (Figure 8H). Finally, we observed a statistically significant decrease in the percentage of MK colonies in miR-494-3p Inhib sample (Figure 8I).

Altogether these results demonstrate that miR-494-3p inhibition in PMF CD34+ cells led to SOCS6 upregulation and impairs MK differentiation suggesting the involvement of miR-494-3p/SOCS6 axis in PMF pathogenesis.

## DISCUSSION

A growing body of experimental evidence has been supporting a key role for miRNAs in tumorigenesis, both for solid cancers and for hematological malignancies [27, 28]. Recently, our group showed how miRNAs may contribute to the pathogenesis of PMF. In particular, we analyzed and integrated gene and miRNA expression profiles in PMF CD34+ cells to uncover networks of miRNA-mRNA interaction. Biologically relevant network nodes may be composed either by deregulated genes targeted by many aberrantly expressed miRNAs or by single miRNAs targeting multiple genes. While the first type of network highlights the relevance of the targeted gene, the latter emphasizes the role of the miRNA as a central player in the pathogenetic mechanisms. Thus, we sought to find an aberrantly expressed miRNA targeting many genes in our dataset [21].

In the present study, we found a network involving the most upregulated miRNAs in PMF CD34+ cells and their common downregulated predicted targets. Among the most upregulated miRNAs, miR-494-3p emerges as being associated to the highest number of downregulated target mRNAs. In particular, 86 DEGs were found to be miR-494-3p predicted targets *in silico*. Supporting a possible role for miR-494-3p in PMF pathogenesis, among its 86 downregulated targets there are several genes that have been described as tumor suppressor genes (CUL3 [29], KLF11 [30], TRAF3 [31] and SOCS6 [32]) as well as genes whose low expression has been associated with the development of myeloid malignancies such as ARID4B [33], PURB [34], ELF2 [35] and RAD23B [36]. Furthermore, among the miR-494-3p downregulated targets, NR2C1, WIP1, HLF and HMGB3 encode for proteins involved in the regulation of hematopoietic stem cell self-renewal and differentiation [37–40]. However, the role of miR-494-3p in tumorigenesis has generally been reported as controversial. Nonetheless, several authors have described it as an oncomiR in several neoplastic contexts, such as in retinoblastoma [41], hepatocellular carcinoma [42] and in non-small cell lung cancer [25]. In hematopoietic malignancies, the overexpression of miR-494-3p has been reported in various forms of lymphomas such as follicular lymphoma [43], classical Hodgkin lymphoma [44], nasal natural killer cell lymphoma [45]. More recently, the overexpression of miR-494-3p in CD34+ cells from PMF patients has been described by our group [21]. Therefore, we wondered if miR-494-3p could be able to alter HSPC fate decisions and thus could be involved in PMF pathogenesis.

To this end, we performed miR-494-3p overexpression experiments in normal CD34+ cells demonstrating that its upregulation enforces MK differentiation while myeloid or erythroid differentiation are not affected. In particular, immunophenotypic and morphological analysis revealed that miR-494-3p overexpression promotes megakaryocytopoiesis in HSPCs leading to the expansion of both early and late MK precursors in liquid and semisolid culture.

Therefore, to go even deeper into the molecular mechanisms by which miR-494-3p might be able to support megakaryocytopoiesis, we performed gene expression profiling of CD34+ cells overexpressing miR-494-3p, to specifically find downregulated genes predicted as putative target of this miRNA. Since we hypothesized that miR-494-3p upregulation might contribute to the aberrant megakaryocytopoiesis in PMF patients, we sought deregulated genes that were similarly downregulated both after miR-494-3p overexpression and in PMF CD34+ cells. Among them, SOCS6 was selected since it was predicted with particularly high likelihood to be a target of miR-494-3p according to TargetScanHuman prediction tool.

In order to confirm the predicted interaction, we performed luciferase reporter assay and western blot analysis, which demonstrated that SOCS6 is a real target of miR-494-3p in hematopoietic cells.

SOCS6 belongs to the family of suppressor of cytokine signaling (SOCS) proteins which act as negative regulators of cytokine and growth factor receptor signaling [46]. In particular, SOCS6 has been reported to interfere with the signal transduction of key hematopoietic receptor tyrosine kinases. Indeed, SOCS6 associates to KIT and FLT3 upon ligand stimulation to induce receptor ubiquitination with consequent reduction of their protein levels, ultimately leading to the impaired activation of ERK1/2 and p38 MAP kinases [23, 24, 47]. Furthermore, SOCS6 regulates the JAK/STAT signaling pathway by decreasing STAT3 nuclear protein levels and by inhibiting STAT3 activation reducing the amount of phosphorilated protein [22, 48]. Of particular interest, TPO promotes megakaryocytopoiesis in HSPCs through the binding to its receptor TPO-R (Thrombopoietin receptor) that leads to the activation of JAK2 protein kinase, which in turn phosphorilates and activates STAT3 [49]. Of further relevance, constitutive activation of the JAK/STAT pathway caused by the presence of mutations in MPL (i.e. the gene encoding for TPO-R) and CALR genes represents an important pathogenetic event in PMF onset and development [13, 14, 50]. Since miR-494-3p inhibits SOCS6, which is a negative regulator of STAT3, a key player in megakaryocytopoiesis, we therefore hypothesized that STAT3 would be more active when miR-494-3p expression is upregulated. In keeping with these observations, our results demonstrated that miR-494-3p overexpression in CD34+ HSPCs indeed reinforced STAT3 signaling by reducing SOCS6 expression, thus increasing the amount of phospho-STAT3. Altogether, these observations compelled us to investigate the role of SOCS6 in normal CD34+ cell differentiation.

First, we silenced SOCS6 expression in CD34+ cells by using a siRNA-based approach in order to establish if SOCS6 silencing might reproduce the effects of miR-494-3p overexpression. Thus, we inhibited SOCS6 expression during the first days of liquid culture, corresponding to the early stages of HSPC commitment *in vitro*. Immunophenotypic and morphological analysis, as well as collagen-based clonogenic assays, demonstrated that SOCS6 downregulation phenocopies the same effects on CD34+ cell differentiation observed in miR-494-3p overexpression experiments. Indeed, SOCS6 downregulation supported megakaryocytopoiesis but had no effects on myeloid or erythroid differentiation. Our results showed also that SOCS6 silencing affected both early and late stages of MK differentiation. Furthermore, we analyzed miR-494-3p and SOCS6 expression during MK differentiation of CD34+ cells. Analysis of the expression kinetics showed that miR-494-3p is upregulated while SOCS6 mRNA is downregulated when normal CD34+ cells are forced to megakaryopoiesis *in vitro*. Altogether, our results demonstrated that miR-494-3p acts through the downregulation of its target SOCS6 and promotes MK differentiation *in vitro*. Finally, to confirm that the newly described miR-494-3p/SOCS6 axis may play role in the pathogenesis of PMF, we highlighted the negative correlation between miR-494-3p and SOCS6 expression in CD34+ PMF samples. Furthermore, we demonstrated that the silencing of miR-494-3p in PMF CD34+ cells, which overexpress this miRNA, induced SOCS6 upregulation and impaired MK overproduction.

One of the main features characterizing the bone marrow of PMF patients is an excessive number of MKs, which have been proposed to be the key cells leading to the fibrotic phenotype through a cytokine storm [6]. Therefore, it is of primary importance to explore the biological mechanisms that drive this MK hyperplasia. Many and different somatic mutations have been reported to be carried from PMF patients, who may have complex combination of such lesions. Analysis of miRNA and mRNA expression is therefore important as it may help to find common pathways that are deregulated and shared by these subgroups of patients. Integrative analysis of aberrantly expressed miRNAs and genes in CD34+ cells and functional validation experiments highlighted a possible role for miR-494-3p and its target SOCS6 in the pathogenesis of PMF.

Overall, these data suggest that miR-494-3p/SOCS6 axis is an important player during the physiological differentiation of HSPCs due to its ability to enforce the lineage commitment decision towards megakaryocytopoiesis. Of particular interest, our results support the hypotesis that miR-494-3p altered expression in PMF CD34+ cells contributes to the development of MK hyperplasia through the downregulation of SOCS6.

## MATERIALS AND METHODS

### Ethics statement

Human CD34+ cells were purified upon donor's informed written consent from umbilical cord blood samples, collected after normal deliveries, according to the institutional guidelines for discarded material.

Primary myelofibrosis CD34+ cells were isolated from the peripheral blood (PB) of 3 patients diagnosed with PMF in a typical fibrotic phase of the disease according to the recently updated World Health Organization (WHO) criteria [1]. All subjects provided informed written consent, and the study was performed under the local Institutional Review Board's approved protocol (Florence: approval date: April 22, 2011, approval file number # 2011/ 0014777; Pavia: approval date: February 24, 2011, file number #174). The study was conducted in accordance with the Declaration of Helsinki.

### Gene and miRNA expression profiles integrative analysis

Gene and miRNA expression profiles of CD34+ cells from 42 PMF and 31 healthy donors (n=15 BM, n=16 PB) were previously reported and submitted to a public repository (GEO series GSE41812 and GSE53482) [21]. Differentially expressed genes (DEGs) and miRNAs (DEMs) were here selected by using the analysis of variance (ANOVA) module included in the Partek GS package. In particular, we considered as differentially expressed all the probe sets with a fold change contrast (FC) ≥1.5 and false discovery rate (q value) <0.05 for both genes and miRNAs in the pairwise comparison of PMF vs controls.

In silico integrative analysis was performed by using QIAGEN's Ingenuity Pathway Analysis software (Ingenuity Systems; Redwood City, CA,http://www.ingenuity.com/ingenuity), as previously described [21].

### CD34+ cell purification

Human CD34+ cells were purified from CB or PB samples from PMF patients as previously described [21]. Purified CD34+ cells were cultured in 24-well plates at 5×10^5^/ml in Iscove's modified Dulbecco's medium (IMDM, Euroclone) containing 20% HS (Bio-Whittaker), interleukin-3 (IL-3) (10 ng/ml), interleukin-6 (IL-6) (10 ng/ml), thrombopoietin (TPO) (20 ng/ml), stem cell factor (SCF) (50 ng/ml) and Flt3-ligand (FLT3L) (50 ng/ml), (all from Miltenyi Biotec; Auburn, CA, USA) and electroporated 24 hours later.

### Electroporation of CD34+ cells

Human CD34+ cells were nucleofected by using the 4DNucleofector™ System (Lonza) as previously reported [21], with minor changes. With regards to miR-494-3p overexpression experiments, each sample was nucleofected twice, once every 24 hours, with mirVana™ miRNA mimics (Thermo Fisher Scientific Inc) starting from the day upon CD34+ cell purification. For each electroporation, 3-4×10^5^ CD34+ cells were nucleofected with 3 μg (0,21 nmol) of mirVana™ miR-494-3p miRNA mimic (mimic-494) using the preset pulsing program DS112. To account for nonspecific effects given by electroporation of small RNAs, CD34+ cells transfected with mirVana miRNA mimic Negative Control #1 (mimic-NegCTR) were used as controls.

Similarly, Silencer^R^ Select small interfering RNA (siRNA) (Thermo Fisher Scientific Inc) transfection was performed as follows [21]. Briefly, CD34+ cells were nucleofected 3 times, once every 24 hours, using 3 μg of Silencer^R^ Select siRNA against human SOCS6 (Supplementary Table 5) or a non-targeting siRNA as a negative control (Silencer^R^ Select Negative Control #2 siRNA; Thermo Fisher Scientific Inc) by using the previously mentioned electroporation protocol DS112.

For miRNA inhibition experiments, PMF CD34+ cells were nucleofected 3 times using the same pulsing protocol, once every 24 hours, with 1 μg (0,21 nmol) of *in vivo* LNA™ miR-494-3p inhibitor or the same quantity of a scramble negative control inhibitor (Exiqon).

SOCS6 or miR-494-3p expression were analyzed 24 hours upon the last nucleofection.

### RNA extraction and qRT-PCR

Total cellular RNA was harvested from 5×10^4^ cells from each sample using the miRNeasy Micro RNA isolation kit (QIAGEN), according to the manufacturer's instructions and as previously described [51].

Relative quantification (RQ) of mRNA and miRNA expression levels was performed as previously described [51]. 18S ribosomal RNA (18S rRNA) or glyceraldehyde-3-phosphate dehydrogenase (GAPDH) were used as housekeeping genes for mRNA expression RQ, while U6 snRNA was used as housekeeping control for RQ of miRNA expression levels.

### Gene expression profiling (GEP)

GEP was performed on RNA samples isolated from CD34+ cells transfected with both miR-494-3p miRNA mimics or miRNA mimic Negative control 24 hours after 2 nucleofections from three independent experiments. mRNA was processed as previously described [51]. Robust multiarray average (RMA) procedure was used to perform probe level normalization and conversion into expression values [52]. DEGs were then selected following a supervised approach with the analysis of variance (ANOVA) module supplied by the Partek GS. 6.6 Software Package (http://www.partek.com). We considered as differentially expressed all the probesets with a fold change contrast ≥1.5 in the pairwise comparison between mimic-494 and mimic-NegCTR samples and a p-value ≤0.05.

Raw and normalized GEP data have been submitted to the NCBI's Gene Expression Omnibus (GEO) public repository (http://www.ncbi.nlm.nih.gov/geo; series GSE85250).

### CD34+ cell-culture conditions

Liquid cultures to assess the differentiation potential were set up 24 hours after the last nucleofection. Briefly, CD34+ cells were seeded 3-5×10^5^/mL in IMDM with 20% HS in order to set up a multilineage cell culture (IL-6, 10 ng/mL; IL-3, 10 ng/mL; TPO, 20 ng/mL; SCF, 50 ng/mL; Flt3L, 50 ng/mL; all cytokines from Miltenyi Biotec). To obtain MK and erythroid differentiation *in vitro*, cells were also cultured in IMDM supplemented with 20% BIT 9500 serum substitute (bovine serum albumin, insulin, and transferrin; StemCell Technologies) with the same afore-mentioned cytokine cocktail. Unilineage cultures were set up by seeding 3-5×10^5^ cells/mL in IMDM with the addition of 20% BIT and TPO 100 ng/mL in order to induce MK differentiation (Miltenyi Biotec) [51].

In order to study the expression kinetics of miR-494-3p during MK differentiation, CD34+ cells were cultured in 24-well plates at a density of 3-5×10^5^/ml in the same condition described above. After a first phase of expansion, 24 hours after purification, 5×10^4^ cells were lysed for RNA extraction (HS 24h) and the remaining cells were seeded (5×10^5^/ml) in MK unilineage culture conditions. The medium was replaced every 3 days. Cell differentiation was monitored by morphological analysis of May-Grünwald-Giemsa (MGG) stained cytospins and by flow cytometry analysis of differentiation marker expression. miR-494-3p and SOCS6 expression levels were detected by qRT-PCR at different time points (i.e. days 3, 5, 8 and 12) after seeding the cells in MK unilineage cultures.

### Morphological and immunophenotypic analysis

Differentiation of CD34+ cells was assessed by morphological analysis of stained cytospins and by flow cytometry analysis of differentiation marker expression (CD34, CD38, CD66b, CD15, myeloperoxidase (MPO), CD14, CD163, Glycophorin A (GPA), CD41, CD42b) at day 3, 5, 8, 10, and 12 after the last nucleofection. The following monoclonal antibodies (MoAbs) were used for flow cytometry analysis: fluorescein isothiocyanate (FITC)-conjugated mouse anti-human CD34 MoAb; allophycocyanin (APC)-conjugated mouse anti-human CD34 MoAb; FITC-conjugated mouse anti-human CD66b MoAb; FITC-conjugated mouse anti-human CD15 MoAb; phycoerythrin (PE)-conjugated mouse anti-human CD14 MoAb; APC-conjugated mouse anti-human CD163 MoAb; PE-conjugated mouse anti-human CD42b MoAb (all from Miltenyi Biotech); FITC-conjugated mouse anti-human CD41 MoAb, and PE-conjugated mouse anti-human GPA MoAb (all from Dako; Milano, Italia;http://www.dako.com) and FITC-conjugated mouse anti-human MPO MoAb (from BD Biosciences; San Jose, CA USA). For the MPO intracellular staining, a previous fixation and permeabilization step is required, therefore we employed the Fix&Perm kit (ThermoFisher Scientific). After staining, cells were analyzed by using a BD FACSCanto II (BD Biosciences; San Jose, CA USA). At least 10,000 events were counted for each sample to ensure statistical relevance.

Images of MGG stained cytospins were captured by using an Ax10scopeA1 microscope equipped with an AxioCam ERc 5S Digital Camera and Axion software 4.8 (all Carl Zeiss MicroImaging Inc.; Thornwood, NY, USA). The images were then processed with Adobe Photoshop 7.0 software.

### Methylcellulose and collagen clonogenic assays

Clonogenic capacity of nucleofected CD34+ cells was assessed by setting up two different assays. The methylcellulose-based clonogenic assay was carried out by plating CD34+ cells in MethoCult™ GF H4434 (StemCell Technologies Inc.; Vancouver), as previously described [53]. In addition, MK colony forming units (CFU-MK) were assayed in collagen-based medium, using a commercial MK assay detection kit (MegaCult-C; StemCell Technologies Inc.) as previously reported [54]. Briefly, 24 hours after the last nucleofection 2.5 × 10^3^ CB CD34+ cells and 3.75 × 10^3^ PMF CD34+ cells were seeded in each chamber of a double-chamber slide (0.75 mL per-chamber). The employed medium contained: collagen (1.1 mg/ml), bovine serum albumin (1%), bovine pancreatic insulin (0.01 mg/ml), human transferrin (iron-saturated) (0.2 mg/ml), and the following human recombinant cytokines: TPO (50 ng/ml), IL-3 (10 ng/ml) and IL-6 (10 ng/ml). After 11 days of incubation at 37°C the chamber slides were fixed in methanol/acetone solution and MK colonies were stained using a primary monoclonal anti-CD41 (GPIIb/IIIa) antibody. MK colonies were identified using an alkaline phosphatase/naphthol detection system (all from StemCell Technologies), while nuclei of all the cells, regardless of their lineage, were counterstained with Evans Blue. CD41- positive colonies were scored as CFU-MK and subdivided by size in small (3-21 cells), medium (21-49 cells) and large (>50 cells) colonies which reflects the maturation stage of the progenitor giving rise to each colony.

### 3′ untranslated region (3′UTR) luciferase reporter assays

Empty luciferase reporter constructs (pEZX-MT01) or plasmids containing wild-type and mutated full-length 3′UTR from human SOCS6 (RefSeq NM_004232.3), human phosphatase and tensine homolog (PTEN) (RefSeq NM_000314.4) and human JARID2 (RefSeq NM_004973.2) transcripts were all purchased from Labomics (Genecopoeia; MD, USA). Further details are presented in Supplementary Table 6. Luciferase assay and its following normalization were performed as previously reported [21].

### Western blot

Protein levels were assessed by means of Western Blot Analysis in both CB CD34+ cells and K562 cells (ATCC) overexpressing miR-494-3p, and in PMF CD34+ cells after miRNA inhibition. Briefly, cells were harvested 24, 48 and 96 hours after the last nucleofection, washed twice with cold phosphate-buffered saline (PBS) and lysed in 50 mM Tris (tris(hydroxymethyl) aminomethane)-Cl (pH 7.4), 150 mM NaCl, 1% Nonidet P-40, 10 mM KCl, 1 mM EDTA, 20 mM NaF, 0.25% Na deoxycholate, 5 mM dithiothreitol (DTT). Protease inhibitors (Roche, Indianapolis, IN, USA Complete, catalog #1697498) and phosphatase inhibitors (ThermoFisher Scientific) were added to the lysis buffer. Total cellular lysates (20μg for each sample) were loaded and separated on 10% SDS-polyacrylamide gel and then transferred on a nitrocellulose membrane. To visualize loading and transfer, Ponceau staining has been performed. Membranes were then pre-blocked in a blocking solution of 0.1% tris-buffered saline Tween 20 (TBS-T) containing 5% Bovine Serum Albumin (BSA) and then incubated with the following primary antybodies: mouse monoclonal anti-SOCS6 antibody (Abcam, Cambridge, UK, catalog #ab56516; 1:500 dilution at 4°C overnight), mouse monoclonal anti-PTEN antibody (Santa Cruz Biotechnology, Inc, Dallas, Texas, USA; catalog #sc-7974; 1:500 dilution for 1h and 45 minutes at room temperature (RT)), rabbit monoclonal anti-STAT3 antibody (Cell Signaling Technology, Inc., catalog #4904; 1:1000 dilution at 4°C overnight), rabbit monoclonal anti-phospho-STAT3 antibody (phospho-Ser727, Cell Signaling Technology, Inc., catalog #9134; 1:250 dilution at 4°C overnight) and with rabbit polyclonal anti-β-actin primary antibody (Thermo Fisher Scientific Inc, catalog #PA1-16889; 1:2000 dilution for 1 hour at RT). The blots were washed for three times with TBS-T and then incubated with 1:5000 dilution of horseradish peroxidase (HRP) conjugated goat anti-mouse (Santa Cruz Biotechnology, Santa Cruz, CA, USA catalog #sc-2005) and/or 1:1000 dilution of HRP-conjugated goat anti rabbit secondary antibody (Thermo Fisher Scientific Inc., catalog #32460) for 1h at RT. After three successive washes with TBS-T, BM chemiluminescence Blotting Substrate (POD) (Roche) was used for protein detection.

### Statistical analysis

All the statistics used for data analysis in overexpression/silencing experiments and 3′UTR luciferase reporter assays were based on 2-tailed Student t-tests for average comparisons in paired samples. Data were then analyzed by means of Microsoft Excel (Microsoft Office, 2008 release) and have been reported as mean ± standard error of the mean (S.E.M). A p-value less than 0.05 was considered significant.

Correlation between miR-494-3p and SOCS6 expression was displayed by means of Spearman's correlation.

## SUPPLEMENTARY MATERIALS FIGURES AND TABLES




