# Supplementary material for: miR-494-3p overexpression promotes megakaryocytopoiesis in primary myelofibrosis hematopoietic stem/progenitor cells by targeting SOCS6
Source: Oncotarget. 2017 Feb 9;8(13):21380–97. doi: 10.18632/oncotarget.15226 (PMC5400591; doi:10.18632/oncotarget.15226)
Supplement: Supplementary file 1 [file oncotarget-08-21380-s001.pdf]

miR-494-3p overexpression promotes megakaryocytopoiesis in primary myelofibrosis hematopoietic stem/progenitor cells by targeting SOCS6

SUPPLEMENTARY FIGURE AND TABLES

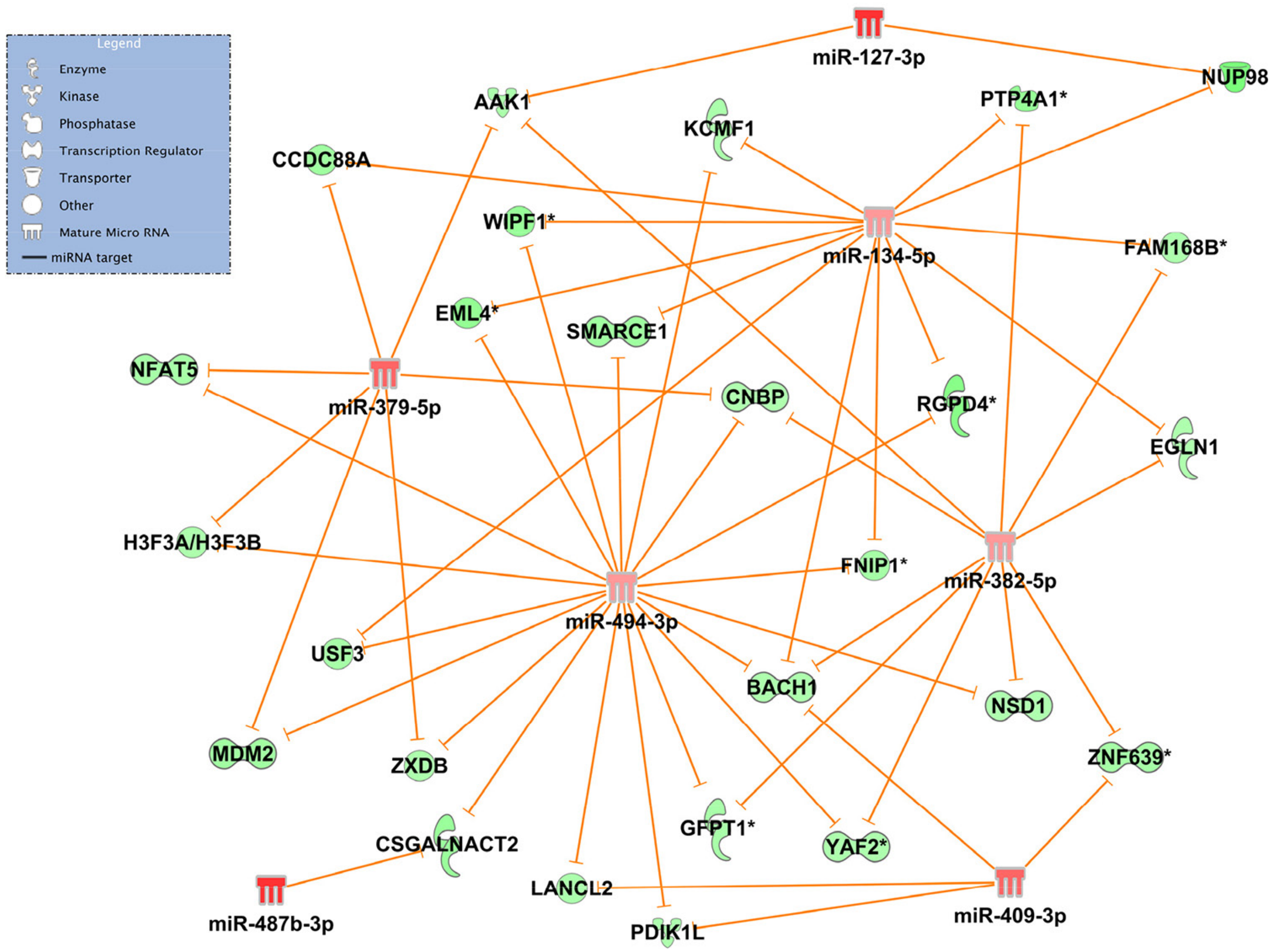

Supplementary Figure 1: Regulatory network including the most upregulated miRNAs and their common targets. Visualization of the regulatory network which includes the most upregulated miRNAs in PMF compared to normal CD34+ cells and their common targets according to IPA integrative analysis. Red filling means upregulation, and green filling indicates downregulation.

Supplementary Table 1: PMF downregulated miR-494-3p predicted targets

See Supplementary File 1

Supplementary Table 2: PMF downregulated miRNA targets

See Supplementary File 1

Supplementary Table 3: Differentially expressed genes (DEGs) upon miR-494-3p overexpression

See Supplementary File 1

Supplementary Table 4: miR-494-3p downregulated predicted targets

See Supplementary File 1

Supplementary Table 5: SOCS6 small interfering RNA (siRNA)

RefSeq Accession Number	Gene Symbol	Full Gene Name	Gene ID	siRNA ID	Sense siRNA Sequence	Antisense siRNA Sequence	Targeted Exon
NM_004232.3	SOCS6	suppressor of cytokine signaling 6	9306	s17777	GCUGCGAUAUCAACGGUGAtt	UCACCGUUGAUAUCGCAGCtg	2

In table it is reported the sequence of siRNA targeting SOCS6 mRNA employed for silencing experiments in CD34+ cells.

Supplementary Table 6: Luciferase reporter constructs

Gene Symbol	Vector	RefSeq	Vector ID	Insert
/	pEZX-MT01	/	CmiT000001-MT01	3'UTR-less
SOCS6	pEZX-MT01	NM_004232.3	HmiT022497-MT01	3'UTR SOCS6
SOCS6	pEZX-MT01	NM_004232.3	HmiT022497-MT01-01	mutant 3'UTR SOCS6 (Binding SITE 1 for miR-494-3p, TTT->CGC)
SOCS6	pEZX-MT01	NM_004232.3	HmiT022497-MT01-02	mutant 3'UTR SOCS6 (Binding SITE 2 for miR-494-3p, TTT->CGC)
SOCS6	pEZX-MT01	NM_004232.3	HmiT022497-MT01-03	mutant 3'UTR SOCS6 (Binding SITE 3 for miR-494-3p, TTT->CGC)
PTEN	pEZX-MT01	NM_000314.4	HmiT015535-MT01	3'UTR PTEN
JARID2	pEZX-MT01	NM_004973.2	HmiT009852-MT01	3'UTR JARID2

This table represents the list of luciferase reporter constructs used for 3' UTR luciferase reporter assays.
