# Supplementary material for: miR-494-3p overexpression promotes megakaryocytopoiesis in primary myelofibrosis hematopoietic stem/progenitor cells by targeting SOCS6
Source: Oncotarget. 2017 Feb 9;8(13):21380–97. doi: 10.18632/oncotarget.15226 (PMC5400591; doi:10.18632/oncotarget.15226)
Supplement: Supplementary file 2 [file oncotarget-08-21380-s002.docx]

**Table 1 : PMF downregulated miR-494-3p predicted targets.** This is the list of 86 downregulated predicted targets of miR-494-3p in PMF CD34+ cells according to integrative analysis performed by means of Ingenuity Pathway Analysis.

| **Probeset ID** | **Entrez Gene** | **Gene Symbol** | **Gene Title** | **RefSeq Transcript ID** | **False Discovery Rate** | **Fold-Change (PMFvsCTR)** | **Source** | **Confidence** |
| --- | --- | --- | --- | --- | --- | --- | --- | --- |
| 11740338_a_at | 3267 | AGFG1 | ArfGAP with FG repeats 1 | NM_001135187 NM_001135188 NM_001135189 NM_004504 | 9,14734E-09 | -2,22516 | TargetScan Human | High (predicted) |
| 11721879_s_at | 51742 | ARID4B | AT rich interactive domain 4B (RBP1-like) | NM_016374 NM_031371 | 1,08791E-06 | -1,68441 | TargetScan Human | High (predicted) |
| 11742557_s_at | 10776 | ARPP19 | cAMP-regulated phosphoprotein, 19kDa | NM_006628 | 1,42594E-05 | -1,64984 | TargetScan Human | High (predicted) |
| 11726369_at | 571 | BACH1 | BTB and CNC homology 1, basic leucine zipper transcription factor 1 | NM_001011545 NM_001186 NM_206866 NR_027655 | 0,00223504 | -1,55803 | TargetScan Human | High (predicted) |
| 11755179_x_at | 375341 | C3orf62 | chromosome 3 open reading frame 62 | NM_198562 | 4,49417E-07 | -1,63559 | TargetScan Human | High (predicted) |
| 11734717_a_at | 79872 | CBLL1 | Cas-Br-M (murine) ecotropic retroviral transforming sequence-like 1 | NM_024814 NR_024199 | 1,06006E-07 | -1,62211 | TargetScan Human | High (predicted) |
| 11754992_a_at | 1105 | CHD1 | chromodomain helicase DNA binding protein 1 | NM_001270 | 1,33276E-09 | -2,218 | TargetScan Human | High (predicted) |
| 11754038_a_at | 7555 | CNBP | CCHC-type zinc finger, nucleic acid binding protein | NM_001127192 NM_001127193 NM_001127194 NM_001127195 NM_001127196 NM | 0,00798281 | -1,53682 | TargetScan Human | High (predicted) |
| 11723281_s_at | 58487 | CREBZF | CREB/ATF bZIP transcription factor | NM_001039618 NR_028024 NR_028025 NR_028026 NR_028027 | 6,56972E-08 | -1,77945 | TargetScan Human | High (predicted) |
| 11732701_a_at | 55454 | CSGALNACT2 | chondroitin sulfate N-acetylgalactosaminyltransferase 2 | NM_018590 | 1,03357E-07 | -1,93379 | TargetScan Human | Moderate (predicted) |
| 11716505_a_at | 8452 | CUL3 | cullin 3 | NM_003590 | 5,81402E-12 | -1,56486 | TargetScan Human | High (predicted) |
| 11756266_a_at | 26999 | CYFIP2 | cytoplasmic FMR1 interacting protein 2 | NM_001037332 NM_001037333 NM_014376 | 4,4313E-06 | -2,34575 | TargetScan Human | High (predicted) |
| 11759340_at | 163486 | DENND1B | DENN/MADD domain containing 1B | NM_001142795 NM_144977 | 9,61799E-09 | -1,58389 | TargetScan Human | Moderate (predicted) |
| 11758454_s_at | 201627 | FAM116A | family with sequence similarity 116, member A | NM_152678 | 6,09062E-18 | -1,82239 | TargetScan Human | High (predicted) |
| 11718109_s_at | 1738 | DLD | dihydrolipoamide dehydrogenase | NM_000108 | 1,84372E-05 | -1,52736 | TargetScan Human | High (predicted) |
| 11724727_a_at | 1983 | EIF5 | eukaryotic translation initiation factor 5 | NM_001969 NM_183004 | 4,27336E-05 | -1,56265 | TargetScan Human | High (predicted) |
| 11760507_at | 1998 | ELF2 | E74-like factor 2 (ets domain transcription factor) | NM_006874 NM_201999 | 1,73036E-05 | -1,68491 | TargetScan Human | High (predicted) |
| 11722752_a_at | 91748 | C14orf43 | chromosome 14 open reading frame 43 | NM_001043318 NM_194278 | 6,68281E-05 | -1,84632 | TargetScan Human | High (predicted) |
| 11716653_x_at | 27436 | EML4 | echinoderm microtubule associated protein like 4 | NM_001145076 NM_019063 | 1,78562E-05 | -2,03988 | TargetScan Human | High (predicted) |
| 11760585_at | 56946 | C11orf30 | chromosome 11 open reading frame 30 | NM_020193 | 2,58844E-08 | -1,85381 | TargetScan Human | High (predicted) |
| 11734858_at | 56605 | ERO1LB | ERO1-like beta (S. cerevisiae) | NM_019891 | 1,13625E-10 | -2,27536 | TargetScan Human | High (predicted) |
| 11754677_a_at | 26049 | FAM169A | family with sequence similarity 169, member A | NM_015566 | 0,000119104 | -1,64828 | TargetScan Human | High (predicted) |
| 11725457_at | 254170 | FBXO33 | F-box protein 33 | NM_203301 | 1,88172E-07 | -2,1049 | TargetScan Human | High (predicted) |
| 11741731_a_at | 55030 | FBXO34 | F-box protein 34 | NM_017943 NM_152231 | 2,28146E-08 | -1,9324 | TargetScan Human | High (predicted) |
| 11731054_s_at | 96459 | FNIP1 | folliculin interacting protein 1 | NM_001008738 NM_133372 | 1,37753E-05 | -1,71509 | TargetScan Human | High (predicted) |
| 11720223_at | 2673 | GFPT1 | glutamine--fructose-6-phosphate transaminase 1 | NM_002056 | 2,85991E-09 | -1,65345 | TargetScan Human | High (predicted) |
| 11743694_at | 94239 | H2AFV | H2A histone family, member V | NM_012412 NM_138635 NM_201436 NM_201516 NM_201517 | 3,76733E-08 | -2,31476 | TargetScan Human | Moderate (predicted) |
| 11758593_s_at | 3021 | H3F3B | H3 histone, family 3B (H3.3B) | NM_005324 | 4,88303E-05 | -1,55932 | TargetScan Human | High (predicted) |
| 11724271_a_at | 3131 | HLF | hepatic leukemia factor | NM_002126 | 0,0205434 | -1,51572 | TargetScan Human | High (predicted) |
| 11754183_s_at | 3149 | HMGB3 | high-mobility group box 3 | NM_005342 | 4,19423E-19 | -2,50522 | TargetScan Human | High (predicted) |
| 11728784_a_at | 54556 | ING3 | inhibitor of growth family, member 3 | NM_019071 NM_198267 | 2,32049E-05 | -1,5805 | TargetScan Human | High (predicted) |
| 11726895_a_at | 11213 | IRAK3 | interleukin-1 receptor-associated kinase 3 | NM_001142523 NM_007199 | 6,74283E-05 | -1,87471 | TargetScan Human | High (predicted) |
| 11757969_x_at | 3727 | JUND | jun D proto-oncogene | NM_005354 | 0,00278443 | -1,50055 | TargetScan Human | High (predicted) |
| 11718518_at | 56888 | KCMF1 | potassium channel modulatory factor 1 | NM_020122 | 1,83078E-08 | -1,5918 | TargetScan Human | High (predicted) |
| 11754859_x_at | 80183 | C13orf18 | chromosome 13 open reading frame 18 | NM_025113 | 1,19897E-10 | -3,78458 | TargetScan Human | High (predicted) |
| 11718565_at | 57189 | KIAA1147 | KIAA1147 | NM_001080392 | 1,5382E-07 | -1,55242 | TargetScan Human | High (predicted) |
| 11727145_s_at | 8462 | KLF11 | Kruppel-like factor 11 | NM_001177716 NM_001177718 NM_003597 | 0,00966761 | -1,68579 | TargetScan Human | High (predicted) |
| 11725085_a_at | 55915 | LANCL2 | LanC lantibiotic synthetase component C-like 2 (bacterial) | NM_018697 | 3,61979E-09 | -1,60749 | TargetScan Human | High (predicted) |
| 11753275_a_at | 9741 | LAPTM4A | lysosomal protein transmembrane 4 alpha | NM_014713 | 0,000142937 | -1,71311 | TargetScan Human | High (predicted) |
| 11723907_a_at | 55132 | LARP1B | La ribonucleoprotein domain family, member 1B | NM_018078 NM_032239 NM_178043 | 3,02439E-10 | -1,96756 | TargetScan Human | Moderate (predicted) |
| 11728429_a_at | 84458 | LCOR | ligand dependent nuclear receptor corepressor | NM_001170765 NM_001170766 NM_032440 | 1,98765E-06 | -1,57045 | TargetScan Human | High (predicted) |
| 11731288_a_at | 8932 | MBD2 | methyl-CpG binding domain protein 2 | NM_003927 NM_015832 | 6,69654E-05 | -1,75775 | TargetScan Human | High (predicted) |
| 11731565_x_at | 4193 | MDM2 | Mdm2 p53 binding protein homolog (mouse) | NM_001145336 NM_001145337 NM_001145339 NM_001145340 NM_002392 NM_00 | 5,00917E-07 | -1,79256 | TargetScan Human | High (predicted) |
| 11722251_a_at | 4751 | NEK2 | NIMA (never in mitosis gene a)-related kinase 2 | NM_002497 | 0,0347364 | -1,78287 | TargetScan Human | Moderate (predicted) |
| 11738447_a_at | 10725 | NFAT5 | nuclear factor of activated T-cells 5, tonicity-responsive | NM_001113178 NM_006599 NM_138713 NM_138714 NM_173214 | 8,14917E-09 | -1,69362 | TargetScan Human | High (predicted) |
| 11763443_at | 7181 | NR2C1 | Nuclear receptor subfamily 2, group C, member 1 | NM_001032287 NM_001127362 NM_003297 | 3,20549E-07 | -1,87468 | TargetScan Human | Moderate (predicted) |
| 11760519_at | 64324 | NSD1 | nuclear receptor binding SET domain protein 1 | NM_022455 NM_172349 | 0,00204969 | -1,52268 | TargetScan Human | High (predicted) |
| 11724095_a_at | 5142 | PDE4B | phosphodiesterase 4B, cAMP-specific (phosphodiesterase E4 dunce homolog, Drosophila) | NM_001037339 NM_001037340 NM_001037341 NM_002600 | 2,96229E-11 | -5,23273 | TargetScan Human | High (predicted) |
| 11724953_a_at | 149420 | PDIK1L | PDLIM1 interacting kinase 1 like | NM_152835 NR_026685 NR_026686 | 3,31068E-07 | -1,54315 | TargetScan Human | Moderate (predicted) |
| 11725271_a_at | 5277 | PIGA | phosphatidylinositol glycan anchor biosynthesis, class A | NM_002641 NM_020473 | 2,82669E-06 | -1,99682 | TargetScan Human | Moderate (predicted) |
| 11735019_a_at | 5495 | PPM1B | protein phosphatase, Mg2+/Mn2+ dependent, 1B | NM_001033556 NM_001033557 NM_002706 NM_177968 NM_177969 | 4,49324E-05 | -1,75769 | TargetScan Human | High (predicted) |
| 11739370_a_at | 8493 | PPM1D | protein phosphatase, Mg2+/Mn2+ dependent, 1D | NM_003620 | 5,46636E-07 | -1,85351 | TargetScan Human | High (predicted) |
| 11743384_at | 55660 | PRPF40A | PRP40 pre-mRNA processing factor 40 homolog A (S. cerevisiae) | NM_017892 | 9,872E-11 | -1,82155 | TargetScan Human | High (predicted) |
| 11758421_s_at | 5728 | PTEN | phosphatase and tensin homolog | NM_000314 | 2,41997E-07 | -1,59627 | TargetScan Human,miRecords | Experimentally Observed,High (predicted) |
| 11743596_a_at | 5791 | PTPRE | protein tyrosine phosphatase, receptor type, E | NM_006504 NM_130435 | 4,252E-08 | -3,11763 | TargetScan Human | High (predicted) |
| 11716923_a_at | 5814 | PURB | purine-rich element binding protein B | NM_033224 | 8,21947E-06 | -2,53453 | TargetScan Human | High (predicted) |
| 11722789_a_at | 5868 | RAB5A | RAB5A, member RAS oncogene family | NM_004162 | 0,00136428 | -1,67924 | TargetScan Human | High (predicted) |
| 11731506_a_at | 5887 | RAD23B | RAD23 homolog B (S. cerevisiae) | NM_002874 | 8,23995E-08 | -1,88169 | TargetScan Human | High (predicted) |
| 11739558_at | 55159 | RFWD3 | ring finger and WD repeat domain 3 | NM_018124 | 1,62961E-07 | -1,73273 | TargetScan Human | Moderate (predicted) |
| 11752004_s_at | 285190 400966 653489 727851 729540 729857 84220 9648 | GCC2 RGPD1 RGPD2 RGPD3 RGPD4 RGPD5 RGPD6 RGPD8 | GRIP and coiled-coil domain containing 2 RANBP2-like and GRIP domain containing 1 / | NM_001024457 NM_001037866 NM_001078170 NM_001123363 NM_001144013 NM | 0,000247233 | -2,283 | TargetScan Human | Moderate (predicted) |
| 11733865_at | 10556 | RPP30 | ribonuclease P/MRP 30kDa subunit | NM_001104546 NM_006413 | 2,12449E-09 | -1,78682 | TargetScan Human | Moderate (predicted) |
| 11717357_at | 23168 | RTF1 | Rtf1, Paf1/RNA polymerase II complex component, homolog (S. cerevisiae) | NM_015138 | 6,27095E-11 | -1,77578 | TargetScan Human | High (predicted) |
| 11732841_a_at | 6328 | SCN3A | sodium channel, voltage-gated, type III, alpha subunit | NM_001081676 NM_001081677 NM_006922 | 2,05526E-09 | -2,74159 | miRecords | Experimentally Observed |
| 11717555_at | 80854 | SETD7 | SET domain containing (lysine methyltransferase) 7 | NM_030648 | 3,06701E-10 | -2,05752 | TargetScan Human | High (predicted) |
| 11724483_s_at | 8036 | SHOC2 | soc-2 suppressor of clear homolog (C. elegans) | NM_007373 | 4,91262E-10 | -1,51818 | TargetScan Human | Moderate (predicted) |
| 11761728_a_at | 6605 | SMARCE1 | SWI/SNF related, matrix associated, actin dependent regulator of chromatin, subfamily e | NM_003079 | 1,30833E-05 | -1,83638 | TargetScan Human | High (predicted) |
| 11762978_x_at | 6606 | SMN1 | survival of motor neuron 1, telomeric | NM_000344 NM_022874 | 3,63971E-07 | -1,69677 | TargetScan Human | Moderate (predicted) |
| 11758088_s_at | 9306 | SOCS6 | suppressor of cytokine signaling 6 | NM_004232 | 6,5438E-09 | -1,66535 | TargetScan Human | High (predicted) |
| 11721860_s_at | 23673 | STX12 | syntaxin 12 | NM_177424 | 1,24236E-07 | -2,21584 | TargetScan Human | High (predicted) |
| 11722797_a_at | 90488 | C12orf23 | chromosome 12 open reading frame 23 | NM_152261 | 5,36142E-12 | -2,02704 | TargetScan Human | High (predicted) |
| 11743501_a_at | 7187 | TRAF3 | TNF receptor-associated factor 3 | NM_003300 NM_145725 NM_145726 | 2,07491E-06 | -1,76473 | TargetScan Human | High (predicted) |
| 11728224_a_at | 51465 | UBE2J1 | ubiquitin-conjugating enzyme E2, J1 (UBC6 homolog, yeast) | NM_016021 | 2,93162E-10 | -2,62894 | TargetScan Human | Moderate (predicted) |
| 11755253_a_at | 205717 | KIAA2018 | KIAA2018 | NM_001009899 | 2,14624E-09 | -1,70501 | TargetScan Human | High (predicted) |
| 11763812_at | 7404 | UTY | ubiquitously transcribed tetratricopeptide repeat gene, Y-linked | NM_007125 NM_182659 NM_182660 | 0,00353017 | -1,83284 | TargetScan Human | Moderate (predicted) |
| 11734909_s_at | 80232 | WDR26 | WD repeat domain 26 | NM_001115113 NM_025160 | 8,06332E-07 | -1,83257 | TargetScan Human | High (predicted) |
| 11750162_a_at | 56270 | WDR45L | WDR45-like | NM_019613 | 0,000249383 | -1,5682 | TargetScan Human | High (predicted) |
| 11759628_at | 7456 | WIPF1 | WAS/WASL interacting protein family, member 1 | NM_001077269 NM_003387 | 9,86729E-06 | -1,99291 | TargetScan Human | High (predicted) |
| 11751494_a_at | 10138 | YAF2 | YY1 associated factor 2 | NM_005748 | 9,82846E-06 | -1,55971 | TargetScan Human | High (predicted) |
| 11725236_at | 9880 | ZBTB39 | zinc finger and BTB domain containing 39 | NM_014830 | 5,68596E-09 | -1,5765 | TargetScan Human | High (predicted) |
| 11722288_a_at | 23099 | ZBTB43 | zinc finger and BTB domain containing 43 | NM_001135776 NM_014007 | 2,17379E-06 | -2,26848 | TargetScan Human | High (predicted) |
| 11759647_x_at | 29066 | ZC3H7A | zinc finger CCCH-type containing 7A | NM_014153 | 2,12449E-09 | -1,56857 | TargetScan Human | High (predicted) |
| 11729733_a_at | 23528 | ZNF281 | zinc finger protein 281 | NM_012482 | 2,48119E-05 | -1,53169 | TargetScan Human | High (predicted) |
| 11759507_at | 23036 | ZNF292 | zinc finger protein 292 | NM_015021 | 6,42541E-08 | -1,66146 | TargetScan Human | High (predicted) |
| 11731962_a_at | 163051 | ZNF709 | zinc finger protein 709 | NM_001145647 NM_152601 | 0,00874238 | -1,84652 | TargetScan Human | Moderate (predicted) |
| 11749755_a_at | 163049 | ZNF791 | zinc finger protein 791 | NM_153358 | 2,53836E-05 | -2,15816 | TargetScan Human | High (predicted) |
| 11755138_s_at | 158586 | ZXDB | zinc finger, X-linked, duplicated B | NM_007157 | 1,61971E-05 | -1,56158 | TargetScan Human | Moderate (predicted) |

**Table 2 : PMF downregulated miRNA targets.** This table represents the list of PMF downregulated genes associated to at least one overexpressed miRNA according to integrative analysis.

| **Probeset ID** | **Entrez Gene** | **Gene Symbol** | **Gene Title** | **RefSeq Transcript ID** | **False Discovery Rate** | **Fold-Change** |
| --- | --- | --- | --- | --- | --- | --- |
| 11719079_at | 79719 | AAGAB | alpha- and gamma-adaptin binding protein | NM_024666 | 9,09417E-09 | -1,61722 |
| 11736635_a_at | 22848 | AAK1 | AP2 associated kinase 1 | NM_014911 | 0,000102223 | -1,59649 |
| 11739503_at | 19 | ABCA1 | ATP-binding cassette, sub-family A (ABC1), member 1 | NM_005502 | 0,00799727 | -1,6398 |
| 11741592_x_at | 5825 | ABCD3 | ATP-binding cassette, sub-family D (ALD), member 3 | NM_001122674 NM_002858 | 3,40977E-10 | -2,42991 |
| 11730849_at | 84945 | ABHD13 | abhydrolase domain containing 13 | NM_032859 | 6,2521E-12 | -2,08247 |
| 11733048_a_at | 10006 | ABI1 | abl-interactor 1 | NM_001012750 NM_001012751 NM_001012752 NM_001178116 NM_001178119 NM | 7,29415E-06 | -1,81965 |
| 11743566_at | 64746 | ACBD3 | acyl-CoA binding domain containing 3 | NM_022735 | 0,000096383 | -1,50422 |
| 11743996_a_at | 10121 | ACTR1A | ARP1 actin-related protein 1 homolog A, centractin alpha (yeast) | NM_005736 | 7,86133E-07 | -1,53367 |
| 11755796_a_at | 8754 | ADAM9 | ADAM metallopeptidase domain 9 (meltrin gamma) | NM_001005845 NM_003816 NR_027638 NR_027639 NR_027878 | 5,04449E-06 | -2,08246 |
| 11719515_at | 22850 | ADNP2 | ADNP homeobox 2 | NM_014913 | 1,87943E-07 | -1,92046 |
| 11716022_at | 156 | ADRBK1 | adrenergic, beta, receptor kinase 1 | NM_001619 | 1,05759E-08 | -2,54831 |
| 11739696_s_at | 3899 | AFF3 | AF4/FMR2 family, member 3 | NM_001025108 NM_002285 | 0,000632985 | -2,17145 |
| 11720422_s_at | 27125 | AFF4 | AF4/FMR2 family, member 4 | NM_014423 | 1,88921E-08 | -1,8391 |
| 11740338_a_at | 3267 | AGFG1 | ArfGAP with FG repeats 1 | NM_001135187 NM_001135188 NM_001135189 NM_004504 | 9,14734E-09 | -2,22516 |
| 11732450_s_at | 375790 | AGRN | agrin | NM_198576 | 0,0038717 | -1,63237 |
| 11730253_a_at | 25909 | AHCTF1 | AT hook containing transcription factor 1 | NM_015446 | 4,23996E-09 | -1,74133 |
| 11719247_at | 202 | AIM1 | absent in melanoma 1 | NM_001624 | 0,0440138 | -1,70698 |
| 11723804_a_at | 9590 | AKAP12 | A kinase (PRKA) anchor protein 12 | NM_005100 NM_144497 | 1,20426E-05 | -5,75698 |
| 11730741_a_at | 79647 | AKIRIN1 | akirin 1 | NM_001136275 NM_024595 | 0,00174444 | -1,80896 |
| 11721753_a_at | 79868 | ALG13 | asparagine-linked glycosylation 13 homolog (S. cerevisiae) | NM_001039210 NM_001099922 NM_001168385 NM_018466 NR_033124 NR_03312 | 5,00753E-05 | -1,67228 |
| 11757951_s_at | 85365 | ALG2 | asparagine-linked glycosylation 2, alpha-1,3-mannosyltransferase homolog (S. cerevisiae | NM_033087 NR_024532 | 5,26929E-07 | -1,53384 |
| 11757688_a_at | 54882 | ANKHD1 | ankyrin repeat and KH domain containing 1 | NM_017747 NM_017978 NM_024668 | 6,2737E-06 | -2,15237 |
| 11761390_a_at | 55608 | ANKRD10 | ankyrin repeat domain 10 | NM_017664 | 3,11813E-08 | -1,52487 |
| 11759780_at | 81573 | ANKRD13C | ankyrin repeat domain 13C | NM_030816 | 3,07627E-07 | -2,41324 |
| 11750847_a_at | 91526 | ANKRD44 | ankyrin repeat domain 44 | NM_153697 | 8,10324E-07 | -1,92417 |
| 11757852_s_at | 164 | AP1G1 | adaptor-related protein complex 1, gamma 1 subunit | NM_001030007 NM_001128 | 0,000200768 | -1,6986 |
| 11761034_at | 54518 | APBB1IP | amyloid beta (A4) precursor protein-binding, family B, member 1 interacting protein | NM_019043 | 2,04646E-06 | -1,61711 |
| 11741085_a_at | 334 | APLP2 | amyloid beta (A4) precursor-like protein 2 | NM_001142276 NM_001142277 NM_001142278 NM_001642 NR_024515 NR_02451 | 0,000365511 | -1,5435 |
| 11715306_s_at | 374 | AREG | amphiregulin | NM_001657 | 0,000400484 | -4,41772 |
| 11715428_x_at | 378 | ARF4 | ADP-ribosylation factor 4 | NM_001660 | 9,19137E-05 | -1,51323 |
| 11717401_at | 382 | ARF6 | ADP-ribosylation factor 6 | NM_001663 | 4,70426E-08 | -1,62277 |
| 11721063_a_at | 10564 | ARFGEF2 | ADP-ribosylation factor guanine nucleotide-exchange factor 2 (brefeldin A-inhibited) | NM_006420 | 9,44733E-06 | -1,70771 |
| 11719347_a_at | 84986 | ARHGAP19 | Rho GTPase activating protein 19 | NM_032900 | 0,00352245 | -1,5392 |
| 11726074_a_at | 8874 | ARHGEF7 | Rho guanine nucleotide exchange factor (GEF) 7 | NM_001113511 NM_001113512 NM_001113513 NM_003899 NM_145735 | 3,23967E-11 | -2,20111 |
| 11734882_a_at | 196528 | ARID2 | AT rich interactive domain 2 (ARID, RFX-like) | NM_152641 | 1,19994E-11 | -1,78271 |
| 11743712_a_at | 5926 | ARID4A | AT rich interactive domain 4A (RBP1-like) | NM_002892 NM_023000 NM_023001 | 1,42432E-07 | -2,27168 |
| 11721879_s_at | 51742 | ARID4B | AT rich interactive domain 4B (RBP1-like) | NM_016374 NM_031371 | 1,08791E-06 | -1,68441 |
| 11734935_a_at | 10865 | ARID5A | AT rich interactive domain 5A (MRF1-like) | NM_212481 | 5,05781E-09 | -2,12102 |
| 11717698_a_at | 25820 | ARIH1 | ariadne homolog, ubiquitin-conjugating enzyme E2 binding protein, 1 (Drosophila) | NM_005744 | 1,21318E-09 | -2,15254 |
| 11737104_at | 51326 | ARL17A | ADP-ribosylation factor-like 17A | NM_001113738 NM_016632 | 0,00965707 | -1,50781 |
| 11732331_s_at | 10123 | ARL4C | ADP-ribosylation factor-like 4C | NM_005737 | 0,038891 | -1,61516 |
| 11729916_s_at | 221079 | ARL5B | ADP-ribosylation factor-like 5B | NM_178815 | 0,000194799 | -1,93321 |
| 11720466_a_at | 23204 | ARL6IP1 | ADP-ribosylation factor-like 6 interacting protein 1 | NM_015161 | 0,00856816 | -1,53475 |
| 11733929_a_at | 25852 | ARMC8 | armadillo repeat containing 8 | NM_014154 NM_015396 NM_213654 | 8,12902E-08 | -2,2715 |
| 11758853_a_at | 81873 | ARPC5L | actin related protein 2/3 complex, subunit 5-like | NM_030978 | 1,82065E-07 | -2,26001 |
| 11742557_s_at | 10776 | ARPP19 | cAMP-regulated phosphoprotein, 19kDa | NM_006628 | 1,42594E-05 | -1,64984 |
| 11728365_a_at | 10777 | ARPP21 | cyclic AMP-regulated phosphoprotein, 21 kD | NM_001025068 NM_001025069 NM_016300 NM_198399 | 5,35418E-06 | -3,10875 |
| 11759778_at | 171023 | ASXL1 | additional sex combs like 1 (Drosophila) | NM_001164603 NM_015338 | 3,90225E-06 | -2,46761 |
| 11756885_a_at | 29028 | ATAD2 | ATPase family, AAA domain containing 2 | NM_014109 | 1,66381E-09 | -1,96823 |
| 11722058_s_at | 64225 | ATL2 | atlastin GTPase 2 | NM_001135673 NM_022374 NR_024191 | 2,06889E-07 | -1,72424 |
| 11755872_a_at | 23200 | ATP11B | ATPase, class VI, type 11B | NM_014616 | 4,27531E-06 | -1,87338 |
| 11754302_a_at | 488 | ATP2A2 | ATPase, Ca++ transporting, cardiac muscle, slow twitch 2 | NM_001135765 NM_001681 NM_170665 | 5,0458E-09 | -1,5376 |
| 11745223_a_at | 27032 | ATP2C1 | ATPase, Ca++ transporting, type 2C, member 1 | NM_001001485 NM_001001486 NM_001001487 NM_014382 | 2,4125E-06 | -1,75364 |
| 11743886_at | 342371 | ATXN1L | ataxin 1-like | NM_001137675 NR_024612 | 2,50621E-11 | -2,1775 |
| 11738263_s_at | 26053 | AUTS2 | autism susceptibility candidate 2 | NM_001127231 NM_001127232 NM_015570 | 1,35495E-06 | -1,85482 |
| 11716155_s_at | 2683 | B4GALT1 | UDP-Gal:betaGlcNAc beta 1,4- galactosyltransferase, polypeptide 1 | NM_001497 | 0,000119149 | -1,54228 |
| 11726369_at | 571 | BACH1 | BTB and CNC homology 1, basic leucine zipper transcription factor 1 | NM_001011545 NM_001186 NM_206866 NR_027655 | 0,00223504 | -1,55803 |
| 11741378_s_at | 60468 | BACH2 | BTB and CNC homology 1, basic leucine zipper transcription factor 2 | NM_001170794 NM_021813 | 0,00999882 | -1,64785 |
| 11749886_a_at | 23786 | BCL2L13 | BCL2-like 13 (apoptosis facilitator) | NM_015367 | 3,77317E-08 | -1,58722 |
| 11717171_at | 599 | BCL2L2 | BCL2-like 2 | NM_004050 | 2,04098E-06 | -1,68477 |
| 11725445_x_at | 602 | BCL3 | B-cell CLL/lymphoma 3 | NM_005178 | 7,44583E-05 | -2,03026 |
| 11720746_s_at | 604 | BCL6 | B-cell CLL/lymphoma 6 | NM_001130845 NM_001134738 NM_001706 | 0,00188706 | -2,21713 |
| 11719907_a_at | 605 | BCL7A | B-cell CLL/lymphoma 7A | NM_001024808 NM_020993 | 8,94749E-10 | -1,52785 |
| 11728217_a_at | 9774 | BCLAF1 | BCL2-associated transcription factor 1 | NM_001077440 NM_001077441 NM_014739 | 1,87188E-09 | -1,98585 |
| 11724716_a_at | 54880 | BCOR | BCL6 co-repressor | NM_001123383 NM_001123384 NM_001123385 NM_017745 | 0,000614151 | -1,70472 |
| 11727346_a_at | 640 | BLK | B lymphoid tyrosine kinase | NM_001715 | 6,08518E-07 | -1,60061 |
| 11737440_a_at | 90427 | BMF | Bcl2 modifying factor | NM_001003940 NM_001003942 NM_001003943 NM_033503 | 0,000772602 | -1,51723 |
| 11741783_a_at | 662 | BNIP1 | BCL2/adenovirus E1B 19kDa interacting protein 1 | NM_001205 NM_013978 NM_013979 NM_013980 | 1,34551E-09 | -1,71854 |
| 11723670_a_at | 663 | BNIP2 | BCL2/adenovirus E1B 19kDa interacting protein 2 | NM_004330 | 5,31152E-09 | -1,76227 |
| 11751151_a_at | 8315 | BRAP | BRCA1 associated protein | NM_006768 | 5,32574E-08 | -1,72763 |
| 11740416_at | 675 | BRCA2 | breast cancer 2, early onset | NM_000059 | 0,000914847 | -1,67936 |
| 11722643_a_at | 7862 | BRPF1 | bromodomain and PHD finger containing, 1 | NM_001003694 NM_004634 | 4,23851E-12 | -1,57127 |
| 11743550_at | 54014 | BRWD1 | bromodomain and WD repeat domain containing 1 | NM_001007246 NM_018963 NM_033656 | 9,03187E-08 | -1,82289 |
| 11733263_a_at | 55108 | BSDC1 | BSD domain containing 1 | NM_001143888 NM_001143889 NM_001143890 NM_018045 | 3,08761E-07 | -1,95591 |
| 11722990_s_at | 9044 | BTAF1 | BTAF1 RNA polymerase II, B-TFIID transcription factor-associated, 170kDa (Mot1 homolog, | NM_003972 | 1,13711E-11 | -1,55922 |
| 11733022_at | 694 | BTG1 | B-cell translocation gene 1, anti-proliferative | NM_001731 | 4,6548E-07 | -3,77192 |
| 11729163_a_at | 10950 | BTG3 | BTG family, member 3 | NM_001130914 NM_006806 | 1,84372E-05 | -2,30556 |
| 11755370_x_at | 9256 | BZRAP1 | benzodiazapine receptor (peripheral) associated protein 1 | NM_004758 NM_024418 | 0,000109738 | -2,12596 |
| 11744775_x_at | 151579 9689 | BZW1 BZW1L1 | basic leucine zipper and W2 domains 1 basic leucine zipper and W2 domains 1 like 1 | NM_014670 NR_026584 | 2,59274E-07 | -2,21057 |
| 11731624_a_at | 55088 | C10orf118 | chromosome 10 open reading frame 118 | NM_018017 | 0,00682453 | -1,55408 |
| 11760585_at | 56946 | C11orf30 | chromosome 11 open reading frame 30 | NM_020193 | 2,58844E-08 | -1,85381 |
| 11723639_s_at | 10944 | C11orf58 | chromosome 11 open reading frame 58 | NM_001142705 NM_014267 | 4,86982E-09 | -1,79918 |
| 11722797_a_at | 90488 | C12orf23 | chromosome 12 open reading frame 23 | NM_152261 | 5,36142E-12 | -2,02704 |
| 11717582_a_at | 28984 | C13orf15 | chromosome 13 open reading frame 15 | NM_014059 | 0,0277971 | -1,72804 |
| 11754859_x_at | 80183 | C13orf18 | chromosome 13 open reading frame 18 | NM_025113 | 1,19897E-10 | -3,78458 |
| 11731004_at | 55320 | C14orf106 | chromosome 14 open reading frame 106 | NM_018353 | 4,8348E-09 | -1,65618 |
| 11721232_s_at | 79609 | C14orf138 | chromosome 14 open reading frame 138 | NM_001040662 NM_024558 | 8,83119E-09 | -1,69061 |
| 11722752_a_at | 91748 | C14orf43 | chromosome 14 open reading frame 43 | NM_001043318 NM_194278 | 6,68281E-05 | -1,84632 |
| 11743350_a_at | 84419 | C15orf48 | chromosome 15 open reading frame 48 | NM_032413 NM_197955 | 0,020834 | -2,09252 |
| 11736227_a_at | 388272 | C16orf87 | chromosome 16 open reading frame 87 | NM_001001436 | 0,000515732 | -1,74772 |
| 11746025_a_at | 284021 | C17orf60 | chromosome 17 open reading frame 60 | NM_001085423 | 9,37586E-06 | -1,72368 |
| 11724672_s_at | 23587 | C17orf81 | chromosome 17 open reading frame 81 | NM_015362 NM_203413 NM_203414 NM_203415 | 3,72111E-13 | -1,67785 |
| 11748073_a_at | 753 | C18orf1 | chromosome 18 open reading frame 1 | NM_001003674 NM_001003675 NM_004338 NM_181481 NM_181482 NM_181483 | 0,00138476 | -1,72319 |
| 11756446_a_at | 57095 | C1orf128 | chromosome 1 open reading frame 128 | NM_020362 | 9,28208E-06 | -1,88299 |
| 11752610_a_at | 9473 | C1orf38 | chromosome 1 open reading frame 38 | NM_001039477 NM_001105556 NM_004848 | 0,00289233 | -1,66704 |
| 11735042_a_at | 148423 | C1orf52 | chromosome 1 open reading frame 52 | NM_198077 NR_024113 | 8,219E-10 | -1,52716 |
| 11729298_a_at | 163859 | C1orf55 | chromosome 1 open reading frame 55 | NM_152608 | 7,05491E-09 | -2,14989 |
| 11743322_at | 51526 | C20orf111 | chromosome 20 open reading frame 111 | NM_016470 | 4,92749E-09 | -2,69228 |
| 11754489_a_at | 79133 | C20orf7 | chromosome 20 open reading frame 7 | NM_001039375 NM_024120 NR_029377 | 1,33313E-10 | -1,92723 |
| 11729697_at | 205327 | C2orf69 | chromosome 2 open reading frame 69 | NM_153689 | 8,21611E-07 | -1,50781 |
| 11726038_s_at | 285237 | C3orf38 | chromosome 3 open reading frame 38 | NM_173824 | 0,0021607 | -1,52308 |
| 11755179_x_at | 375341 | C3orf62 | chromosome 3 open reading frame 62 | NM_198562 | 4,49417E-07 | -1,63559 |
| 11763368_a_at | 55345 | C4orf21 | chromosome 4 open reading frame 21 | NM_001099776 NM_018392 | 0,00137944 | -1,71425 |
| 11744932_a_at | 153222 | C5orf41 | chromosome 5 open reading frame 41 | NM_001168393 NM_001168394 NM_153607 | 0,00781532 | -2,01702 |
| 11717937_at | 85027 | C5orf62 | chromosome 5 open reading frame 62 | NM_032947 | 1,00514E-06 | -1,72353 |
| 11717122_s_at | 64771 | C6orf106 | chromosome 6 open reading frame 106 | NM_022758 NM_024294 | 8,39614E-06 | -1,68612 |
| 11738867_a_at | 85411 | C6orf114 | chromosome 6 open reading frame 114 | NM_033069 | 0,000259082 | -2,14254 |
| 11740806_a_at | 387119 | C6orf204 | chromosome 6 open reading frame 204 | NM_001042475 NM_001178035 NM_206921 | 0,00348317 | -1,52017 |
| 11723458_x_at | 84060 | C7orf64 | chromosome 7 open reading frame 64 | NM_032120 | 5,08628E-06 | -1,52227 |
| 11736070_at | 65265 | C8orf33 | chromosome 8 open reading frame 33 | NM_023080 | 1,22929E-05 | -1,57635 |
| 11751867_a_at | 401466 | C8orf59 | chromosome 8 open reading frame 59 | NM_001099670 NM_001099671 NM_001099672 NM_001099673 | 3,20635E-05 | -2,18307 |
| 11737176_at | 203228 | C9orf72 | chromosome 9 open reading frame 72 | NM_018325 NM_145005 | 0,00440911 | -1,66081 |
| 11763465_at | 91768 | CABLES1 | Cdk5 and Abl enzyme substrate 1 | NM_001100619 NM_138375 NR_023359 | 0,000122105 | -1,61531 |
| 11723567_s_at | 23473 | CAPN7 | calpain 7 | NM_014296 | 2,83303E-07 | -1,50322 |
| 11734717_a_at | 79872 | CBLL1 | Cas-Br-M (murine) ecotropic retroviral transforming sequence-like 1 | NM_024814 NR_024199 | 1,06006E-07 | -1,62211 |
| 11719987_a_at | 150275 | CCDC117 | coiled-coil domain containing 117 | NM_173510 | 9,52952E-08 | -1,56434 |
| 11733359_a_at | 25790 | CCDC19 | coiled-coil domain containing 19 | NM_012337 | 8,82448E-05 | -1,73022 |
| 11743728_a_at | 57003 | CCDC47 | coiled-coil domain containing 47 | NM_020198 | 9,95391E-08 | -1,79082 |
| 11733157_a_at | 152137 | CCDC50 | coiled-coil domain containing 50 | NM_174908 NM_178335 | 3,11637E-05 | -1,8007 |
| 11755625_a_at | 60494 | CCDC81 | coiled-coil domain containing 81 | NM_001156474 NM_021827 | 0,000248095 | -1,6768 |
| 11739604_a_at | 55704 | CCDC88A | coiled-coil domain containing 88A | NM_001135597 NM_018084 | 1,07678E-09 | -1,82487 |
| 11726624_a_at | 901 | CCNG2 | cyclin G2 | NM_004354 | 0,000066986 | -1,56228 |
| 11724034_at | 23607 | CD2AP | CD2-associated protein | NM_012120 | 1,32306E-06 | -1,56359 |
| 11742219_a_at | 4179 | CD46 | CD46 molecule, complement regulatory protein | NM_002389 NM_153826 NM_172350 NM_172351 NM_172352 NM_172353 NM_ | 0,00285675 | -1,70738 |
| 11741089_x_at | 973 | CD79A | CD79a molecule, immunoglobulin-associated alpha | NM_001783 NM_021601 | 8,77475E-10 | -1,79923 |
| 11745466_a_at | 81602 | CDADC1 | cytidine and dCMP deaminase domain containing 1 | NM_030911 | 0,00227268 | -1,58541 |
| 11725576_s_at | 8556 | CDC14A | CDC14 cell division cycle 14 homolog A (S. cerevisiae) | NM_003672 NM_033312 NM_033313 | 0,000159203 | -1,58918 |
| 11752920_a_at | 996 | CDC27 | cell division cycle 27 homolog (S. cerevisiae) | NM_001114091 NM_001256 | 8,36963E-09 | -1,63547 |
| 11722605_at | 55664 | CDC37L1 | cell division cycle 37 homolog (S. cerevisiae)-like 1 | NM_017913 | 1,74901E-07 | -1,5631 |
| 11725717_at | 998 | CDC42 | cell division cycle 42 (GTP binding protein, 25kDa) | NM_001039802 NM_001791 NM_044472 | 7,91434E-07 | -4,90403 |
| 11743759_a_at | 56990 | CDC42SE2 | CDC42 small effector 2 | NM_001038702 NM_020240 | 3,36491E-05 | -1,79912 |
| 11726614_at | 1000 | CDH2 | cadherin 2, type 1, N-cadherin (neuronal) | NM_001792 | 0,00987811 | -1,58249 |
| 11739638_s_at | 51755 | CDK12 | cyclin-dependent kinase 12 | NM_015083 NM_016507 | 1,00415E-06 | -1,51263 |
| 11762335_a_at | 91368 | CDKN2AIPNL | CDKN2A interacting protein N-terminal like | NM_080656 | 5,47199E-05 | -1,65014 |
| 11721284_a_at | 8760 | CDS2 | CDP-diacylglycerol synthase (phosphatidate cytidylyltransferase) 2 | NM_003818 | 3,24855E-10 | -1,7878 |
| 11743135_s_at | 1052 | CEBPD | CCAAT/enhancer binding protein (C/EBP), delta | NM_005195 | 0,0200665 | -2,19942 |
| 11717694_a_at | 1054 | CEBPG | CCAAT/enhancer binding protein (C/EBP), gamma | NM_001806 | 9,87848E-10 | -2,46382 |
| 11725788_a_at | 55839 | CENPN | centromere protein N | NM_001100624 NM_001100625 NM_018455 | 0,000451716 | -1,5877 |
| 11752592_a_at | 9859 | CEP170 | centrosomal protein 170kDa | NM_001042404 NM_001042405 NM_014812 | 2,30576E-07 | -2,21018 |
| 11719055_a_at | 9857 | CEP350 | centrosomal protein 350kDa | NM_014810 | 2,17265E-08 | -1,65214 |
| 11720247_a_at | 55165 | CEP55 | centrosomal protein 55kDa | NM_001127182 NM_018131 | 0,0301578 | -1,91962 |
| 11759670_a_at | 9702 | CEP57 | centrosomal protein 57kDa | NM_014679 | 3,86486E-10 | -1,95783 |
| 11730777_a_at | 23177 | CEP68 | centrosomal protein 68kDa | NM_015147 | 0,00308288 | -1,52461 |
| 11754992_a_at | 1105 | CHD1 | chromodomain helicase DNA binding protein 1 | NM_001270 | 1,33276E-09 | -2,218 |
| 11743284_at | 1106 | CHD2 | chromodomain helicase DNA binding protein 2 | NM_001042572 NM_001271 | 4,88471E-12 | -1,89085 |
| 11736286_a_at | 1119 | CHKA | choline kinase alpha | NM_001277 NM_212469 | 7,39143E-05 | -1,65813 |
| 11724747_a_at | 25978 | CHMP2B | chromatin modifying protein 2B | NM_014043 | 4,39808E-05 | -1,6111 |
| 11716496_s_at | 128866 | CHMP4B | chromatin modifying protein 4B | NM_176812 | 2,76683E-05 | -1,62798 |
| 11743434_a_at | 50515 | CHST11 | carbohydrate (chondroitin 4) sulfotransferase 11 | NM_001173982 NM_018413 | 6,14015E-08 | -1,8383 |
| 11744300_at | 51363 | CHST15 | carbohydrate (N-acetylgalactosamine 4-sulfate 6-O) sulfotransferase 15 | NM_015892 | 0,000591386 | -1,581 |
| 11760514_x_at | 26586 | CKAP2 | cytoskeleton associated protein 2 | NM_001098525 NM_018204 | 5,31501E-07 | -2,65357 |
| 11744641_a_at | 56650 | CLDND1 | claudin domain containing 1 | NM_001040181 NM_001040182 NM_001040183 NM_001040199 NM_001040200 NM | 2,07297E-07 | -2,3711 |
| 11754405_s_at | 25932 | CLIC4 | chloride intracellular channel 4 | NM_013943 | 0,000373356 | -1,53009 |
| 11723955_a_at | 9685 | CLINT1 | clathrin interactor 1 | NM_014666 | 0,00131324 | -1,56789 |
| 11727415_at | 2055 | CLN8 | ceroid-lipofuscinosis, neuronal 8 (epilepsy, progressive with mental retardation) | NM_018941 | 1,20245E-05 | -1,75519 |
| 11746341_a_at | 1213 | CLTC | clathrin, heavy chain (Hc) | NM_004859 | 2,39247E-14 | -1,77879 |
| 11754038_a_at | 7555 | CNBP | CCHC-type zinc finger, nucleic acid binding protein | NM_001127192 NM_001127193 NM_001127194 NM_001127195 NM_001127196 NM | 0,00798281 | -1,53682 |
| 11759150_at | 4850 | CNOT4 | CCR4-NOT transcription complex, subunit 4 | NM_001008225 NM_013316 | 8,04976E-07 | -1,63675 |
| 11746336_a_at | 9337 | CNOT8 | CCR4-NOT transcription complex, subunit 8 | NM_004779 | 1,50271E-06 | -2,08241 |
| 11717275_s_at | 1289 | COL5A1 | collagen, type V, alpha 1 | NM_000093 | 9,2026E-06 | -2,07595 |
| 11724193_s_at | 9318 | COPS2 | COP9 constitutive photomorphogenic homolog subunit 2 (Arabidopsis) | NM_001143887 NM_004236 | 3,58984E-05 | -1,72765 |
| 11724259_at | 80219 | COQ10B | coenzyme Q10 homolog B (S. cerevisiae) | NM_025147 | 0,000231602 | -2,02215 |
| 11723281_s_at | 58487 | CREBZF | CREB/ATF bZIP transcription factor | NM_001039618 NR_028024 NR_028025 NR_028026 NR_028027 | 6,56972E-08 | -1,77945 |
| 11763851_a_at | 1393 | CRHBP | corticotropin releasing hormone binding protein | NM_001882 | 0,0179013 | -1,57988 |
| 11732701_a_at | 55454 | CSGALNACT2 | chondroitin sulfate N-acetylgalactosaminyltransferase 2 | NM_018590 | 1,03357E-07 | -1,93379 |
| 11763319_at | 1452 | CSNK1A1 | casein kinase 1, alpha 1 | NM_001025105 NM_001892 | 1,20098E-05 | -1,83911 |
| 11739917_a_at | 1453 | CSNK1D | casein kinase 1, delta | NM_001893 NM_139062 | 3,0084E-06 | -1,94158 |
| 11757721_s_at | 64651 | CSRNP1 | cysteine-serine-rich nuclear protein 1 | NM_033027 | 0,000157806 | -1,70339 |
| 11719480_a_at | 1475 | CSTA | cystatin A (stefin A) | NM_005213 | 0,0490103 | -2,13318 |
| 11746937_a_at | 10106 | CTDSP2 | CTD (carboxy-terminal domain, RNA polymerase II, polypeptide A) small phosphatase 2 | NM_005730 | 0,000538946 | -1,66292 |
| 11715442_s_at | 1490 | CTGF | connective tissue growth factor | NM_001901 | 0,000032919 | -3,573 |
| 11716505_a_at | 8452 | CUL3 | cullin 3 | NM_003590 | 5,81402E-12 | -1,56486 |
| 11759512_x_at | 54883 | CWC25 | CWC25 spliceosome-associated protein homolog (S. cerevisiae) | NM_017748 | 7,15436E-08 | -1,97924 |
| 11748974_s_at | 55280 | CWF19L1 | CWF19-like 1, cell cycle control (S. pombe) | NM_018294 | 1,16949E-12 | -1,63825 |
| 11758000_s_at | 1525 | CXADR | coxsackie virus and adenovirus receptor | NM_001338 | 1,44075E-08 | -1,93939 |
| 11720819_s_at | 6387 | CXCL12 | chemokine (C-X-C motif) ligand 12 | NM_000609 NM_001033886 NM_001178134 NM_199168 | 0,0300657 | -1,57477 |
| 11739094_a_at | 7852 | CXCR4 | chemokine (C-X-C motif) receptor 4 | NM_001008540 NM_003467 | 2,30717E-06 | -2,44243 |
| 11758591_s_at | 55086 | CXorf57 | chromosome X open reading frame 57 | NM_001184782 NM_018015 | 0,000453166 | -1,57377 |
| 11756266_a_at | 26999 | CYFIP2 | cytoplasmic FMR1 interacting protein 2 | NM_001037332 NM_001037333 NM_014376 | 4,4313E-06 | -2,34575 |
| 11723095_a_at | 1540 | CYLD | cylindromatosis (turban tumor syndrome) | NM_001042355 NM_001042412 NM_015247 | 0,000205575 | -1,78552 |
| 11750061_x_at | 9802 | DAZAP2 | DAZ associated protein 2 | NM_001136264 NM_001136266 NM_001136267 NM_001136268 NM_001136269 NM | 0,000473457 | -1,51633 |
| 11734713_s_at | 10926 | DBF4 | DBF4 homolog (S. cerevisiae) | NM_006716 | 3,83217E-11 | -1,84036 |
| 11719471_at | 55802 | DCP1A | DCP1 decapping enzyme homolog A (S. cerevisiae) | NM_018403 | 5,41483E-09 | -2,60276 |
| 11734569_a_at | 51164 | DCTN4 | dynactin 4 (p62) | NM_001135643 NM_001135644 NM_016221 | 0,000227406 | -1,58263 |
| 11743972_a_at | 54541 | DDIT4 | DNA-damage-inducible transcript 4 | NM_019058 | 0,0166065 | -1,71583 |
| 11763277_at | 11269 | DDX19B | DEAD (Asp-Glu-Ala-As) box polypeptide 19B | NM_001014449 NM_001014451 NM_007242 | 1,39131E-07 | -1,73516 |
| 11733073_a_at | 9188 | DDX21 | DEAD (Asp-Glu-Ala-Asp) box polypeptide 21 | NM_004728 | 1,03709E-09 | -1,53023 |
| 11734665_x_at | 8653 | DDX3Y | DEAD (Asp-Glu-Ala-Asp) box polypeptide 3, Y-linked | NM_001122665 NM_004660 | 0,00906933 | -1,74617 |
| 11718968_a_at | 1656 | DDX6 | DEAD (Asp-Glu-Ala-Asp) box polypeptide 6 | NM_004397 | 0,000120849 | -1,94136 |
| 11759340_at | 163486 | DENND1B | DENN/MADD domain containing 1B | NM_001142795 NM_144977 | 9,61799E-09 | -1,58389 |
| 11758317_s_at | 22898 | DENND3 | DENN/MADD domain containing 3 | NM_014957 | 3,80205E-05 | -1,59722 |
| 11736710_a_at | 10260 | DENND4A | DENN/MADD domain containing 4A | NM_001144823 NM_005848 | 0,000470729 | -1,62187 |
| 11742766_a_at | 23258 | DENND5A | DENN/MADD domain containing 5A | NM_015213 | 3,46021E-05 | -1,543 |
| 11718109_s_at | 1738 | DLD | dihydrolipoamide dehydrogenase | NM_000108 | 1,84372E-05 | -1,52736 |
| 11741748_a_at | 1739 | DLG1 | discs, large homolog 1 (Drosophila) | NM_001098424 NM_004087 | 1,00838E-06 | -1,61129 |
| 11720932_s_at | 1743 | DLST | dihydrolipoamide S-succinyltransferase (E2 component of 2-oxo-glutarate complex) | NM_001933 | 1,78297E-09 | -1,53491 |
| 11724711_a_at | 1657 | DMXL1 | Dmx-like 1 | NM_005509 | 1,89443E-05 | -2,30245 |
| 11753512_s_at | 10049 65084 | DNAJB6 TMEM135 | DnaJ (Hsp40) homolog, subfamily B, member 6 transmembrane protein 135 | NM_001168724 NM_005494 NM_022918 NM_058246 NR_033149 | 3,54295E-07 | -1,86481 |
| 11727646_a_at | 548645 | DNAJC25 | DnaJ (Hsp40) homolog, subfamily C , member 25 | NM_001015882 | 1,83267E-08 | -1,68898 |
| 11763209_at | 85440 | DOCK7 | dedicator of cytokinesis 7 | NM_033407 | 0,00234248 | -1,5385 |
| 11721398_a_at | 84444 | DOT1L | DOT1-like, histone H3 methyltransferase (S. cerevisiae) | NM_032482 | 1,65081E-08 | -2,53427 |
| 11743286_at | 23220 | DTX4 | deltex homolog 4 (Drosophila) | NM_015177 | 5,42814E-10 | -1,7555 |
| 11758143_s_at | 1850 | DUSP8 | dual specificity phosphatase 8 | NM_004420 | 9,98887E-05 | -2,37479 |
| 11735012_a_at | 140735 | DYNLL2 | dynein, light chain, LC8-type 2 | NM_080677 | 3,35094E-06 | -2,29427 |
| 11724612_a_at | 8445 | DYRK2 | dual-specificity tyrosine-(Y)-phosphorylation regulated kinase 2 | NM_003583 NM_006482 | 1,08514E-07 | -1,71319 |
| 11724079_s_at | 1870 | E2F2 | E2F transcription factor 2 | NM_004091 | 0,000213696 | -1,73986 |
| 11730029_a_at | 144455 | E2F7 | E2F transcription factor 7 | NM_203394 | 0,00120786 | -1,84061 |
| 11728957_a_at | 79733 | E2F8 | E2F transcription factor 8 | NM_024680 | 0,0416746 | -1,55403 |
| 11758126_s_at | 85403 | EAF1 | ELL associated factor 1 | NM_033083 | 2,10914E-05 | -1,66706 |
| 11726801_at | 1879 | EBF1 | early B-cell factor 1 | NM_024007 | 6,75663E-06 | -6,7708 |
| 11722262_at | 9695 | EDEM1 | ER degradation enhancer, mannosidase alpha-like 1 | NM_014674 | 1,52822E-13 | -1,84331 |
| 11733856_s_at | 54583 | EGLN1 | egl nine homolog 1 (C. elegans) | NM_022051 | 0,0410296 | -1,55229 |
| 11722151_a_at | 9451 | EIF2AK3 | eukaryotic translation initiation factor 2-alpha kinase 3 | NM_004836 | 5,73177E-17 | -2,9709 |
| 11725637_a_at | 8669 | EIF3J | eukaryotic translation initiation factor 3, subunit J | NM_003758 | 3,98784E-08 | -1,5931 |
| 11760707_a_at | 1974 | EIF4A2 | eukaryotic translation initiation factor 4A2 | NM_001967 | 4,51994E-05 | -1,60933 |
| 11744930_a_at | 8672 | EIF4G3 | eukaryotic translation initiation factor 4 gamma, 3 | NM_003760 | 0,00309996 | -1,80528 |
| 11750029_a_at | 7458 | EIF4H | eukaryotic translation initiation factor 4H | NM_022170 NM_031992 | 1,3414E-06 | -2,05789 |
| 11724727_a_at | 1983 | EIF5 | eukaryotic translation initiation factor 5 | NM_001969 NM_183004 | 4,27336E-05 | -1,56265 |
| 11753619_s_at | 143244 1984 | EIF5A EIF5AL1 | eukaryotic translation initiation factor 5A eukaryotic translation initiation facto | NM_001099692 NM_001143760 NM_001143761 NM_001143762 NM_001970 | 0,000926049 | -1,50367 |
| 11760507_at | 1998 | ELF2 | E74-like factor 2 (ets domain transcription factor) | NM_006874 NM_201999 | 1,73036E-05 | -1,68491 |
| 11734985_at | 2005 | ELK4 | ELK4, ETS-domain protein (SRF accessory protein 1) | NM_001973 NM_021795 | 1,50057E-06 | -1,93982 |
| 11716653_x_at | 27436 | EML4 | echinoderm microtubule associated protein like 4 | NM_001145076 NM_019063 | 1,78562E-05 | -2,03988 |
| 11753779_x_at | 2029 | ENSA | endosulfine alpha | NM_004436 NM_207042 NM_207043 NM_207044 NM_207045 NM_207046 NM_ | 0,00161366 | -1,50493 |
| 11725127_at | 2033 | EP300 | E1A binding protein p300 | NM_001429 | 2,56294E-07 | -1,53911 |
| 11736740_a_at | 80314 | EPC1 | enhancer of polycomb homolog 1 (Drosophila) | NM_025209 | 6,32622E-07 | -1,71235 |
| 11752081_a_at | 85465 | EPT1 | ethanolaminephosphotransferase 1 (CDP-ethanolamine-specific) | NM_033505 | 1,33197E-12 | -2,25182 |
| 11718262_a_at | 2077 | ERF | Ets2 repressor factor | NM_006494 | 8,93445E-05 | -1,82559 |
| 11751747_a_at | 2078 | ERG | v-ets erythroblastosis virus E26 oncogene homolog (avian) | NM_001136154 NM_001136155 NM_004449 NM_182918 | 3,91093E-07 | -2,65407 |
| 11746760_a_at | 57222 | ERGIC1 | endoplasmic reticulum-golgi intermediate compartment (ERGIC) 1 | NM_001031711 | 1,04135E-05 | -1,67797 |
| 11739015_a_at | 57471 | ERMN | ermin, ERM-like protein | NM_001009959 NM_020711 | 8,42047E-10 | -2,03038 |
| 11726321_a_at | 30001 | ERO1L | ERO1-like (S. cerevisiae) | NM_014584 | 0,000033076 | -1,94899 |
| 11734858_at | 56605 | ERO1LB | ERO1-like beta (S. cerevisiae) | NM_019891 | 1,13625E-10 | -2,27536 |
| 11723909_at | 9700 | ESPL1 | extra spindle pole bodies homolog 1 (S. cerevisiae) | NM_012291 | 0,000564614 | -1,80668 |
| 11721448_a_at | 2107 | ETF1 | eukaryotic translation termination factor 1 | NM_004730 | 1,42648E-10 | -2,13579 |
| 11742988_s_at | 7430 | EZR | ezrin | NM_001111077 NM_003379 | 9,10235E-15 | -3,0406 |
| 11716002_at | 23197 | FAF2 | Fas associated factor family member 2 | NM_014613 | 3,6976E-09 | -1,69245 |
| 11721815_a_at | 9214 | FAIM3 | Fas apoptotic inhibitory molecule 3 | NM_001142472 NM_001142473 NM_005449 | 0,00813829 | -1,63938 |
| 11744085_at | 283991 | FAM100B | family with sequence similarity 100, member B | NM_182565 | 3,16964E-05 | -1,76533 |
| 11727569_at | 90268 | FAM105B | family with sequence similarity 105, member B | NM_138348 | 3,64723E-08 | -1,88532 |
| 11746414_a_at | 83641 | FAM107B | family with sequence similarity 107, member B | NM_031453 | 0,000656172 | -2,24066 |
| 11739176_at | 51104 | FAM108B1 | family with sequence similarity 108, member B1 | NM_001025780 NM_016014 | 3,01172E-08 | -1,6976 |
| 11753386_s_at | 145165 6767 729992 | FAM10A4 LOC729992 ST13 | ST13-like tumor suppressor similar to heat shock 70kD protein binding protein s | NM_003932 NR_002183 XR_038318 XR_038756 XR_039754 | 0,00277346 | -1,63964 |
| 11758454_s_at | 201627 | FAM116A | family with sequence similarity 116, member A | NM_152678 | 6,09062E-18 | -1,82239 |
| 11722923_a_at | 159090 | FAM122B | family with sequence similarity 122B | NM_001166599 NM_001166600 NM_001170756 NM_001170757 NM_145284 | 1,39631E-09 | -1,51183 |
| 11740777_a_at | 199786 | FAM129C | family with sequence similarity 129, member C | NM_001098524 NM_173544 | 4,38347E-09 | -2,89843 |
| 11746241_s_at | 257415 728640 | FAM133B LOC728640 | family with sequence similarity 133, member B family with sequence similarity 133, | NM_001040057 NM_152789 NR_027508 XR_015400 XR_015474 XR_038346 | 6,49143E-08 | -1,83005 |
| 11719737_a_at | 54463 | FAM134B | family with sequence similarity 134, member B | NM_001034850 NM_019000 | 0,0180829 | -1,53581 |
| 11721207_s_at | 57700 | FAM160B1 | family with sequence similarity 160, member B1 | NM_001135051 NM_020940 | 0,000117876 | -1,60917 |
| 11716045_s_at | 130074 | FAM168B | family with sequence similarity 168, member B | NM_001009993 | 8,13229E-12 | -1,55736 |
| 11754677_a_at | 26049 | FAM169A | family with sequence similarity 169, member A | NM_015566 | 0,000119104 | -1,64828 |
| 11742961_s_at | 131583 | FAM43A | family with sequence similarity 43, member A | NM_153690 | 2,17619E-05 | -2,53826 |
| 11724756_s_at | 51307 | FAM53C | family with sequence similarity 53, member C | NM_001135647 NM_016605 | 0,000175427 | -1,5577 |
| 11723747_a_at | 54478 | FAM64A | family with sequence similarity 64, member A | NM_019013 | 0,0185602 | -1,79513 |
| 11719895_x_at | 157769 | FAM91A1 | family with sequence similarity 91, member A1 | NM_144963 | 0,000234979 | -1,93437 |
| 11734736_a_at | 84188 | FAR1 | fatty acyl CoA reductase 1 | NM_032228 | 0,0101963 | -1,53313 |
| 11754628_a_at | 64319 | FBRS | fibrosin | NM_001105079 | 0,000654612 | -1,51362 |
| 11750639_s_at | 80204 | FBXO11 | F-box protein 11 | NM_012167 NM_018693 NM_025133 | 4,07979E-10 | -2,19261 |
| 11759149_at | 26273 | FBXO3 | F-box protein 3 | NM_012175 NM_033406 | 4,45513E-05 | -2,33798 |
| 11723321_at | 84085 | FBXO30 | F-box protein 30 | NM_032145 | 0,000367635 | -1,62966 |
| 11725457_at | 254170 | FBXO33 | F-box protein 33 | NM_203301 | 1,88172E-07 | -2,1049 |
| 11741731_a_at | 55030 | FBXO34 | F-box protein 34 | NM_017943 NM_152231 | 2,28146E-08 | -1,9324 |
| 11734988_s_at | 10116 | FEM1B | fem-1 homolog b (C. elegans) | NM_015322 | 0,000309532 | -1,84167 |
| 11723272_at | 56929 | FEM1C | fem-1 homolog c (C. elegans) | NM_020177 | 8,00855E-08 | -2,50354 |
| 11727301_a_at | 2260 | FGFR1 | fibroblast growth factor receptor 1 | NM_001174063 NM_001174064 NM_001174065 NM_001174066 NM_001174067 NM | 2,41086E-09 | -1,8268 |
| 11764055_at | 2332 | FMR1 | fragile X mental retardation 1 | NM_001185075 NM_001185076 NM_001185081 NM_001185082 NM_002024 NR_033699 NR_033700 | 5,61889E-09 | -3,01303 |
| 11717944_at | 23360 | FNBP4 | formin binding protein 4 | NM_015308 | 2,20557E-11 | -1,52846 |
| 11731054_s_at | 96459 | FNIP1 | folliculin interacting protein 1 | NM_001008738 NM_133372 | 1,37753E-05 | -1,71509 |
| 11763170_s_at | 2355 | FOSL2 | FOS-like antigen 2 | NM_005253 | 1,08917E-12 | -3,86742 |
| 11725441_a_at | 2305 | FOXM1 | forkhead box M1 | NM_021953 NM_202002 NM_202003 | 0,0135225 | -1,57874 |
| 11727929_a_at | 2308 | FOXO1 | forkhead box O1 | NM_002015 | 4,59068E-09 | -2,29669 |
| 11718890_x_at | 2309 | FOXO3 | forkhead box O3 | NM_001455 NM_201559 | 1,65166E-06 | -1,68763 |
| 11726384_at | 10129 | FRY | furry homolog (Drosophila) | NM_023037 | 0,000224242 | -1,61931 |
| 11751391_a_at | 84248 | FYTTD1 | forty-two-three domain containing 1 | NM_001011537 NM_032288 NR_027840 | 3,27537E-06 | -2,04267 |
| 11754074_s_at | 50486 | G0S2 | G0/G1switch 2 | NM_015714 | 0,0499213 | -1,91457 |
| 11716581_a_at | 9908 | G3BP2 | GTPase activating protein (SH3 domain) binding protein 2 | NM_012297 NM_203504 NM_203505 | 4,61419E-06 | -1,8943 |
| 11724867_a_at | 9846 | GAB2 | GRB2-associated binding protein 2 | NM_012296 NM_080491 | 8,0456E-13 | -2,33001 |
| 11758023_x_at | 23710 | GABARAPL1 | GABA(A) receptor-associated protein like 1 | NM_031412 | 0,00428633 | -1,88181 |
| 11722717_a_at | 2551 | GABPA | GA binding protein transcription factor, alpha subunit 60kDa | NM_002040 | 5,56929E-05 | -1,50142 |
| 11727466_a_at | 2553 | GABPB1 | GA binding protein transcription factor, beta subunit 1 | NM_002041 NM_005254 NM_016654 NM_016655 NM_181427 | 1,23514E-08 | -2,62697 |
| 11728763_x_at | 79690 | GAL3ST4 | galactose-3-O-sulfotransferase 4 | NM_024637 | 9,66246E-08 | -1,84561 |
| 11752004_s_at | 285190 400966 653489 727851 729540 729857 84220 9648 | GCC2 RGPD1 RGPD2 RGPD3 RGPD4 RGPD5 RGPD6 RGPD8 | GRIP and coiled-coil domain containing 2 RANBP2-like and GRIP domain containing 1 / | NM_001024457 NM_001037866 NM_001078170 NM_001123363 NM_001144013 NM | 0,000247233 | -2,283 |
| 11731313_a_at | 94104 | GCFC1 | GC-rich sequence DNA-binding factor 1 | NM_013329 NM_016631 NM_058191 NR_027873 | 1,63939E-05 | -1,75074 |
| 11741302_x_at | 145781 81488 | GCOM1 GRINL1A | GRINL1A complex locus glutamate receptor, ionotropic, N-methyl D-aspartate-like 1A | NM_001018090 NM_001018091 NM_001018100 NM_001018102 NM_015532 NM_15 | 1,40217E-05 | -1,88291 |
| 11720223_at | 2673 | GFPT1 | glutamine--fructose-6-phosphate transaminase 1 | NM_002056 | 2,85991E-09 | -1,65345 |
| 11746868_a_at | 9815 | GIT2 | G protein-coupled receptor kinase interacting ArfGAP 2 | NM_001135213 NM_001135214 NM_014776 NM_057169 NM_057170 NM_139201 | 7,38364E-08 | -1,589 |
| 11719592_at | 51228 | GLTP | glycolipid transfer protein | NM_016433 | 0,000243556 | -1,54239 |
| 11719594_at | 26205 | GMEB2 | glucocorticoid modulatory element binding protein 2 | NM_012384 | 1,56319E-12 | -2,10673 |
| 11727847_a_at | 2768 | GNA12 | guanine nucleotide binding protein (G protein) alpha 12 | NM_007353 | 7,91407E-05 | -1,5314 |
| 11748689_a_at | 10672 | GNA13 | guanine nucleotide binding protein (G protein), alpha 13 | NM_006572 | 0,00093341 | -1,59365 |
| 11753871_a_at | 2788 | GNG7 | guanine nucleotide binding protein (G protein), gamma 7 | NM_052847 | 4,78884E-08 | -1,77851 |
| 11751303_s_at | 26003 | GORASP2 | golgi reassembly stacking protein 2, 55kDa | NM_015530 | 7,0232E-11 | -1,53129 |
| 11718503_a_at | 56261 | GPCPD1 | glycerophosphocholine phosphodiesterase GDE1 homolog (S. cerevisiae) | NM_019593 | 0,00122467 | -1,5929 |
| 11745074_a_at | 2941 | GSTA4 | glutathione S-transferase alpha 4 | NM_001512 | 4,14397E-07 | -1,96457 |
| 11716986_a_at | 2959 | GTF2B | general transcription factor IIB | NM_001514 | 8,4867E-06 | -1,59407 |
| 11759958_x_at | 84163 | GTF2IRD2 | GTF2I repeat domain containing 2 | NM_173537 | 2,67843E-06 | -1,74896 |
| 11758112_s_at | 64412 | GZF1 | GDNF-inducible zinc finger protein 1 | NM_022482 | 5,20811E-05 | -1,9761 |
| 11743694_at | 94239 | H2AFV | H2A histone family, member V | NM_012412 NM_138635 NM_201436 NM_201516 NM_201517 | 3,76733E-08 | -2,31476 |
| 11758593_s_at | 3021 | H3F3B | H3 histone, family 3B (H3.3B) | NM_005324 | 4,88303E-05 | -1,55932 |
| 11754123_s_at | 3030 | HADHA | hydroxyacyl-CoA dehydrogenase/3-ketoacyl-CoA thiolase/enoyl-CoA hydratase (trifunctiona | NM_000182 | 0,00015016 | -1,68691 |
| 11728820_a_at | 55142 | HAUS2 | HAUS augmin-like complex, subunit 2 | NM_001130447 NM_018097 | 5,44956E-06 | -1,58671 |
| 11727265_a_at | 79441 | HAUS3 | HAUS augmin-like complex, subunit 3 | NM_024511 | 9,43581E-08 | -1,90481 |
| 11727488_s_at | 54801 | HAUS6 | HAUS augmin-like complex, subunit 6 | NM_017645 | 2,80378E-06 | -1,63749 |
| 11719898_s_at | 1839 | HBEGF | heparin-binding EGF-like growth factor | NM_001945 | 0,000307117 | -2,14691 |
| 11752897_a_at | 26959 | HBP1 | HMG-box transcription factor 1 | NM_012257 | 1,05883E-05 | -2,21458 |
| 11734203_at | 10767 | HBS1L | HBS1-like (S. cerevisiae) | NM_001145158 NM_001145207 NM_006620 | 0,00011261 | -1,77476 |
| 11719433_at | 51696 | HECA | headcase homolog (Drosophila) | NM_016217 | 2,16906E-10 | -2,44733 |
| 11744225_at | 8925 | HERC1 | hect (homologous to the E6-AP (UBE3A) carboxyl terminus) domain and RCC1 (CHC1)-like do | NM_003922 | 5,23869E-09 | -1,60741 |
| 11761495_at | 8916 | HERC3 | hect domain and RLD 3 | NM_014606 | 4,46447E-05 | -1,86914 |
| 11724028_a_at | 23119 | HIC2 | hypermethylated in cancer 2 | NM_015094 | 2,20347E-06 | -1,69041 |
| 11749123_a_at | 3091 | HIF1A | hypoxia inducible factor 1, alpha subunit (basic helix-loop-helix transcription factor) | NM_001530 NM_181054 | 6,51984E-06 | -2,32942 |
| 11722239_a_at | 204851 | HIPK1 | homeodomain interacting protein kinase 1 | NM_152696 NM_181358 NM_198268 NM_198269 | 5,06091E-09 | -1,51914 |
| 11746804_x_at | 3119 | HLA-DQB1 | major histocompatibility complex, class II, DQ beta 1 | NM_002123 | 0,0104357 | -1,72995 |
| 11724271_a_at | 3131 | HLF | hepatic leukemia factor | NM_002126 | 0,0205434 | -1,51572 |
| 11762147_at | 8091 | HMGA2 | high mobility group AT-hook 2 | NM_003483 NM_003484 | 0,000166013 | -3,02589 |
| 11754183_s_at | 3149 | HMGB3 | high-mobility group box 3 | NM_005342 | 4,19423E-19 | -2,50522 |
| 11733879_a_at | 10042 | HMGXB4 | HMG box domain containing 4 | NM_001003681 NM_005487 NM_014250 NR_027780 | 9,29249E-16 | -1,98971 |
| 11732247_s_at | 3183 | HNRNPC | heterogeneous nuclear ribonucleoprotein C (C1/C2) | NM_001077442 NM_001077443 NM_004500 NM_031314 | 6,69256E-11 | -1,67045 |
| 11760952_x_at | 3184 | HNRNPD | heterogeneous nuclear ribonucleoprotein D (AU-rich element RNA binding protein 1, 37kDa | NM_001003810 NM_002138 NM_031369 NM_031370 | 0,00450136 | -1,52826 |
| 11749449_s_at | 3188 | HNRNPH2 | heterogeneous nuclear ribonucleoprotein H2 (H') | NM_001032393 NM_019597 | 1,63496E-06 | -1,66666 |
| 11755112_s_at | 11100 | HNRNPUL1 | heterogeneous nuclear ribonucleoprotein U-like 1 | NM_007040 NM_144732 | 1,4532E-09 | -1,53214 |
| 11722747_at | 84376 | HOOK3 | hook homolog 3 (Drosophila) | NM_032410 | 1,93831E-07 | -1,51528 |
| 11720525_a_at | 89781 | HPS4 | Hermansky-Pudlak syndrome 4 | NM_022081 NM_152841 | 4,27694E-12 | -1,68639 |
| 11719211_a_at | 3298 | HSF2 | heat shock transcription factor 2 | NM_001135564 NM_004506 | 7,26523E-08 | -2,11079 |
| 11719069_s_at | 6782 | HSPA13 | heat shock protein 70kDa family, member 13 | NM_006948 | 2,63672E-05 | -2,41634 |
| 11725933_at | 3309 | HSPA5 | heat shock 70kDa protein 5 (glucose-regulated protein, 78kDa) | NM_005347 | 0,0399508 | -1,61517 |
| 11752595_a_at | 3313 | HSPA9 | heat shock 70kDa protein 9 (mortalin) | NM_004134 | 2,67571E-05 | -1,5109 |
| 11747201_a_at | 3423 | IDS | iduronate 2-sulfatase | NM_000202 NM_001166550 NM_006123 | 2,01465E-14 | -3,75346 |
| 11735990_x_at | 3512 | IGJ | immunoglobulin J polypeptide, linker protein for immunoglobulin alpha and mu polypeptid | NM_144646 | 2,65176E-11 | -7,18028 |
| 11725198_at | 3552 | IL1A | interleukin 1, alpha | NM_000575 | 0,0257357 | -1,53449 |
| 11759038_at | 3572 | IL6ST | interleukin 6 signal transducer (gp130, oncostatin M receptor) | NM_002184 NM_175767 | 0,00104207 | -2,12855 |
| 11740781_a_at | 3609 | ILF3 | interleukin enhancer binding factor 3, 90kDa | NM_001137673 NM_004516 NM_012218 NM_017620 NM_153464 | 2,63553E-09 | -1,9762 |
| 11716854_s_at | 54928 | IMPAD1 | inositol monophosphatase domain containing 1 | NM_017813 | 1,48503E-11 | -2,11399 |
| 11727959_s_at | 3621 | ING1 | inhibitor of growth family, member 1 | NM_005537 NM_198217 NM_198218 NM_198219 | 3,97802E-06 | -1,58036 |
| 11728784_a_at | 54556 | ING3 | inhibitor of growth family, member 3 | NM_019071 NM_198267 | 2,32049E-05 | -1,5805 |
| 11744573_a_at | 54617 | INO80 | INO80 homolog (S. cerevisiae) | NM_017553 | 6,90547E-13 | -1,52969 |
| 11726895_a_at | 11213 | IRAK3 | interleukin-1 receptor-associated kinase 3 | NM_001142523 NM_007199 | 6,74283E-05 | -1,87471 |
| 11721365_a_at | 3658 | IREB2 | iron-responsive element binding protein 2 | NM_004136 | 6,33527E-08 | -2,11172 |
| 11717508_at | 3662 | IRF4 | interferon regulatory factor 4 | NM_002460 | 1,79248E-10 | -2,71238 |
| 11739415_at | 126298 | IRGQ | immunity-related GTPase family, Q | NM_001007561 | 2,2747E-06 | -3,11363 |
| 11718013_at | 8660 | IRS2 | insulin receptor substrate 2 | NM_003749 | 6,72156E-07 | -2,01552 |
| 11731493_at | 83737 | ITCH | itchy E3 ubiquitin protein ligase homolog (mouse) | NM_031483 | 2,58207E-09 | -2,38939 |
| 11763773_a_at | 81618 | ITM2C | integral membrane protein 2C | NM_001012514 NM_001012516 NM_030926 | 0,000116695 | -1,96764 |
| 11720757_a_at | 10625 | IVNS1ABP | influenza virus NS1A binding protein | NM_006469 | 8,75757E-05 | -1,71504 |
| 11756294_a_at | 58494 | JAM2 | junctional adhesion molecule 2 | NM_021219 | 1,70111E-06 | -1,54579 |
| 11720011_at | 3720 | JARID2 | jumonji, AT rich interactive domain 2 | NM_004973 | 3,86613E-10 | -2,50488 |
| 11746223_a_at | 221037 | JMJD1C | jumonji domain containing 1C | NM_004241 NM_032776 | 2,75718E-12 | -2,42912 |
| 11744289_a_at | 23210 | JMJD6 | jumonji domain containing 6 | NM_001081461 NM_015167 | 0,000155794 | -1,92893 |
| 11747116_a_at | 133746 | JMY | junction mediating and regulatory protein, p53 cofactor | NM_152405 | 0,000121796 | -1,76327 |
| 11755160_a_at | 9929 | JOSD1 | Josephin domain containing 1 | NM_014876 | 1,04236E-09 | -1,64434 |
| 11757969_x_at | 3727 | JUND | jun D proto-oncogene | NM_005354 | 0,00278443 | -1,50055 |
| 11716602_at | 25948 | KBTBD2 | kelch repeat and BTB (POZ) domain containing 2 | NM_015483 | 0,00120042 | -2,07039 |
| 11718518_at | 56888 | KCMF1 | potassium channel modulatory factor 1 | NM_020122 | 1,83078E-08 | -1,5918 |
| 11727317_at | 23630 | KCNE1L | KCNE1-like | NM_012282 | 6,85665E-07 | -2,562 |
| 11741932_a_at | 283518 | KCNRG | potassium channel regulator | NM_173605 NM_199464 | 5,70375E-11 | -1,5956 |
| 11758648_s_at | 83892 | KCTD10 | potassium channel tetramerisation domain containing 10 | NM_031954 | 1,70199E-08 | -1,69687 |
| 11758284_s_at | 54793 | KCTD9 | potassium channel tetramerisation domain containing 9 | NM_017634 | 4,52553E-08 | -1,55699 |
| 11717167_a_at | 22992 | KDM2A | lysine (K)-specific demethylase 2A | NM_012308 NR_027473 | 1,34969E-10 | -1,74295 |
| 11752163_a_at | 84678 | KDM2B | lysine (K)-specific demethylase 2B | NM_001005366 NM_032590 | 3,20915E-09 | -1,61722 |
| 11716573_a_at | 55818 | KDM3A | lysine (K)-specific demethylase 3A | NM_001146688 NM_018433 | 2,40875E-08 | -2,01926 |
| 11743523_a_at | 5927 | KDM5A | lysine (K)-specific demethylase 5A | NM_001042603 NM_005056 | 6,08927E-09 | -1,5273 |
| 11743085_a_at | 7403 | KDM6A | lysine (K)-specific demethylase 6A | NM_021140 | 2,04275E-07 | -2,00433 |
| 11721165_a_at | 23351 | KHNYN | KH and NYN domain containing | NM_015299 | 1,58412E-09 | -1,62662 |
| 11721808_x_at | 9798 | KIAA0174 | KIAA0174 | NM_014761 | 7,56699E-06 | -2,00908 |
| 11728702_s_at | 9778 | KIAA0232 | KIAA0232 | NM_001100590 NM_014743 | 7,35868E-10 | -1,9108 |
| 11725489_x_at | 22863 | KIAA0831 | KIAA0831 | NM_014924 | 9,15596E-10 | -2,02783 |
| 11718565_at | 57189 | KIAA1147 | KIAA1147 | NM_001080392 | 1,5382E-07 | -1,55242 |
| 11729431_a_at | 27143 | KIAA1274 | KIAA1274 | NM_014431 | 9,11287E-05 | -1,5644 |
| 11736612_x_at | 57589 | KIAA1432 | KIAA1432 | NM_001135920 NM_020829 | 8,58301E-18 | -2,3011 |
| 11727234_at | 57724 | KIAA1632 | KIAA1632 | NM_020964 | 9,8798E-08 | -1,7344 |
| 11755253_a_at | 205717 | KIAA2018 | KIAA2018 | NM_001009899 | 2,14624E-09 | -1,70501 |
| 11741537_a_at | 63971 | KIF13A | kinesin family member 13A | NM_001105566 NM_001105567 NM_001105568 NM_022113 | 3,27893E-06 | -1,58588 |
| 11731887_at | 9928 | KIF14 | kinesin family member 14 | NM_014875 | 0,00294777 | -1,92576 |
| 11756918_a_at | 9493 | KIF23 | kinesin family member 23 | NM_004856 NM_138555 | 0,0125212 | -1,53678 |
| 11724209_a_at | 3833 | KIFC1 | kinesin family member C1 | NM_002263 | 0,00127082 | -1,6557 |
| 11743682_s_at | 7071 | KLF10 | Kruppel-like factor 10 | NM_001032282 NM_005655 | 0,00118325 | -1,69436 |
| 11727145_s_at | 8462 | KLF11 | Kruppel-like factor 11 | NM_001177716 NM_001177718 NM_003597 | 0,00966761 | -1,68579 |
| 11718160_at | 51621 | KLF13 | Kruppel-like factor 13 | NM_015995 | 0,000239003 | -1,55559 |
| 11759749_at | 51274 | KLF3 | Kruppel-like factor 3 (basic) | NM_016531 | 1,90357E-07 | -3,6525 |
| 11759428_a_at | 8609 | KLF7 | Kruppel-like factor 7 (ubiquitous) | NM_003709 | 2,72786E-05 | -1,55588 |
| 11725569_at | 80311 | KLHL15 | kelch-like 15 (Drosophila) | NM_030624 | 0,000225677 | -1,69254 |
| 11759656_a_at | 55975 | KLHL7 | kelch-like 7 (Drosophila) | NM_001031710 NM_001172428 NM_018846 NR_033328 NR_033329 | 3,61691E-08 | -2,38583 |
| 11718200_at | 3839 | KPNA3 | karyopherin alpha 3 (importin alpha 4) | NM_002267 | 5,35827E-09 | -1,6991 |
| 11749630_a_at | 11103 | KRR1 | KRR1, small subunit (SSU) processome component, homolog (yeast) | NM_007043 | 3,39493E-08 | -1,85435 |
| 11725085_a_at | 55915 | LANCL2 | LanC lantibiotic synthetase component C-like 2 (bacterial) | NM_018697 | 3,61979E-09 | -1,60749 |
| 11753275_a_at | 9741 | LAPTM4A | lysosomal protein transmembrane 4 alpha | NM_014713 | 0,000142937 | -1,71311 |
| 11723907_a_at | 55132 | LARP1B | La ribonucleoprotein domain family, member 1B | NM_018078 NM_032239 NM_178043 | 3,02439E-10 | -1,96756 |
| 11721738_a_at | 26524 | LATS2 | LATS, large tumor suppressor, homolog 2 (Drosophila) | NM_014572 | 8,63784E-07 | -1,86938 |
| 11751573_a_at | 3930 | LBR | lamin B receptor | NM_002296 NM_194442 | 2,81922E-05 | -2,71043 |
| 11728429_a_at | 84458 | LCOR | ligand dependent nuclear receptor corepressor | NM_001170765 NM_001170766 NM_032440 | 1,98765E-06 | -1,57045 |
| 11726333_s_at | 51176 | LEF1 | lymphoid enhancer-binding factor 1 | NM_001130713 NM_001130714 NM_001166119 NM_016269 | 0,00265083 | -3,03287 |
| 11728605_s_at | 3987 440895 96626 | LIMS1 LIMS3 LOC440895 | LIM and senescent cell antigen-like domains 1 LIM and senescent cell antigen-like d | NM_004987 NM_033514 NR_027143 NR_027467 | 0,00191668 | -1,72291 |
| 11740773_s_at | 132660 | LIN54 | lin-54 homolog (C. elegans) | NM_001115007 NM_001115008 NM_194282 | 1,29464E-09 | -1,55652 |
| 11757883_s_at | 6047 644006 | LOC644006 RNF4 | hypothetical LOC644006 ring finger protein 4 | NM_002938 XM_001722645 XM_929433 XM_939111 | 5,8019E-09 | -1,64151 |
| 11721014_s_at | 646517 9589 | LOC646517 WTAP | similar to Wilms tumour 1-associating protein Wilms tumor 1 associated protein | NM_004906 NM_152857 NM_152858 XR_016074 XR_017669 XR_039014 | 4,51075E-07 | -2,1134 |
| 11731076_a_at | 91694 | LONRF1 | LON peptidase N-terminal domain and ring finger 1 | NM_152271 | 1,51286E-06 | -1,94336 |
| 11755110_a_at | 26018 | LRIG1 | leucine-rich repeats and immunoglobulin-like domains 1 | NM_015541 | 0,00486273 | -2,1104 |
| 11754389_x_at | 255252 | LRRC57 | leucine rich repeat containing 57 | NM_153260 | 7,97534E-05 | -1,55826 |
| 11757458_s_at | 56262 | LRRC8A | leucine rich repeat containing 8 family, member A | NM_001127244 NM_001127245 NM_019594 | 0,0003965 | -1,5358 |
| 11739657_a_at | 4050 | LTB | lymphotoxin beta (TNF superfamily, member 3) | NM_002341 NM_009588 | 1,47579E-05 | -2,10104 |
| 11751835_a_at | 84946 | LTV1 | LTV1 homolog (S. cerevisiae) | NM_032860 | 0,000783995 | -1,68706 |
| 11728263_at | 55692 | LUC7L | LUC7-like (S. cerevisiae) | NM_018032 NM_201412 | 6,38913E-09 | -1,85895 |
| 11724314_a_at | 7798 | LUZP1 | leucine zipper protein 1 | NM_001142546 NM_033631 | 0,000520118 | -1,57616 |
| 11724678_a_at | 9450 | LY86 | lymphocyte antigen 86 | NM_004271 | 1,09986E-06 | -2,55738 |
| 11726611_x_at | 23764 | MAFF | v-maf musculoaponeurotic fibrosarcoma oncogene homolog F (avian) | NM_001161572 NM_001161573 NM_001161574 NM_012323 NM_152878 | 0,00968146 | -2,04766 |
| 11758538_s_at | 4097 | MAFG | v-maf musculoaponeurotic fibrosarcoma oncogene homolog G (avian) | NM_002359 NM_032711 | 2,49219E-05 | -1,52003 |
| 11719388_x_at | 7975 | MAFK | v-maf musculoaponeurotic fibrosarcoma oncogene homolog K (avian) | NM_002360 | 3,24353E-06 | -1,86182 |
| 11753222_s_at | 84061 | MAGT1 | magnesium transporter 1 | NM_032121 | 1,223E-08 | -2,40981 |
| 11736101_at | 4121 | MAN1A1 | mannosidase, alpha, class 1A, member 1 | NM_005907 | 0,000396961 | -1,70258 |
| 11738983_x_at | 81631 | MAP1LC3B | microtubule-associated protein 1 light chain 3 beta | NM_022818 | 0,000019575 | -2,01572 |
| 11759296_at | 10746 | MAP3K2 | mitogen-activated protein kinase kinase kinase 2 | NM_006609 | 2,04095E-06 | -2,38955 |
| 11720218_a_at | 4215 | MAP3K3 | mitogen-activated protein kinase kinase kinase 3 | NM_002401 NM_203351 | 4,42319E-05 | -1,50076 |
| 11721362_a_at | 8491 | MAP4K3 | mitogen-activated protein kinase kinase kinase kinase 3 | NM_003618 | 7,63311E-06 | -1,55422 |
| 11746471_s_at | 9448 | MAP4K4 | mitogen-activated protein kinase kinase kinase kinase 4 | NM_004834 NM_145686 NM_145687 | 6,0522E-07 | -1,59931 |
| 11755571_a_at | 55700 | MAP7D1 | MAP7 domain containing 1 | NM_018067 | 8,16947E-08 | -1,79055 |
| 11759946_at | 1432 | MAPK14 | mitogen-activated protein kinase 14 | NM_001315 NM_139012 NM_139013 NM_139014 | 0,000146754 | -1,73439 |
| 11722796_a_at | 5597 | MAPK6 | mitogen-activated protein kinase 6 | NM_002748 | 0,000346811 | -1,68393 |
| 11730896_a_at | 9261 | MAPKAPK2 | mitogen-activated protein kinase-activated protein kinase 2 | NM_004759 NM_032960 | 2,84227E-09 | -2,18506 |
| 11715586_at | 22919 | MAPRE1 | microtubule-associated protein, RP/EB family, member 1 | NM_012325 | 9,48964E-09 | -1,8155 |
| 11743138_at | 54708 | MARCH5 | membrane-associated ring finger (C3HC4) 5 | NM_017824 | 2,97235E-11 | -2,56456 |
| 11717746_a_at | 10299 | MARCH6 | membrane-associated ring finger (C3HC4) 6 | NM_005885 | 1,63372E-06 | -1,67627 |
| 11723771_a_at | 84930 | MASTL | microtubule associated serine/threonine kinase-like | NM_001172303 NM_001172304 NM_032844 | 4,33053E-10 | -2,13728 |
| 11728150_at | 4149 | MAX | MYC associated factor X | NM_002382 NM_145112 NM_145113 NM_145114 NM_145116 NM_197957 | 0,0120635 | -1,63393 |
| 11731288_a_at | 8932 | MBD2 | methyl-CpG binding domain protein 2 | NM_003927 NM_015832 | 6,69654E-05 | -1,75775 |
| 11748317_s_at | 90411 | MCFD2 | multiple coagulation factor deficiency 2 | NM_001171506 NM_001171507 NM_001171508 NM_001171509 NM_001171510 NM | 4,01472E-09 | -2,59272 |
| 11715484_a_at | 4170 | MCL1 | myeloid cell leukemia sequence 1 (BCL2-related) | NM_021960 NM_182763 | 0,000180762 | -2,2103 |
| 11731565_x_at | 4193 | MDM2 | Mdm2 p53 binding protein homolog (mouse) | NM_001145336 NM_001145337 NM_001145339 NM_001145340 NM_002392 NM_00 | 5,00917E-07 | -1,79256 |
| 11721912_at | 4194 | MDM4 | Mdm4 p53 binding protein homolog (mouse) | NM_002393 NR_024171 | 2,77409E-14 | -1,94655 |
| 11732947_a_at | 9439 | MED23 | mediator complex subunit 23 | NM_004830 NM_015979 | 4,75861E-10 | -1,58036 |
| 11754467_s_at | 9441 | MED26 | mediator complex subunit 26 | NM_004831 | 1,0641E-06 | -1,76066 |
| 11760463_at | 10001 | MED6 | mediator complex subunit 6 | NM_005466 | 6,00706E-08 | -2,32666 |
| 11718096_a_at | 4205 | MEF2A | myocyte enhancer factor 2A | NM_001130926 NM_001130927 NM_001130928 NM_001171894 NM_005587 | 4,49417E-07 | -1,8125 |
| 11749473_a_at | 4209 | MEF2D | myocyte enhancer factor 2D | NM_005920 | 6,4099E-12 | -2,69859 |
| 11736383_a_at | 399818 | METTL10 | methyltransferase like 10 | NM_212554 | 4,48533E-09 | -1,57638 |
| 11758191_s_at | 51320 | MEX3C | mex-3 homolog C (C. elegans) | NM_016626 | 1,09359E-08 | -2,08529 |
| 11715706_x_at | 10724 | MGEA5 | meningioma expressed antigen 5 (hyaluronidase) | NM_001142434 NM_012215 | 9,77079E-07 | -2,0251 |
| 11722387_at | 90007 | MIDN | midnolin | NM_177401 | 0,000117408 | -1,6146 |
| 11740072_s_at | 57708 | MIER1 | mesoderm induction early response 1 homolog (Xenopus laevis) | NM_001077700 NM_001077701 NM_001077702 NM_001077703 NM_001077704 NM | 3,5352E-06 | -1,55792 |
| 11725769_at | 166968 | MIER3 | mesoderm induction early response 1, family member 3 | NM_152622 | 3,23318E-08 | -1,82315 |
| 11721144_a_at | 4288 | MKI67 | antigen identified by monoclonal antibody Ki-67 | NM_001145966 NM_002417 | 0,00636996 | -1,88657 |
| 11739146_a_at | 2872 | MKNK2 | MAP kinase interacting serine/threonine kinase 2 | NM_017572 NM_199054 | 2,04098E-06 | -1,72105 |
| 11759830_at | 55904 | MLL5 | myeloid/lymphoid or mixed-lineage leukemia 5 (trithorax homolog, Drosophila) | NM_018682 NM_182931 | 2,73212E-08 | -2,72878 |
| 11741562_a_at | 4311 | MME | membrane metallo-endopeptidase | NM_000902 NM_007287 NM_007288 NM_007289 | 1,71688E-11 | -5,281 |
| 11719168_at | 64112 | MOAP1 | modulator of apoptosis 1 | NM_022151 | 4,0065E-06 | -1,51943 |
| 11739834_s_at | 25843 | MOBKL3 | MOB1, Mps One Binder kinase activator-like 3 (yeast) | NM_001100819 NM_015387 NM_199482 | 5,68322E-06 | -1,60653 |
| 11745636_a_at | 23041 | MON2 | MON2 homolog (S. cerevisiae) | NM_015026 | 2,91491E-06 | -1,91166 |
| 11755858_a_at | 931 | MS4A1 | membrane-spanning 4-domains, subfamily A, member 1 | NM_021950 NM_152866 | 5,73911E-06 | -3,36435 |
| 11726609_at | 22823 | MTF2 | metal response element binding transcription factor 2 | NM_001164391 NM_001164392 NM_001164393 NM_007358 | 4,28665E-11 | -1,81281 |
| 11755511_x_at | 9633 | MTL5 | metallothionein-like 5, testis-specific (tesmin) | NM_001039656 NM_004923 | 0,000165436 | -1,68754 |
| 11756307_a_at | 54893 | MTMR10 | myotubularin related protein 10 | NM_017762 | 2,35051E-06 | -1,86849 |
| 11755887_a_at | 54545 | MTMR12 | myotubularin related protein 12 | NM_001040446 | 2,31079E-09 | -1,70657 |
| 11721021_a_at | 8897 | MTMR3 | myotubularin related protein 3 | NM_021090 NM_153050 NM_153051 | 7,53277E-06 | -1,51284 |
| 11723203_s_at | 9107 | MTMR6 | myotubularin related protein 6 | NM_004685 | 3,18319E-13 | -2,39998 |
| 11739183_a_at | 54516 | MTRF1L | mitochondrial translational release factor 1-like | NM_001114184 NM_019041 | 1,22303E-06 | -2,05464 |
| 11723115_s_at | 345778 | MTX3 | metaxin 3 | NM_001010891 NM_001167741 | 0,00936489 | -1,65516 |
| 11762666_at | 55745 | MUDENG | MU-2/AP1M2 domain containing, death-inducing | NM_018229 NR_026895 | 1,01894E-05 | -1,59158 |
| 11750016_a_at | 4084 | MXD1 | MAX dimerization protein 1 | NM_002357 | 1,44912E-07 | -3,42587 |
| 11725748_a_at | 83463 | MXD3 | MAX dimerization protein 3 | NM_001142935 NM_031300 | 2,20688E-07 | -1,5938 |
| 11763312_at | 10608 | MXD4 | MAX dimerization protein 4 | NM_006454 | 0,000134879 | -1,55682 |
| 11724358_s_at | 4644 | MYO5A | myosin VA (heavy chain 12, myoxin) | NM_000259 NM_001142495 | 3,3382E-06 | -1,55826 |
| 11759877_at | 55930 | MYO5C | myosin VC | NM_018728 | 4,14032E-07 | -1,85319 |
| 11726636_s_at | 7994 | MYST3 | MYST histone acetyltransferase (monocytic leukemia) 3 | NM_001099412 NM_001099413 NM_006766 | 1,8459E-11 | -1,60695 |
| 11735202_a_at | 55728 | N4BP2 | NEDD4 binding protein 2 | NM_018177 | 0,00494577 | -1,60096 |
| 11733714_a_at | 79612 | NAA16 | N(alpha)-acetyltransferase 16, NatA auxiliary subunit | NM_001110798 NM_018527 NM_024561 | 8,59996E-08 | -1,66207 |
| 11746938_a_at | 122830 | NAA30 | N(alpha)-acetyltransferase 30, NatC catalytic subunit | NM_001011713 | 2,87916E-06 | -1,56852 |
| 11756128_a_at | 80218 | NAA50 | N(alpha)-acetyltransferase 50, NatE catalytic subunit | NM_025146 | 2,54062E-08 | -1,75738 |
| 11762777_a_at | 4673 | NAP1L1 | nucleosome assembly protein 1-like 1 | NM_004537 NM_139207 | 6,85087E-10 | -1,83029 |
| 11718331_a_at | 63908 | NAPB | N-ethylmaleimide-sensitive factor attachment protein, beta | NM_022080 | 0,00378196 | -1,56572 |
| 11758328_s_at | 8202 | NCOA3 | nuclear receptor coactivator 3 | NM_001174087 NM_001174088 NM_006534 NM_181659 | 5,42729E-07 | -2,06004 |
| 11717573_s_at | 25977 | NECAP1 | NECAP endocytosis associated 1 | NM_015509 NR_024260 | 5,22382E-07 | -2,01267 |
| 11744915_a_at | 257194 | NEGR1 | neuronal growth regulator 1 | NM_173808 | 0,010323 | -1,64712 |
| 11722251_a_at | 4751 | NEK2 | NIMA (never in mitosis gene a)-related kinase 2 | NM_002497 | 0,0347364 | -1,78287 |
| 11738447_a_at | 10725 | NFAT5 | nuclear factor of activated T-cells 5, tonicity-responsive | NM_001113178 NM_006599 NM_138713 NM_138714 NM_173214 | 8,14917E-09 | -1,69362 |
| 11743007_at | 4794 | NFKBIE | nuclear factor of kappa light polypeptide gene enhancer in B-cells inhibitor, epsilon | NM_004556 | 0,00405399 | -1,98012 |
| 11722629_a_at | 25983 | NGDN | neuroguidin, EIF4E binding protein | NM_001042635 NM_015514 | 7,10151E-11 | -1,53023 |
| 11721744_a_at | 22795 | NID2 | nidogen 2 (osteonidogen) | NM_007361 | 0,00497535 | -1,79261 |
| 11716717_a_at | 4814 | NINJ1 | ninjurin 1 | NM_004148 | 0,00141706 | -1,79735 |
| 11730269_at | 25836 | NIPBL | Nipped-B homolog (Drosophila) | NM_015384 NM_133433 | 6,49143E-08 | -1,59303 |
| 11745029_a_at | 55922 | NKRF | NFKB repressing factor | NM_001173487 NM_001173488 NM_017544 | 9,49427E-11 | -1,73648 |
| 11718535_at | 4820 | NKTR | natural killer-tumor recognition sequence | NM_005385 | 2,05343E-06 | -1,53791 |
| 11738523_a_at | 114548 | NLRP3 | NLR family, pyrin domain containing 3 | NM_001079821 NM_001127461 NM_001127462 NM_004895 NM_183395 | 0,00151051 | -1,66691 |
| 11720005_at | 4885 | NPTX2 | neuronal pentraxin II | NM_002523 | 0,000104235 | -1,81836 |
| 11742029_a_at | 9975 | NR1D2 | nuclear receptor subfamily 1, group D, member 2 | NM_001145425 NM_005126 | 0,000255733 | -1,73582 |
| 11763443_at | 7181 | NR2C1 | Nuclear receptor subfamily 2, group C, member 1 | NM_001032287 NM_001127362 NM_003297 | 3,20549E-07 | -1,87468 |
| 11743739_a_at | 2908 | NR3C1 | nuclear receptor subfamily 3, group C, member 1 (glucocorticoid receptor) | NM_000176 NM_001018074 NM_001018075 NM_001018076 NM_001018077 NM_00 | 1,91221E-09 | -1,61683 |
| 11729058_s_at | 8013 | NR4A3 | nuclear receptor subfamily 4, group A, member 3 | NM_006981 NM_173198 NM_173199 NM_173200 | 1,97787E-06 | -2,48686 |
| 11741386_a_at | 4899 | NRF1 | nuclear respiratory factor 1 | NM_001040110 NM_005011 | 4,59313E-09 | -1,56599 |
| 11760519_at | 64324 | NSD1 | nuclear receptor binding SET domain protein 1 | NM_022455 NM_172349 | 0,00204969 | -1,52268 |
| 11722568_at | 22978 | NT5C2 | 5'-nucleotidase, cytosolic II | NM_001134373 NM_012229 | 5,07552E-09 | -1,98676 |
| 11718962_a_at | 57532 | NUFIP2 | nuclear fragile X mental retardation protein interacting protein 2 | NM_020772 | 0,000101181 | -1,79198 |
| 11719636_a_at | 9972 | NUP153 | nucleoporin 153kDa | NM_005124 | 6,15353E-13 | -1,74652 |
| 11743662_a_at | 53371 | NUP54 | nucleoporin 54kDa | NM_017426 | 3,53217E-10 | -1,77624 |
| 11731268_a_at | 4928 | NUP98 | nucleoporin 98kDa | NM_005387 NM_016320 NM_139131 NM_139132 | 5,08661E-09 | -2,53239 |
| 11756569_a_at | 51203 | NUSAP1 | nucleolar and spindle associated protein 1 | NM_001129897 NM_016359 NM_018454 | 0,0050272 | -1,72911 |
| 11732403_a_at | 79627 | OGFRL1 | opioid growth factor receptor-like 1 | NM_024576 | 0,00703172 | -1,66175 |
| 11743529_a_at | 4976 | OPA1 | optic atrophy 1 (autosomal dominant) | NM_015560 NM_130831 NM_130832 NM_130833 NM_130834 NM_130835 NM_ | 7,1436E-07 | -1,73042 |
| 11728751_a_at | 5007 | OSBP | oxysterol binding protein | NM_002556 | 9,82041E-08 | -1,69226 |
| 11741895_a_at | 54726 | OTUD4 | OTU domain containing 4 | NM_001102653 NM_017493 NM_199324 | 2,59709E-08 | -1,58478 |
| 11733632_s_at | 9934 | P2RY14 | purinergic receptor P2Y, G-protein coupled, 14 | NM_001081455 NM_014879 | 0,000484602 | -2,86179 |
| 11717118_a_at | 8974 | P4HA2 | prolyl 4-hydroxylase, alpha polypeptide II | NM_001017973 NM_001017974 NM_001142598 NM_001142599 NM_004199 | 0,00244077 | -1,77606 |
| 11716153_a_at | 5048 | PAFAH1B1 | platelet-activating factor acetylhydrolase 1b, regulatory subunit 1 (45kDa) | NM_000430 | 1,73783E-07 | -1,57438 |
| 11762985_a_at | 5049 | PAFAH1B2 | platelet-activating factor acetylhydrolase 1b, catalytic subunit 2 (30kDa) | NM_001184746 NM_001184747 NM_001184748 NM_002572 | 1,48643E-06 | -1,8507 |
| 11724378_s_at | 55824 | PAG1 | phosphoprotein associated with glycosphingolipid microdomains 1 | NM_018440 | 0,0182971 | -2,12773 |
| 11733936_a_at | 10605 | PAIP1 | poly(A) binding protein interacting protein 1 | NM_006451 NM_182789 NM_183323 | 1,49708E-06 | -1,55212 |
| 11753743_x_at | 51247 | PAIP2 | poly(A) binding protein interacting protein 2 | NM_001033112 NM_016480 | 0,000033138 | -1,5216 |
| 11733083_s_at | 23022 | PALLD | palladin, cytoskeletal associated protein | NM_001166108 NM_001166109 NM_001166110 NM_016081 | 0,000369543 | -1,79656 |
| 11744392_a_at | 10914 | PAPOLA | poly(A) polymerase alpha | NM_032632 | 3,24269E-05 | -1,59634 |
| 11748434_a_at | 10038 | PARP2 | poly (ADP-ribose) polymerase 2 | NM_001042618 NM_005484 | 2,08187E-08 | -1,61992 |
| 11759592_at | 84333 | PCGF5 | polycomb group ring finger 5 | NM_032373 | 2,60558E-05 | -1,79504 |
| 11724095_a_at | 5142 | PDE4B | phosphodiesterase 4B, cAMP-specific (phosphodiesterase E4 dunce homolog, Drosophila) | NM_001037339 NM_001037340 NM_001037341 NM_002600 | 2,96229E-11 | -5,23273 |
| 11724953_a_at | 149420 | PDIK1L | PDLIM1 interacting kinase 1 like | NM_152835 NR_026685 NR_026686 | 3,31068E-07 | -1,54315 |
| 11733060_a_at | 57162 | PELI1 | pellino homolog 1 (Drosophila) | NM_020651 | 0,0230502 | -1,73788 |
| 11717168_a_at | 5187 | PER1 | period homolog 1 (Drosophila) | NM_002616 | 1,7627E-07 | -2,84193 |
| 11758315_s_at | 8864 | PER2 | period homolog 2 (Drosophila) | NM_022817 | 8,51784E-14 | -2,13005 |
| 11717732_s_at | 5209 | PFKFB3 | 6-phosphofructo-2-kinase/fructose-2,6-biphosphatase 3 | NM_001145443 NM_004566 | 0,00480709 | -1,70783 |
| 11733872_x_at | 5230 | PGK1 | phosphoglycerate kinase 1 | NM_000291 | 2,62868E-08 | -1,61204 |
| 11745433_a_at | 1912 | PHC2 | polyhomeotic homolog 2 (Drosophila) | NM_004427 NM_198040 | 6,45457E-09 | -1,69202 |
| 11759920_at | 80012 | PHC3 | polyhomeotic homolog 3 (Drosophila) | NM_024947 | 4,82295E-06 | -2,14163 |
| 11760107_a_at | 55274 | PHF10 | PHD finger protein 10 | NM_018288 NM_133325 | 6,43996E-13 | -1,75693 |
| 11761326_at | 57649 | PHF12 | PHD finger protein 12 | NM_001033561 NM_020889 | 1,98468E-05 | -2,26005 |
| 11733294_at | 51105 | PHF20L1 | PHD finger protein 20-like 1 | NM_016018 NM_032205 NM_198513 | 1,8723E-09 | -1,84193 |
| 11720361_at | 55023 | PHIP | pleckstrin homology domain interacting protein | NM_017934 | 2,13127E-09 | -1,94303 |
| 11721305_a_at | 22822 | PHLDA1 | pleckstrin homology-like domain, family A, member 1 | NM_007350 | 0,0138547 | -1,87448 |
| 11719250_s_at | 23239 | PHLPP1 | PH domain and leucine rich repeat protein phosphatase 1 | NM_194449 | 8,44443E-07 | -1,50542 |
| 11737589_a_at | 57157 | PHTF2 | putative homeodomain transcription factor 2 | NM_001127357 NM_001127358 NM_001127359 NM_001127360 NM_020432 | 6,87642E-09 | -1,73265 |
| 11731291_a_at | 9063 | PIAS2 | protein inhibitor of activated STAT, 2 | NM_004671 NM_173206 | 1,27925E-11 | -2,08657 |
| 11725842_a_at | 80119 | PIF1 | PIF1 5'-to-3' DNA helicase homolog (S. cerevisiae) | NM_025049 | 0,000184052 | -1,89015 |
| 11725271_a_at | 5277 | PIGA | phosphatidylinositol glycan anchor biosynthesis, class A | NM_002641 NM_020473 | 2,82669E-06 | -1,99682 |
| 11745214_a_at | 5290 | PIK3CA | phosphoinositide-3-kinase, catalytic, alpha polypeptide | NM_006218 | 7,60223E-08 | -1,9889 |
| 11748670_a_at | 5295 | PIK3R1 | phosphoinositide-3-kinase, regulatory subunit 1 (alpha) | NM_181504 NM_181523 NM_181524 | 1,20366E-06 | -2,0777 |
| 11716071_s_at | 415116 | PIM3 | pim-3 oncogene | NM_001001852 | 9,52547E-09 | -2,35609 |
| 11719114_a_at | 9867 | PJA2 | praja ring finger 2 | NM_014819 | 0,0081114 | -1,52064 |
| 11724840_at | 5586 | PKN2 | protein kinase N2 | NM_006256 | 3,2158E-07 | -1,68758 |
| 11738053_x_at | 5329 | PLAUR | plasminogen activator, urokinase receptor | NM_001005376 NM_001005377 NM_002659 | 0,0202227 | -1,92991 |
| 11720296_at | 79666 | PLEKHF2 | pleckstrin homology domain containing, family F (with FYVE domain) member 2 | NM_024613 | 0,000111015 | -1,62546 |
| 11744266_a_at | 23207 | PLEKHM2 | pleckstrin homology domain containing, family M (with RUN domain) member 2 | NM_015164 | 1,61066E-07 | -1,80911 |
| 11742745_a_at | 123 | PLIN2 | perilipin 2 | NM_001122 | 0,0362895 | -1,67937 |
| 11738628_a_at | 50640 | PNPLA8 | patatin-like phospholipase domain containing 8 | NM_015723 | 6,88475E-07 | -1,6803 |
| 11740583_at | 23126 | POGZ | pogo transposable element with ZNF domain | NM_015100 NM_145796 NM_207171 | 1,92959E-06 | -1,54827 |
| 11722451_s_at | 5433 | POLR2D | polymerase (RNA) II (DNA directed) polypeptide D | NM_004805 | 2,0364E-12 | -1,70373 |
| 11730842_a_at | 10621 | POLR3F | polymerase (RNA) III (DNA directed) polypeptide F, 39 kDa | NM_006466 | 3,45855E-10 | -1,66187 |
| 11725975_at | 9883 | POM121 | POM121 membrane glycoprotein (rat) | NM_172020 | 3,96555E-11 | -1,61069 |
| 11726153_at | 5450 | POU2AF1 | POU class 2 associating factor 1 | NM_006235 | 4,5814E-08 | -5,85739 |
| 11720673_a_at | 5494 | PPM1A | protein phosphatase, Mg2+/Mn2+ dependent, 1A | NM_021003 NM_177951 NM_177952 | 8,89827E-05 | -1,7362 |
| 11735019_a_at | 5495 | PPM1B | protein phosphatase, Mg2+/Mn2+ dependent, 1B | NM_001033556 NM_001033557 NM_002706 NM_177968 NM_177969 | 4,49324E-05 | -1,75769 |
| 11739370_a_at | 8493 | PPM1D | protein phosphatase, Mg2+/Mn2+ dependent, 1D | NM_003620 | 5,46636E-07 | -1,85351 |
| 11760681_x_at | 152926 | PPM1K | protein phosphatase, Mg2+/Mn2+ dependent, 1K | NM_152542 | 0,000010024 | -1,54613 |
| 11746720_a_at | 5500 | PPP1CB | protein phosphatase 1, catalytic subunit, beta isozyme | NM_002709 NM_206876 | 6,48585E-05 | -1,8802 |
| 11717661_a_at | 26051 | PPP1R16B | protein phosphatase 1, regulatory (inhibitor) subunit 16B | NM_001172735 NM_015568 | 1,8723E-09 | -3,58713 |
| 11743369_s_at | 5504 | PPP1R2 | protein phosphatase 1, regulatory (inhibitor) subunit 2 | NM_006241 | 0,000121911 | -1,69813 |
| 11721034_at | 79660 | PPP1R3B | protein phosphatase 1, regulatory (inhibitor) subunit 3B | NM_024607 | 0,0001194 | -1,77355 |
| 11746098_a_at | 9989 | PPP4R1 | protein phosphatase 4, regulatory subunit 1 | NM_001042388 NM_005134 | 1,17301E-07 | -1,55339 |
| 11759562_a_at | 151987 | PPP4R2 | protein phosphatase 4, regulatory subunit 2 | NM_174907 | 1,69235E-10 | -1,86398 |
| 11748125_a_at | 5537 | PPP6C | protein phosphatase 6, catalytic subunit | NM_001123355 NM_001123369 NM_002721 | 2,08741E-07 | -1,94559 |
| 11764020_s_at | 160760 | PPTC7 | PTC7 protein phosphatase homolog (S. cerevisiae) | NM_139283 | 5,9172E-09 | -1,74924 |
| 11716358_s_at | 9055 | PRC1 | protein regulator of cytokinesis 1 | NM_003981 NM_199413 NM_199414 | 7,39946E-10 | -2,22558 |
| 11743840_a_at | 7799 | PRDM2 | PR domain containing 2, with ZNF domain | NM_001007257 NM_001135610 NM_012231 NM_015866 | 5,4151E-11 | -2,04925 |
| 11751517_a_at | 5562 | PRKAA1 | protein kinase, AMP-activated, alpha 1 catalytic subunit | NM_006251 NM_206907 | 2,91823E-05 | -1,851 |
| 11753791_s_at | 5573 | PRKAR1A | protein kinase, cAMP-dependent, regulatory, type I, alpha (tissue specific extinguisher | NM_002734 NM_212471 NM_212472 | 2,37739E-09 | -3,35559 |
| 11745482_s_at | 5621 | PRNP | prion protein | NM_000311 NM_001080121 NM_001080122 NM_001080123 NM_183079 | 8,75189E-09 | -2,37476 |
| 11743384_at | 55660 | PRPF40A | PRP40 pre-mRNA processing factor 40 homolog A (S. cerevisiae) | NM_017892 | 9,872E-11 | -1,82155 |
| 11718235_at | 8899 | PRPF4B | PRP4 pre-mRNA processing factor 4 homolog B (yeast) | NM_003913 | 8,78752E-09 | -1,78317 |
| 11734600_a_at | 11168 | PSIP1 | PC4 and SFRS1 interacting protein 1 | NM_001128217 NM_021144 NM_033222 | 0,000390951 | -1,54682 |
| 11760100_x_at | 5690 | PSMB2 | proteasome (prosome, macropain) subunit, beta type, 2 | NM_002794 | 1,55837E-07 | -1,60564 |
| 11760946_at | 5701 | PSMC2 | Proteasome (prosome, macropain) 26S subunit, ATPase, 2 | NM_002803 | 2,27697E-05 | -1,56214 |
| 11746059_a_at | 5713 | PSMD7 | proteasome (prosome, macropain) 26S subunit, non-ATPase, 7 | NM_002811 | 3,0953E-08 | -1,72868 |
| 11755120_a_at | 23198 | PSME4 | proteasome (prosome, macropain) activator subunit 4 | NM_014614 | 1,27925E-11 | -2,14653 |
| 11725028_s_at | 55269 | PSPC1 | paraspeckle component 1 | NM_001042414 NR_003272 | 4,73118E-10 | -1,57162 |
| 11742677_x_at | 5725 | PTBP1 | polypyrimidine tract binding protein 1 | NM_002819 NM_031990 NM_031991 NM_175847 | 2,13546E-06 | -1,91027 |
| 11722804_x_at | 58155 | PTBP2 | polypyrimidine tract binding protein 2 | NM_021190 | 2,26789E-08 | -2,04507 |
| 11758421_s_at | 5728 | PTEN | phosphatase and tensin homolog | NM_000314 | 2,41997E-07 | -1,59627 |
| 11717897_a_at | 7803 | PTP4A1 | protein tyrosine phosphatase type IVA, member 1 | NM_003463 | 0,00106971 | -2,02158 |
| 11726806_s_at | 114971 | PTPMT1 | protein tyrosine phosphatase, mitochondrial 1 | NM_001143984 NM_175732 | 8,14917E-09 | -1,72142 |
| 11743596_a_at | 5791 | PTPRE | protein tyrosine phosphatase, receptor type, E | NM_006504 NM_130435 | 4,252E-08 | -3,11763 |
| 11748996_a_at | 23369 | PUM2 | pumilio homolog 2 (Drosophila) | NM_015317 | 1,56856E-07 | -1,78646 |
| 11716923_a_at | 5814 | PURB | purine-rich element binding protein B | NM_033224 | 8,21947E-06 | -2,53453 |
| 11719528_at | 5819 | PVRL2 | poliovirus receptor-related 2 (herpesvirus entry mediator B) | NM_001042724 NM_002856 | 0,000179494 | -1,74296 |
| 11736783_s_at | 80223 | RAB11FIP1 | RAB11 family interacting protein 1 (class I) | NM_001002233 NM_001002814 NM_025151 | 1,14806E-07 | -2,92758 |
| 11724143_s_at | 22841 | RAB11FIP2 | RAB11 family interacting protein 2 (class I) | NM_014904 | 4,45135E-09 | -1,77973 |
| 11720393_at | 84440 | RAB11FIP4 | RAB11 family interacting protein 4 (class II) | NM_032932 | 0,000080336 | -1,60695 |
| 11759678_at | 201475 | RAB12 | RAB12, member RAS oncogene family | NM_001025300 | 7,50707E-08 | -1,84849 |
| 11751040_a_at | 22931 | RAB18 | RAB18, member RAS oncogene family | NM_021252 | 7,58967E-05 | -1,67435 |
| 11727505_at | 23011 | RAB21 | RAB21, member RAS oncogene family | NM_014999 | 1,24822E-09 | -2,17499 |
| 11718218_at | 57403 | RAB22A | RAB22A, member RAS oncogene family | NM_020673 | 4,71624E-14 | -1,97886 |
| 11750261_a_at | 22930 | RAB3GAP1 | RAB3 GTPase activating protein subunit 1 (catalytic) | NM_001172435 NM_012233 | 1,13538E-10 | -1,81673 |
| 11757590_s_at | 339122 | RAB43 | RAB43, member RAS oncogene family | NM_198490 | 1,83397E-05 | -1,59986 |
| 11722789_a_at | 5868 | RAB5A | RAB5A, member RAS oncogene family | NM_004162 | 0,00136428 | -1,67924 |
| 11720798_at | 51762 | RAB8B | RAB8B, member RAS oncogene family | NM_016530 | 0,00170462 | -1,61655 |
| 11748733_a_at | 27342 | RABGEF1 | RAB guanine nucleotide exchange factor (GEF) 1 | NM_014504 | 1,74862E-07 | -2,50971 |
| 11731506_a_at | 5887 | RAD23B | RAD23 homolog B (S. cerevisiae) | NM_002874 | 8,23995E-08 | -1,88169 |
| 11748273_a_at | 5894 | RAF1 | v-raf-1 murine leukemia viral oncogene homolog 1 | NM_002880 | 4,0496E-06 | -1,55413 |
| 11737830_at | 5896 | RAG1 | recombination activating gene 1 | NM_000448 | 5,93905E-06 | -4,54523 |
| 11748706_a_at | 22913 | RALY | RNA binding protein, autoantigenic (hnRNP-associated with lethal yellow homolog (mouse) | NM_007367 NM_016732 | 6,69923E-06 | -1,9033 |
| 11722816_at | 5903 | RANBP2 | RAN binding protein 2 | NM_006267 | 4,29601E-10 | -1,88027 |
| 11757841_s_at | 10048 | RANBP9 | RAN binding protein 9 | NM_005493 | 1,79248E-10 | -1,58512 |
| 11716435_a_at | 2889 | RAPGEF1 | Rap guanine nucleotide exchange factor (GEF) 1 | NM_005312 NM_198679 | 3,48929E-07 | -1,86394 |
| 11743480_s_at | 9821 | RB1CC1 | RB1-inducible coiled-coil 1 | NM_001083617 NM_014781 | 5,74996E-08 | -1,9654 |
| 11730916_a_at | 5930 | RBBP6 | retinoblastoma binding protein 6 | NM_006910 NM_018703 NM_032626 | 8,96564E-05 | -1,84658 |
| 11719925_a_at | 10137 | RBM12 | RNA binding motif protein 12 | NM_006047 NM_152838 | 9,54709E-07 | -1,53 |
| 11749638_a_at | 22828 | RBM16 | RNA binding motif protein 16 | NM_014892 | 3,17754E-08 | -1,61712 |
| 11755224_a_at | 155435 | RBM33 | RNA binding motif protein 33 | NM_053043 | 2,7714E-08 | -1,50813 |
| 11715594_a_at | 55544 | RBM38 | RNA binding motif protein 38 | NM_017495 NM_183425 | 2,80035E-06 | -1,87755 |
| 11761738_x_at | 27316 | RBMX | RNA binding motif protein, X-linked | NM_001164803 NM_002139 NR_028476 NR_028477 | 3,95056E-08 | -1,68962 |
| 11726095_at | 494115 | RBMXL1 | RNA binding motif protein, X-linked-like 1 | NM_001162536 NM_019610 | 1,05687E-07 | -1,71281 |
| 11764053_a_at | 3516 | RBPJ | recombination signal binding protein for immunoglobulin kappa J region | NM_005349 NM_015874 NM_203283 NM_203284 | 0,000261034 | -1,63406 |
| 11737961_s_at | 55758 | RCOR3 | REST corepressor 3 | NM_001136223 NM_001136224 NM_001136225 NM_018254 | 4,40012E-07 | -1,6158 |
| 11726839_s_at | 768211 | RELL1 | RELT-like 1 | NM_001085399 NM_001085400 | 8,86321E-12 | -2,13739 |
| 11739558_at | 55159 | RFWD3 | ring finger and WD repeat domain 3 | NM_018124 | 1,62961E-07 | -1,73273 |
| 11716553_a_at | 57414 | RHBDD2 | rhomboid domain containing 2 | NM_001040456 NM_001040457 | 3,04307E-08 | -1,57087 |
| 11754028_s_at | 6009 | RHEB | Ras homolog enriched in brain | NM_005614 | 9,89525E-05 | -1,57437 |
| 11758676_s_at | 23433 | RHOQ | ras homolog gene family, member Q | NM_012249 | 4,84137E-06 | -1,66208 |
| 11758472_s_at | 253260 | RICTOR | RPTOR independent companion of MTOR, complex 2 | NM_152756 | 1,32608E-06 | -2,1197 |
| 11739663_at | 9783 | RIMS3 | regulating synaptic membrane exocytosis 3 | NM_014747 | 1,59045E-06 | -2,27647 |
| 11725077_at | 6016 | RIT1 | Ras-like without CAAX 1 | NM_006912 | 0,000831325 | -2,221 |
| 11758122_s_at | 6018 | RLF | rearranged L-myc fusion | NM_012421 | 7,85982E-08 | -3,27217 |
| 11734799_s_at | 51132 | RLIM | ring finger protein, LIM domain interacting | NM_016120 NM_183353 | 0,000365894 | -1,62956 |
| 11743344_a_at | 64795 | RMND5A | required for meiotic nuclear division 5 homolog A (S. cerevisiae) | NM_022780 | 0,00106772 | -2,00638 |
| 11725952_at | 9921 | RNF10 | ring finger protein 10 | NM_014868 | 6,61967E-05 | -1,94312 |
| 11717807_at | 7844 | RNF103 | ring finger protein 103 | NM_005667 | 2,74746E-07 | -1,92761 |
| 11715622_a_at | 55905 | RNF114 | ring finger protein 114 | NM_018683 | 1,34173E-06 | -2,1644 |
| 11758333_s_at | 379013 51444 | RNF138 RNF138P1 | ring finger protein 138 ring finger protein 138 pseudogene 1 | NM_016271 NM_198128 NR_001575 | 5,50593E-06 | -2,53657 |
| 11745856_x_at | 153830 | RNF145 | ring finger protein 145 | NM_144726 | 5,41179E-06 | -2,79636 |
| 11717689_a_at | 25897 | RNF19A | ring finger protein 19A | NM_015435 NM_183419 | 0,00267873 | -1,52784 |
| 11718095_s_at | 152006 | RNF38 | ring finger protein 38 | NM_022781 NM_194328 NM_194329 NM_194330 NM_194332 | 4,59359E-07 | -1,78032 |
| 11721954_s_at | 10193 | RNF41 | ring finger protein 41 | NM_005785 NM_194358 NM_194359 | 3,95356E-11 | -1,70618 |
| 11716756_at | 9616 | RNF7 | ring finger protein 7 | NM_014245 NM_183237 | 1,03884E-06 | -2,33417 |
| 11728296_a_at | 8731 | RNMT | RNA (guanine-7-) methyltransferase | NM_003799 | 8,57444E-09 | -3,29003 |
| 11739631_at | 6103 | RPGR | retinitis pigmentosa GTPase regulator | NM_000328 NM_001034853 | 2,32648E-07 | -1,60765 |
| 11757122_s_at | 6161 | RPL32 | ribosomal protein L32 | NM_000994 NM_001007073 NM_001007074 | 2,05035E-07 | -1,77686 |
| 11728348_a_at | 116832 | RPL39L | ribosomal protein L39-like | NM_052969 | 0,0132216 | -1,6374 |
| 11749241_a_at | 11102 | RPP14 | ribonuclease P/MRP 14kDa subunit | NM_001098783 NM_007042 | 1,02776E-08 | -1,57609 |
| 11733865_at | 10556 | RPP30 | ribonuclease P/MRP 30kDa subunit | NM_001104546 NM_006413 | 2,12449E-09 | -1,78682 |
| 11718279_at | 58490 | RPRD1B | regulation of nuclear pre-mRNA domain containing 1B | NM_021215 | 7,40957E-11 | -1,89234 |
| 11747836_a_at | 6198 | RPS6KB1 | ribosomal protein S6 kinase, 70kDa, polypeptide 1 | NM_003161 | 5,72494E-07 | -1,58715 |
| 11724550_at | 54665 | RSBN1 | round spermatid basic protein 1 | NM_018364 | 6,20414E-06 | -1,58715 |
| 11743274_x_at | 65117 | RSRC2 | arginine/serine-rich coiled-coil 2 | NM_023012 NM_198261 NM_198262 | 9,27309E-07 | -1,55988 |
| 11717357_at | 23168 | RTF1 | Rtf1, Paf1/RNA polymerase II complex component, homolog (S. cerevisiae) | NM_015138 | 6,27095E-11 | -1,77578 |
| 11738035_s_at | 57142 | RTN4 | reticulon 4 | NM_007008 NM_020532 NM_153828 NM_207520 NM_207521 | 0,000021916 | -2,17406 |
| 11743533_x_at | 22902 | RUFY3 | RUN and FYVE domain containing 3 | NM_001037442 NM_001130709 NM_014961 | 6,93538E-07 | -1,52416 |
| 11725180_a_at | 860 | RUNX2 | runt-related transcription factor 2 | NM_001015051 NM_001024630 NM_004348 | 1,00415E-06 | -2,10416 |
| 11717538_a_at | 23429 | RYBP | RING1 and YY1 binding protein | NM_012234 | 3,83642E-08 | -1,83349 |
| 11726241_at | 6263 | RYR3 | ryanodine receptor 3 | NM_001036 | 0,00241489 | -1,73407 |
| 11743816_s_at | 1901 | S1PR1 | sphingosine-1-phosphate receptor 1 | NM_001400 | 0,00967246 | -2,28665 |
| 11741306_a_at | 142891 | SAMD8 | sterile alpha motif domain containing 8 | NM_001174156 NM_144660 | 1,54103E-05 | -1,96259 |
| 11744403_x_at | 29115 | SAP30BP | SAP30 binding protein | NM_013260 | 3,11422E-08 | -1,70471 |
| 11717130_a_at | 56681 | SAR1A | SAR1 homolog A (S. cerevisiae) | NM_001142648 NM_020150 | 8,55508E-05 | -1,71187 |
| 11760027_at | 9733 | SART3 | squamous cell carcinoma antigen recognized by T cells 3 | NM_014706 | 2,33316E-06 | -1,97764 |
| 11742794_s_at | 155370 51119 | SBDS SBDSP1 | Shwachman-Bodian-Diamond syndrome Shwachman-Bodian-Diamond syndrome pseudogene 1 | NM_016038 NR_001588 NR_024109 NR_024110 NR_024111 | 0,000276578 | -1,65677 |
| 11732841_a_at | 6328 | SCN3A | sodium channel, voltage-gated, type III, alpha subunit | NM_001081676 NM_001081677 NM_006922 | 2,05526E-09 | -2,74159 |
| 11739335_at | 55681 | SCYL2 | SCY1-like 2 (S. cerevisiae) | NM_017988 | 2,13546E-06 | -1,57582 |
| 11749096_a_at | 9147 | SDCCAG1 | serologically defined colon cancer antigen 1 | NM_004713 | 1,39201E-10 | -1,57674 |
| 11729958_at | 54549 | SDK2 | sidekick homolog 2 (chicken) | NM_001144952 | 8,23868E-08 | -2,32351 |
| 11743926_a_at | 10802 | SEC24A | SEC24 family, member A (S. cerevisiae) | NM_021982 | 0,000558407 | -1,92533 |
| 11718863_at | 11231 | SEC63 | SEC63 homolog (S. cerevisiae) | NM_007214 | 4,29065E-15 | -1,62789 |
| 11717600_a_at | 79048 | SECISBP2 | SECIS binding protein 2 | NM_024077 | 1,43433E-07 | -1,55983 |
| 11744132_at | 9728 | SECISBP2L | SECIS binding protein 2-like | NM_014701 | 1,36185E-05 | -1,72715 |
| 11720485_s_at | 59343 | SENP2 | SUMO1/sentrin/SMT3 specific peptidase 2 | NM_021627 | 3,09226E-09 | -1,69582 |
| 11739942_s_at | 51091 | SEPSECS | Sep (O-phosphoserine) tRNA:Sec (selenocysteine) tRNA synthase | NM_016955 NM_153825 | 1,50681E-07 | -1,53298 |
| 11744594_a_at | 23157 | SEPT6 | septin 6 | NM_015129 NM_145799 NM_145800 NM_145802 | 0,00108677 | -1,55497 |
| 11716631_s_at | 5055 | SERPINB2 | serpin peptidase inhibitor, clade B (ovalbumin), member 2 | NM_001143818 NM_002575 | 0,0249048 | -1,91717 |
| 11728413_at | 5271 | SERPINB8 | serpin peptidase inhibitor, clade B (ovalbumin), member 8 | NM_001031848 NM_002640 NM_198833 | 4,73921E-07 | -2,3712 |
| 11733919_s_at | 9792 | SERTAD2 | SERTA domain containing 2 | NM_014755 | 2,01424E-06 | -1,71191 |
| 11719062_a_at | 27244 | SESN1 | sestrin 1 | NM_014454 | 0,000393463 | -1,85142 |
| 11716192_a_at | 29072 | SETD2 | SET domain containing 2 | NM_014159 | 1,94279E-12 | -1,7833 |
| 11717555_at | 80854 | SETD7 | SET domain containing (lysine methyltransferase) 7 | NM_030648 | 3,06701E-10 | -2,05752 |
| 11748745_a_at | 7536 | SF1 | splicing factor 1 | NM_001178030 NM_001178031 NM_004630 NM_201995 NM_201997 NM_201998 / | 1,1176E-12 | -2,12954 |
| 11720474_a_at | 6421 | SFPQ | splicing factor proline/glutamine-rich | NM_005066 | 9,01621E-13 | -1,58549 |
| 11761781_a_at | 9295 | SFRS11 | splicing factor, arginine/serine-rich 11 | NM_004768 | 2,68213E-05 | -1,75831 |
| 11739377_x_at | 140890 | SFRS12 | splicing factor, arginine/serine-rich 12 | NM_001077199 NM_139168 | 5,31554E-07 | -1,80408 |
| 11721310_s_at | 10772 | SFRS13A | splicing factor, arginine/serine-rich 13A | NM_006625 NM_054016 XM_001713958 XM_001713979 XM_002344446 XM_00234 | 7,64593E-12 | -1,78398 |
| 11717409_at | 10019 | SH2B3 | SH2B adaptor protein 3 | NM_005475 | 2,12371E-08 | -1,81301 |
| 11754466_a_at | 23677 | SH3BP4 | SH3-domain binding protein 4 | NM_014521 | 0,00578289 | -1,5102 |
| 11724483_s_at | 8036 | SHOC2 | soc-2 suppressor of clear homolog (C. elegans) | NM_007373 | 4,91262E-10 | -1,51818 |
| 11752177_a_at | 6477 | SIAH1 | seven in absentia homolog 1 (Drosophila) | NM_001006610 NM_003031 | 3,85522E-06 | -2,2558 |
| 11743067_at | 6478 | SIAH2 | seven in absentia homolog 2 (Drosophila) | NM_005067 | 3,33858E-11 | -2,33512 |
| 11716771_s_at | 150094 | SIK1 | salt-inducible kinase 1 | NM_173354 | 2,98576E-09 | -4,37874 |
| 11730294_a_at | 25942 | SIN3A | SIN3 homolog A, transcription regulator (yeast) | NM_001145357 NM_001145358 NM_015477 | 3,45071E-15 | -1,66471 |
| 11763339_a_at | 10572 | SIVA1 | SIVA1, apoptosis-inducing factor | NM_006427 NM_021709 | 2,94529E-10 | -1,60028 |
| 11741553_a_at | 6498 | SKIL | SKI-like oncogene | NM_001145097 NM_001145098 NM_005414 | 2,76837E-07 | -3,86797 |
| 11722823_a_at | 6500 | SKP1 | S-phase kinase-associated protein 1 | NM_006930 NM_170679 | 1,59185E-06 | -1,89419 |
| 11746556_a_at | 7884 | SLBP | stem-loop binding protein | NM_006527 | 1,22517E-06 | -1,57078 |
| 11744052_a_at | 4891 | SLC11A2 | solute carrier family 11 (proton-coupled divalent metal ion transporters), member 2 | NM_000617 NM_001174125 NM_001174126 NM_001174127 NM_001174128 NM_00 | 2,50618E-07 | -1,56871 |
| 11724324_a_at | 9990 | SLC12A6 | solute carrier family 12 (potassium/chloride transporters), member 6 | NM_001042494 NM_001042495 NM_001042496 NM_001042497 NM_005135 NM_13 | 0,00954123 | -1,56602 |
| 11725662_a_at | 151473 | SLC16A14 | solute carrier family 16, member 14 (monocarboxylic acid transporter 14) | NM_152527 | 0,00196146 | -1,68674 |
| 11723477_x_at | 26503 | SLC17A5 | solute carrier family 17 (anion/sugar transporter), member 5 | NM_012434 | 0,000101979 | -1,71259 |
| 11744616_a_at | 10560 | SLC19A2 | solute carrier family 19 (thiamine transporter), member 2 | NM_006996 | 1,0361E-06 | -1,87194 |
| 11724454_at | 6583 | SLC22A4 | solute carrier family 22 (organic cation/ergothioneine transporter), member 4 | NM_003059 | 0,000246335 | -1,75829 |
| 11719859_a_at | 56731 | SLC2A4RG | SLC2A4 regulator | NM_020062 | 9,04782E-07 | -1,57216 |
| 11726006_at | 7779 | SLC30A1 | solute carrier family 30 (zinc transporter), member 1 | NM_021194 | 2,93434E-08 | -4,17791 |
| 11759004_at | 9197 | SLC33A1 | solute carrier family 33 (acetyl-CoA transporter), member 1 | NM_004733 | 6,20603E-07 | -1,70632 |
| 11736468_at | 23169 | SLC35D1 | solute carrier family 35 (UDP-glucuronic acid/UDP-N-acetylgalactosamine dual transporte | NM_015139 | 1,22291E-10 | -1,89219 |
| 11725219_a_at | 9748 | SLK | STE20-like kinase (yeast) | NM_014720 | 5,9159E-08 | -1,63572 |
| 11761874_at | 7871 | SLMAP | sarcolemma associated protein | NM_007159 | 0,000223934 | -1,70003 |
| 11748335_a_at | 51012 | SLMO2 | slowmo homolog 2 (Drosophila) | NM_016045 | 0,000025786 | -1,61865 |
| 11724469_at | 10569 | SLU7 | SLU7 splicing factor homolog (S. cerevisiae) | NM_006425 | 5,34321E-06 | -1,53843 |
| 11747501_a_at | 4086 | SMAD1 | SMAD family member 1 | NM_001003688 NM_005900 | 0,000294055 | -1,86318 |
| 11730338_a_at | 4091 | SMAD6 | SMAD family member 6 | NM_001142861 NM_005585 NR_027654 | 0,0020394 | -1,88983 |
| 11725514_a_at | 4092 | SMAD7 | SMAD family member 7 | NM_005904 | 2,94529E-10 | -7,45585 |
| 11761728_a_at | 6605 | SMARCE1 | SWI/SNF related, matrix associated, actin dependent regulator of chromatin, subfamily e | NM_003079 | 1,30833E-05 | -1,83638 |
| 11720585_a_at | 23347 | SMCHD1 | structural maintenance of chromosomes flexible hinge domain containing 1 | NM_015295 | 1,15411E-10 | -1,76532 |
| 11719758_a_at | 140775 | SMCR8 | Smith-Magenis syndrome chromosome region, candidate 8 | NM_144775 | 1,39515E-07 | -1,52808 |
| 11762978_x_at | 6606 | SMN1 | survival of motor neuron 1, telomeric | NM_000344 NM_022874 | 3,63971E-07 | -1,69677 |
| 11722957_a_at | 57154 | SMURF1 | SMAD specific E3 ubiquitin protein ligase 1 | NM_020429 NM_181349 | 1,07057E-09 | -2,31882 |
| 11743762_at | 79753 | SNIP1 | Smad nuclear interacting protein 1 | NM_024700 | 5,10452E-11 | -2,33975 |
| 11719046_a_at | 54861 | SNRK | SNF related kinase | NM_001100594 NM_017719 | 3,64379E-07 | -1,64004 |
| 11755612_s_at | 6627 | SNRPA1 | small nuclear ribonucleoprotein polypeptide A' | NM_003090 | 1,07395E-22 | -1,82896 |
| 11754272_x_at | 6638 8926 | SNRPN SNURF | small nuclear ribonucleoprotein polypeptide N SNRPN upstream reading frame | NM_003097 NM_005678 NM_022804 NM_022805 NM_022806 NM_022807 NM_ | 0,00123981 | -1,50688 |
| 11756208_a_at | 51429 | SNX9 | sorting nexin 9 | NM_016224 | 5,50384E-06 | -2,14148 |
| 11739980_a_at | 122809 | SOCS4 | suppressor of cytokine signaling 4 | NM_080867 NM_199421 | 7,22534E-08 | -1,91467 |
| 11758088_s_at | 9306 | SOCS6 | suppressor of cytokine signaling 6 | NM_004232 | 6,5438E-09 | -1,66535 |
| 11724655_at | 6651 | SON | SON DNA binding protein | NM_032195 NM_138927 | 3,47396E-08 | -2,0765 |
| 11758883_at | 6670 | SP3 | Sp3 transcription factor | NM_001017371 NM_001172712 NM_003111 | 9,33996E-08 | -1,96807 |
| 11744885_a_at | 9043 | SPAG9 | sperm associated antigen 9 | NM_001130528 NM_003971 | 4,71486E-10 | -1,88573 |
| 11721117_at | 60559 | SPCS3 | signal peptidase complex subunit 3 homolog (S. cerevisiae) | NM_021928 | 1,13403E-07 | -1,58127 |
| 11743666_at | 23013 | SPEN | spen homolog, transcriptional regulator (Drosophila) | NM_015001 | 2,05795E-12 | -1,76742 |
| 11746362_x_at | 201305 | SPNS3 | spinster homolog 3 (Drosophila) | NM_182538 | 0,000272379 | -1,63863 |
| 11724503_at | 10418 | SPON1 | spondin 1, extracellular matrix protein | NM_006108 | 4,04289E-05 | -1,50987 |
| 11724643_at | 339745 | SPOPL | speckle-type POZ protein-like | NM_001001664 | 0,0014179 | -1,87583 |
| 11717652_s_at | 10252 | SPRY1 | sprouty homolog 1, antagonist of FGF signaling (Drosophila) | NM_005841 NM_199327 | 0,000172658 | -1,59515 |
| 11722690_at | 6711 | SPTBN1 | spectrin, beta, non-erythrocytic 1 | NM_003128 NM_178313 | 1,35129E-08 | -5,93132 |
| 11731838_at | 10558 | SPTLC1 | serine palmitoyltransferase, long chain base subunit 1 | NM_006415 NM_178324 | 1,00886E-06 | -1,75156 |
| 11756771_x_at | 144108 | SPTY2D1 | SPT2, Suppressor of Ty, domain containing 1 (S. cerevisiae) | NM_194285 | 4,507E-09 | -2,11921 |
| 11752843_x_at | 8878 | SQSTM1 | sequestosome 1 | NM_001142298 NM_001142299 NM_003900 | 0,00264173 | -1,58278 |
| 11758864_at | 6728 | SRP19 | signal recognition particle 19kDa | NM_003135 | 2,83075E-08 | -1,83477 |
| 11750541_a_at | 6744 | SSFA2 | sperm specific antigen 2 | NM_001130445 NM_006751 | 4,57126E-05 | -1,66095 |
| 11744850_a_at | 85464 | SSH2 | slingshot homolog 2 (Drosophila) | NM_033389 | 0,0133403 | -1,55426 |
| 11743839_a_at | 10402 | ST3GAL6 | ST3 beta-galactoside alpha-2,3-sialyltransferase 6 | NM_006100 | 1,65292E-06 | -2,11249 |
| 11717904_a_at | 6780 | STAU1 | staufen, RNA binding protein, homolog 1 (Drosophila) | NM_001037328 NM_004602 NM_017452 NM_017453 NM_017454 | 8,84486E-06 | -1,55174 |
| 11722655_s_at | 9263 | STK17A | serine/threonine kinase 17a | NM_004760 | 0,000071846 | -2,07683 |
| 11726042_a_at | 9262 | STK17B | serine/threonine kinase 17b | NM_004226 | 0,000134544 | -2,6027 |
| 11724714_a_at | 55351 | STK32B | serine/threonine kinase 32B | NM_018401 | 1,47817E-05 | -1,62583 |
| 11738538_x_at | 3925 | STMN1 | stathmin 1 | NM_001145454 NM_005563 NM_203399 NM_203401 | 2,3565E-12 | -1,68941 |
| 11751052_a_at | 11171 | STRAP | serine/threonine kinase receptor associated protein | NM_007178 | 2,35022E-08 | -1,57137 |
| 11721860_s_at | 23673 | STX12 | syntaxin 12 | NM_177424 | 1,24236E-07 | -2,21584 |
| 11751297_s_at | 10923 | SUB1 | SUB1 homolog (S. cerevisiae) | NM_006713 | 6,97939E-09 | -2,13608 |
| 11750021_x_at | 6836 | SURF4 | surfeit 4 | NM_033161 | 8,14715E-08 | -1,61791 |
| 11721500_at | 23075 | SWAP70 | SWAP switching B-cell complex 70kDa subunit | NM_015055 | 7,54327E-06 | -1,59918 |
| 11744427_a_at | 94056 | SYAP1 | synapse associated protein 1, SAP47 homolog (Drosophila) | NM_032796 NR_033181 | 1,16454E-11 | -2,49969 |
| 11739102_a_at | 25949 | SYF2 | SYF2 homolog, RNA splicing factor (S. cerevisiae) | NM_015484 NM_207170 | 2,89397E-08 | -2,76817 |
| 11748695_a_at | 9145 | SYNGR1 | synaptogyrin 1 | NM_004711 NM_145731 NM_145738 | 1,83006E-06 | -1,60242 |
| 11748535_x_at | 6882 | TAF11 | TAF11 RNA polymerase II, TATA box binding protein (TBP)-associated factor, 28kDa | NM_005643 | 5,06713E-09 | -1,70877 |
| 11736606_a_at | 27097 | TAF5L | TAF5-like RNA polymerase II, p300/CBP-associated factor (PCAF)-associated factor, 65kDa | NM_001025247 NM_014409 | 1,35321E-08 | -1,66574 |
| 11753782_a_at | 10010 | TANK | TRAF family member-associated NFKB activator | NM_004180 NM_133484 | 1,24036E-05 | -1,98533 |
| 11723832_a_at | 202018 | TAPT1 | transmembrane anterior posterior transformation 1 | NM_153365 | 4,96152E-11 | -4,34751 |
| 11727377_a_at | 64786 | TBC1D15 | TBC1 domain family, member 15 | NM_001146213 NM_001146214 NM_022771 NR_027449 | 2,38858E-07 | -1,60146 |
| 11754170_a_at | 84897 | TBRG1 | transforming growth factor beta regulator 1 | NM_032811 NR_016021 | 1,53177E-06 | -1,79909 |
| 11727495_at | 6924 | TCEB3 | transcription elongation factor B (SIII), polypeptide 3 (110kDa, elongin A) | NM_003198 | 6,90547E-13 | -1,58761 |
| 11755059_s_at | 6929 | TCF3 | transcription factor 3 (E2A immunoglobulin enhancer binding factors E12/E47) | NM_001136139 NM_003200 | 1,1165E-07 | -1,61282 |
| 11754733_a_at | 6925 | TCF4 | transcription factor 4 | NM_001083962 NM_003199 | 1,39873E-07 | -2,3919 |
| 11737662_a_at | 54790 | TET2 | tet oncogene family member 2 | NM_001127208 NM_017628 | 0,00623934 | -1,92756 |
| 11740938_a_at | 7050 | TGIF1 | TGFB-induced factor homeobox 1 | NM_003244 NM_170695 NM_173207 NM_173208 NM_173209 NM_173210 NM_ | 3,7837E-07 | -1,76005 |
| 11755219_a_at | 7056 | THBD | thrombomodulin | NM_000361 | 0,0430812 | -1,51168 |
| 11748096_a_at | 25917 | THUMPD3 | THUMP domain containing 3 | NM_001114092 NM_015453 | 1,74148E-08 | -1,51528 |
| 11748156_a_at | 7072 | TIA1 | TIA1 cytotoxic granule-associated RNA binding protein | NM_022037 NM_022173 | 1,25208E-07 | -2,71261 |
| 11720681_at | 92610 | TIFA | TRAF-interacting protein with forkhead-associated domain | NM_052864 | 5,36272E-08 | -1,7499 |
| 11719995_a_at | 7091 | TLE4 | transducin-like enhancer of split 4 (E(sp1) homolog, Drosophila) | NM_007005 | 1,66034E-15 | -2,23029 |
| 11744472_a_at | 11011 | TLK2 | tousled-like kinase 2 | NM_001112707 NM_006852 | 2,634E-10 | -1,52236 |
| 11716947_at | 56889 | TM9SF3 | transmembrane 9 superfamily member 3 | NM_020123 | 1,27606E-09 | -2,05339 |
| 11715544_a_at | 10972 | TMED10 | transmembrane emp24-like trafficking protein 10 (yeast) | NM_006827 | 5,34019E-11 | -1,90097 |
| 11716229_s_at | 56900 | TMEM167B | transmembrane protein 167B | NM_020141 | 4,38605E-05 | -1,56811 |
| 11758891_at | 92703 | TMEM183A | transmembrane protein 183A | NM_138391 | 3,09878E-08 | -1,8309 |
| 11732201_a_at | 55254 | TMEM39A | transmembrane protein 39A | NM_018266 | 2,70134E-11 | -2,15522 |
| 11726381_a_at | 440026 | TMEM41B | transmembrane protein 41B | NM_001165030 NM_015012 NR_028491 | 8,52924E-06 | -2,52078 |
| 11731547_a_at | 91147 | TMEM67 | transmembrane protein 67 | NM_001142301 NM_153704 NR_024522 | 4,00835E-07 | -1,6709 |
| 11724753_at | 7110 | TMF1 | TATA element modulatory factor 1 | NM_007114 | 1,71338E-06 | -1,71602 |
| 11718939_s_at | 7128 | TNFAIP3 | tumor necrosis factor, alpha-induced protein 3 | NM_006290 | 0,0309619 | -1,59341 |
| 11720972_at | 7153 | TOP2A | topoisomerase (DNA) II alpha 170kDa | NM_001067 | 0,0185304 | -1,96367 |
| 11741099_a_at | 7159 | TP53BP2 | tumor protein p53 binding protein, 2 | NM_001031685 NM_005426 | 2,76825E-10 | -1,7776 |
| 11718610_at | 94241 | TP53INP1 | tumor protein p53 inducible nuclear protein 1 | NM_001135733 NM_033285 | 6,73323E-10 | -2,51897 |
| 11716399_at | 58476 | TP53INP2 | tumor protein p53 inducible nuclear protein 2 | NM_021202 | 0,000199656 | -1,5455 |
| 11731455_at | 7170 | TPM3 | tropomyosin 3 | NM_001043351 NM_001043352 NM_001043353 NM_152263 NM_153649 | 1,83267E-08 | -2,46117 |
| 11743501_a_at | 7187 | TRAF3 | TNF receptor-associated factor 3 | NM_003300 NM_145725 NM_145726 | 2,07491E-06 | -1,76473 |
| 11747523_a_at | 9618 | TRAF4 | TNF receptor-associated factor 4 | NM_004295 | 1,38331E-05 | -1,66182 |
| 11720285_s_at | 22906 | TRAK1 | trafficking protein, kinesin binding 1 | NM_001042646 NM_014965 | 3,37877E-10 | -1,71399 |
| 11746721_a_at | 54210 | TREM1 | triggering receptor expressed on myeloid cells 1 | NM_018643 | 0,0239323 | -1,72831 |
| 11739282_a_at | 373 | TRIM23 | tripartite motif-containing 23 | NM_001656 NM_033227 NM_033228 | 0,000109046 | -1,78834 |
| 11720261_a_at | 51592 | TRIM33 | tripartite motif-containing 33 | NM_015906 NM_033020 | 1,30958E-08 | -1,6917 |
| 11733897_a_at | 6738 | TROVE2 | TROVE domain family, member 2 | NM_001042369 NM_001042370 NM_001173524 NM_001173525 NM_004600 NR_03 | 7,13595E-07 | -2,24185 |
| 11752636_a_at | 26133 | TRPC4AP | transient receptor potential cation channel, subfamily C, member 4 associated protein | NM_015638 NM_199368 | 2,18488E-10 | -1,63898 |
| 11735128_a_at | 9819 | TSC22D2 | TSC22 domain family, member 2 | NM_014779 | 2,27873E-06 | -1,93566 |
| 11718512_x_at | 81619 | TSPAN14 | tetraspanin 14 | NM_001128309 NM_030927 | 3,70459E-08 | -1,80086 |
| 11757628_s_at | 64061 | TSPYL2 | TSPY-like 2 | NM_022117 | 0,00102671 | -1,82432 |
| 11754362_a_at | 57217 | TTC7A | tetratricopeptide repeat domain 7A | NM_020458 | 4,21558E-08 | -1,69761 |
| 11733881_at | 7270 | TTF1 | transcription termination factor, RNA polymerase I | NM_007344 | 9,02573E-09 | -1,58659 |
| 11752922_x_at | 203068 | TUBB | tubulin, beta | NM_178014 | 6,3335E-07 | -1,8931 |
| 11719788_s_at | 7280 | TUBB2A | tubulin, beta 2A | NM_001069 | 0,00730679 | -2,02812 |
| 11724882_x_at | 7286 | TUFT1 | tuftelin 1 | NM_001126337 NM_020127 | 0,00421483 | -1,59142 |
| 11716945_s_at | 5756 | TWF1 | twinfilin, actin-binding protein, homolog 1 (Drosophila) | NM_002822 | 7,39016E-08 | -1,65624 |
| 11747929_a_at | 51271 | UBAP1 | ubiquitin associated protein 1 | NM_001171201 NM_001171202 NM_001171203 NM_001171204 NM_016525 NR_03 | 1,38984E-07 | -1,67064 |
| 11716265_a_at | 7319 | UBE2A | ubiquitin-conjugating enzyme E2A (RAD6 homolog) | NM_003336 NM_181762 NM_181777 | 1,77061E-10 | -1,90411 |
| 11742880_a_at | 7320 | UBE2B | ubiquitin-conjugating enzyme E2B (RAD6 homolog) | NM_003337 | 0,000159588 | -1,50031 |
| 11722028_at | 7326 | UBE2G1 | ubiquitin-conjugating enzyme E2G 1 (UBC7 homolog, yeast) | NM_003342 | 1,84788E-05 | -1,63486 |
| 11728670_a_at | 7328 | UBE2H | ubiquitin-conjugating enzyme E2H (UBC8 homolog, yeast) | NM_003344 NM_182697 | 0,00284557 | -1,65334 |
| 11728224_a_at | 51465 | UBE2J1 | ubiquitin-conjugating enzyme E2, J1 (UBC6 homolog, yeast) | NM_016021 | 2,93162E-10 | -2,62894 |
| 11718508_at | 7332 | UBE2L3 | ubiquitin-conjugating enzyme E2L 3 | NM_003347 NM_198157 NR_028436 NR_028437 | 5,26904E-05 | -2,39142 |
| 11720726_at | 197131 | UBR1 | ubiquitin protein ligase E3 component n-recognin 1 | NM_174916 | 0,000379598 | -1,63995 |
| 11742705_a_at | 51366 | UBR5 | ubiquitin protein ligase E3 component n-recognin 5 | NM_015902 | 1,28497E-08 | -1,57482 |
| 11760899_a_at | 51035 | UBXN1 | UBX domain protein 1 | NM_015853 | 1,33313E-10 | -2,78718 |
| 11749832_x_at | 165324 | UBXN2A | UBX domain protein 2A | NM_181713 | 9,15292E-05 | -1,9116 |
| 11742835_a_at | 23190 | UBXN4 | UBX domain protein 4 | NM_014607 | 0,00103701 | -1,54382 |
| 11729723_a_at | 167127 | UGT3A2 | UDP glycosyltransferase 3 family, polypeptide A2 | NM_001168316 NM_174914 NR_031764 | 2,46299E-06 | -1,60722 |
| 11748783_a_at | 29128 | UHRF1 | ubiquitin-like with PHD and ring finger domains 1 | NM_001048201 NM_013282 | 0,000743403 | -1,96617 |
| 11735995_a_at | 9097 | USP14 | ubiquitin specific peptidase 14 (tRNA-guanine transglycosylase) | NM_001037334 NM_005151 | 1,66733E-06 | -1,54673 |
| 11759627_at | 9958 | USP15 | ubiquitin specific peptidase 15 | NM_006313 | 0,000998664 | -2,12091 |
| 200083_PM_at | 23326 | USP22 | ubiquitin specific peptidase 22 | NM_015276 | 3,607E-10 | -1,52075 |
| 11749740_a_at | 9960 | USP3 | ubiquitin specific peptidase 3 | NM_006537 | 9,89525E-05 | -1,94375 |
| 11742974_s_at | 84132 | USP42 | ubiquitin specific peptidase 42 | NM_032172 | 1,9371E-12 | -3,46482 |
| 11748350_a_at | 7874 | USP7 | ubiquitin specific peptidase 7 (herpes virus-associated) | NM_003470 | 1,32664E-11 | -2,01877 |
| 11724333_a_at | 9101 | USP8 | ubiquitin specific peptidase 8 | NM_001128610 NM_001128611 NM_005154 | 7,14369E-10 | -1,76032 |
| 11725331_a_at | 8239 | USP9X | ubiquitin specific peptidase 9, X-linked | NM_001039590 NM_001039591 | 1,50897E-08 | -1,55463 |
| 11763812_at | 7404 | UTY | ubiquitously transcribed tetratricopeptide repeat gene, Y-linked | NM_007125 NM_182659 NM_182660 | 0,00353017 | -1,83284 |
| 11745991_a_at | 7405 | UVRAG | UV radiation resistance associated gene | NM_003369 | 2,06396E-11 | -1,50124 |
| 11743255_a_at | 9218 | VAPA | VAMP (vesicle-associated membrane protein)-associated protein A, 33kDa | NM_003574 NM_194434 | 8,10324E-07 | -1,55828 |
| 11758451_s_at | 7415 | VCP | valosin-containing protein | NM_007126 | 6,29911E-07 | -1,63402 |
| 11756398_a_at | 9730 | VPRBP | Vpr (HIV-1) binding protein | NM_001171904 NM_014703 | 2,63048E-14 | -2,23494 |
| 11728009_at | 29802 | VPREB3 | pre-B lymphocyte 3 | NM_013378 | 6,86742E-07 | -6,45558 |
| 11753954_x_at | 9559 | VPS26A | vacuolar protein sorting 26 homolog A (S. pombe) | NM_001035260 NM_004896 | 4,00835E-07 | -1,9213 |
| 11742695_a_at | 51322 | WAC | WW domain containing adaptor with coiled-coil | NM_016628 NM_100486 NR_024557 | 2,3259E-07 | -1,58391 |
| 11747311_s_at | 441818 51729 | WBP11 WBP11P1 | WW domain binding protein 11 WW domain binding protein 11 pseudogene 1 | NM_016312 NR_003558 | 3,52055E-12 | -1,88233 |
| 11722219_at | 11193 | WBP4 | WW domain binding protein 4 (formin binding protein 21) | NM_007187 | 1,62945E-07 | -1,62537 |
| 11760388_a_at | 91833 | WDR20 | WD repeat domain 20 | NM_144574 NM_181291 NM_181302 NM_181308 | 1,76337E-07 | -2,14055 |
| 11734909_s_at | 80232 | WDR26 | WD repeat domain 26 | NM_001115113 NM_025160 | 8,06332E-07 | -1,83257 |
| 11758867_at | 55339 | WDR33 | WD repeat domain 33 | NM_001006622 NM_001006623 NM_018383 | 0,00268567 | -1,6476 |
| 11727833_at | 23160 | WDR43 | WD repeat domain 43 | NM_015131 | 1,79841E-08 | -1,57381 |
| 11750162_a_at | 56270 | WDR45L | WDR45-like | NM_019613 | 0,000249383 | -1,5682 |
| 11743620_x_at | 123720 | WHAMM | WAS protein homolog associated with actin, golgi membranes and microtubules | NM_001080435 | 6,88475E-07 | -2,40556 |
| 11736518_a_at | 7468 | WHSC1 | Wolf-Hirschhorn syndrome candidate 1 | NM_001042424 NM_007331 NM_133330 NM_133331 NM_133334 NM_133335 | 1,65992E-05 | -1,52612 |
| 11754226_a_at | 54904 | WHSC1L1 | Wolf-Hirschhorn syndrome candidate 1-like 1 | NM_017778 NM_023034 | 3,77169E-07 | -1,62854 |
| 11759628_at | 7456 | WIPF1 | WAS/WASL interacting protein family, member 1 | NM_001077269 NM_003387 | 9,86729E-06 | -1,99291 |
| 11736645_at | 11060 | WWP2 | WW domain containing E3 ubiquitin protein ligase 2 | NM_007014 NM_199423 NM_199424 | 4,78561E-09 | -2,36905 |
| 11751494_a_at | 10138 | YAF2 | YY1 associated factor 2 | NM_005748 | 9,82846E-06 | -1,55971 |
| 11719415_a_at | 7525 | YES1 | v-yes-1 Yamaguchi sarcoma viral oncogene homolog 1 | NM_005433 | 0,00141706 | -1,64947 |
| 11715986_s_at | 51646 | YPEL5 | yippee-like 5 (Drosophila) | NM_001127399 NM_001127400 NM_001127401 NM_016061 | 2,75955E-05 | -1,52068 |
| 11740514_a_at | 91746 | YTHDC1 | YTH domain containing 1 | NM_001031732 NM_133370 | 4,45117E-07 | -2,49418 |
| 11745511_a_at | 54915 | YTHDF1 | YTH domain family, member 1 | NM_017798 | 1,88708E-06 | -1,65284 |
| 11758751_at | 7534 | YWHAZ | tyrosine 3-monooxygenase/tryptophan 5-monooxygenase activation protein, zeta polypeptid | NM_001135699 NM_001135700 NM_001135701 NM_001135702 NM_003406 NM_14 | 0,000037896 | -1,57065 |
| 11758916_at | 7528 | YY1 | YY1 transcription factor | NM_003403 | 1,77505E-12 | -1,69452 |
| 11722277_s_at | 9889 | ZBED4 | zinc finger, BED-type containing 4 | NM_014838 | 5,02816E-12 | -1,71494 |
| 11745435_a_at | 22890 | ZBTB1 | zinc finger and BTB domain containing 1 | NM_001123329 NM_014950 | 5,03057E-06 | -1,59341 |
| 11727339_a_at | 57621 | ZBTB2 | zinc finger and BTB domain containing 2 | NM_020861 | 5,33533E-11 | -1,89632 |
| 11744810_a_at | 9841 | ZBTB24 | zinc finger and BTB domain containing 24 | NM_001164313 NM_014797 | 4,00835E-07 | -1,74893 |
| 11738156_a_at | 57684 | ZBTB26 | zinc finger and BTB domain containing 26 | NM_020924 | 2,91993E-05 | -1,62664 |
| 11725236_at | 9880 | ZBTB39 | zinc finger and BTB domain containing 39 | NM_014830 | 5,68596E-09 | -1,5765 |
| 11722288_a_at | 23099 | ZBTB43 | zinc finger and BTB domain containing 43 | NM_001135776 NM_014007 | 2,17379E-06 | -2,26848 |
| 11717815_at | 9925 | ZBTB5 | zinc finger and BTB domain containing 5 | NM_014872 | 2,81268E-05 | -1,7789 |
| 11730939_at | 653121 | ZBTB8A | zinc finger and BTB domain containing 8A | NM_001040441 | 0,000958225 | -1,79541 |
| 11763472_x_at | 340152 | ZC3H12D | zinc finger CCCH-type containing 12D | NM_207360 | 0,00146362 | -1,83057 |
| 11759529_at | 55854 | ZC3H15 | zinc finger CCCH-type containing 15 | NM_018471 | 8,02454E-07 | -1,80738 |
| 11759647_x_at | 29066 | ZC3H7A | zinc finger CCCH-type containing 7A | NM_014153 | 2,12449E-09 | -1,56857 |
| 11756839_x_at | 56829 | ZC3HAV1 | zinc finger CCCH-type, antiviral 1 | NM_020119 NM_024625 | 1,10011E-06 | -1,92221 |
| 11743591_a_at | 55625 | ZDHHC7 | zinc finger, DHHC-type containing 7 | NM_001145548 NM_017740 | 5,5847E-06 | -1,55586 |
| 11723430_a_at | 386607 80829 | ZFP91 ZFP91-CNTF | zinc finger protein 91 homolog (mouse) ZFP91-CNTF readthrough transcript | NM_053023 NR_024091 | 4,07009E-09 | -1,73469 |
| 11760665_at | 7586 | ZKSCAN1 | zinc finger with KRAB and SCAN domains 1 | NM_003439 | 0,000416717 | -1,92441 |
| 11735636_a_at | 9205 | ZMYM5 | zinc finger, MYM-type 5 | NM_001039649 NM_001039650 NM_001142684 | 0,00199114 | -1,67506 |
| 11743889_a_at | 7556 | ZNF10 | zinc finger protein 10 | NM_015394 | 4,99281E-06 | -1,60271 |
| 11730577_at | 163227 | ZNF100 | zinc finger protein 100 | NM_173531 | 1,24506E-10 | -1,79978 |
| 11749808_x_at | 51427 | ZNF107 | zinc finger protein 107 | NM_001013746 NM_016220 | 3,67809E-08 | -1,5471 |
| 11737217_s_at | 7678 | ZNF124 | zinc finger protein 124 | NM_003431 | 1,51233E-05 | -1,75542 |
| 11746383_a_at | 7690 | ZNF131 | zinc finger protein 131 | NM_003432 | 2,12661E-09 | -1,50764 |
| 11731497_a_at | 7693 | ZNF134 | zinc finger protein 134 | NM_003435 | 1,8387E-06 | -1,76931 |
| 11736277_x_at | 7695 | ZNF136 | zinc finger protein 136 | NM_003437 | 5,15787E-05 | -1,87635 |
| 11728597_a_at | 7673 | ZNF222 | zinc finger protein 222 | NM_001129996 NM_013360 | 0,00215671 | -1,7654 |
| 11721601_s_at | 10472 | ZNF238 | zinc finger protein 238 | NM_006352 NM_205768 | 4,93825E-11 | -1,78023 |
| 11723259_x_at | 7572 | ZNF24 | zinc finger protein 24 | NM_006965 | 2,01424E-06 | -1,77648 |
| 11762349_x_at | 9534 | ZNF254 | zinc finger protein 254 | NM_203282 | 6,72156E-07 | -2,38178 |
| 11758517_s_at | 10782 | ZNF274 | zinc finger protein 274 | NM_016324 NM_016325 NM_133502 | 7,00549E-09 | -1,60099 |
| 11729733_a_at | 23528 | ZNF281 | zinc finger protein 281 | NM_012482 | 2,48119E-05 | -1,53169 |
| 11759507_at | 23036 | ZNF292 | zinc finger protein 292 | NM_015021 | 6,42541E-08 | -1,66146 |
| 11758414_s_at | 49854 | ZNF295 | zinc finger protein 295 | NM_001098402 NM_001098403 NM_020727 | 4,45238E-08 | -3,42355 |
| 11722817_a_at | 7551 | ZNF3 | zinc finger protein 3 | NM_017715 NM_032924 | 6,68233E-11 | -1,66591 |
| 11728261_a_at | 91975 | ZNF300 | zinc finger protein 300 | NM_001172831 NM_001172832 NM_052860 | 0,00257804 | -1,5144 |
| 11741798_a_at | 57343 | ZNF304 | zinc finger protein 304 | NM_020657 | 1,05034E-06 | -1,59527 |
| 11735748_a_at | 57693 | ZNF317 | zinc finger protein 317 | NM_020933 | 6,24434E-09 | -1,58744 |
| 11732398_a_at | 284695 | ZNF326 | zinc finger protein 326 | NM_182975 NM_182976 | 1,81902E-07 | -1,77628 |
| 11729766_x_at | 55422 | ZNF331 | zinc finger protein 331 | NM_001079906 NM_001079907 NM_018555 | 2,03342E-10 | -2,23201 |
| 11732610_at | 79750 | ZNF385D | zinc finger protein 385D | NM_024697 | 0,018696 | -1,62754 |
| 11729609_a_at | 84124 | ZNF394 | zinc finger protein 394 | NM_032164 | 0,000033652 | -1,63017 |
| 11732013_a_at | 79744 | ZNF419 | zinc finger protein 419 | NM_001098491 NM_001098492 NM_001098493 NM_001098494 NM_001098495 NM | 4,92047E-07 | -1,63753 |
| 11736977_x_at | 170959 | ZNF431 | zinc finger protein 431 | NM_133473 | 1,49847E-07 | -1,74785 |
| 11755232_s_at | 90333 | ZNF468 | zinc finger protein 468 | NM_001008801 NM_199132 | 4,88184E-07 | -1,54653 |
| 11729346_s_at | 130557 | ZNF513 | zinc finger protein 513 | NM_144631 | 3,07258E-06 | -1,51284 |
| 11755645_a_at | 57711 | ZNF529 | zinc finger protein 529 | NM_001145649 NM_001145650 NM_020951 NR_027239 | 5,53147E-05 | -1,62816 |
| 11731703_x_at | 90233 | ZNF551 | zinc finger protein 551 | NM_138347 | 6,15629E-07 | -1,53006 |
| 11735008_a_at | 148254 | ZNF555 | zinc finger protein 555 | NM_001172775 NM_152791 | 0,00729832 | -1,72082 |
| 11756696_x_at | 163081 | ZNF567 | zinc finger protein 567 | NM_152603 | 1,08647E-06 | -1,68154 |
| 11730981_x_at | 152687 | ZNF595 | zinc finger protein 595 | NM_182524 | 5,53227E-07 | -3,19876 |
| 11726449_at | 80110 | ZNF614 | zinc finger protein 614 | NM_025040 | 8,95561E-08 | -1,568 |
| 11746271_a_at | 51193 | ZNF639 | zinc finger protein 639 | NM_016331 | 4,92807E-07 | -1,72237 |
| 11715154_s_at | 171392 | ZNF675 | zinc finger protein 675 | NM_138330 | 6,08827E-05 | -1,55975 |
| 11754636_a_at | 7562 | ZNF708 | zinc finger protein 708 | NM_021269 | 1,95247E-06 | -1,74641 |
| 11731962_a_at | 163051 | ZNF709 | zinc finger protein 709 | NM_001145647 NM_152601 | 0,00874238 | -1,84652 |
| 11746460_a_at | 7552 | ZNF711 | zinc finger protein 711 | NM_021998 | 0,00855834 | -1,53522 |
| 11723787_x_at | 90321 | ZNF766 | zinc finger protein 766 | NM_001010851 | 2,99872E-06 | -1,6691 |
| 11730548_a_at | 285989 | ZNF789 | zinc finger protein 789 | NM_001013258 NM_213603 | 1,89225E-05 | -1,83522 |
| 11749755_a_at | 163049 | ZNF791 | zinc finger protein 791 | NM_153358 | 2,53836E-05 | -2,15816 |
| 11736419_a_at | 168850 | ZNF800 | zinc finger protein 800 | NM_176814 | 1,46774E-06 | -1,79529 |
| 11739609_a_at | 390980 | ZNF805 | zinc finger protein 805 | NM_001023563 NM_001145078 | 9,68915E-09 | -1,82945 |
| 11754540_x_at | 730051 | ZNF814 | zinc finger protein 814 | NM_001144989 | 0,000117878 | -1,60474 |
| 11759750_at | 400713 | ZNF880 | zinc finger protein 880 | NM_001145434 | 2,22628E-06 | -2,49896 |
| 11732320_a_at | 168374 | ZNF92 | zinc finger protein 92 | NM_007139 NM_152626 | 7,80391E-08 | -1,517 |
| 11758596_s_at | 54764 | ZRANB1 | zinc finger, RAN-binding domain containing 1 | NM_017580 | 1,70199E-08 | -1,82159 |
| 11735582_at | 54993 | ZSCAN2 | zinc finger and SCAN domain containing 2 | NM_001007072 NM_017894 NM_181877 | 3,23276E-05 | -1,61479 |
| 11755138_s_at | 158586 | ZXDB | zinc finger, X-linked, duplicated B | NM_007157 | 1,61971E-05 | -1,56158 |
| 11727464_at | 79364 | ZXDC | ZXD family zinc finger C | NM_001040653 NM_025112 | 2,53546E-05 | -1,69667 |

**Table 3 : Differentially expressed genes (DEGs) upon miR-494-3p overexpression.** This table represents the list of DEGs upon miR-494-3p overexpression in CB CD34+ cells according to microarray analysis performed 24 hours after transfection.

| **Column ID** | **Entrez Gene** | **Gene Symbol** | **Gene Title** | **RefSeq Transcript ID** | **p-value** | **Fold-Change** | **Fold-Change (Description)** |
| --- | --- | --- | --- | --- | --- | --- | --- |
| 11754418_s_at | 10146 | G3BP1 | GTPase activating protein (SH3 domain) binding protein 1 | NM_005754 NM_198395 | 0,00117655 | -4,40926 | 494 down vs NEG |
| 11730759_s_at | 9419 | CRIPT | cysteine-rich PDZ-binding protein | NM_014171 | 0,0328274 | -3,56291 | 494 down vs NEG |
| 11722586_a_at | 10146 | G3BP1 | GTPase activating protein (SH3 domain) binding protein 1 | NM_005754 NM_198395 | 0,0124337 | -3,41808 | 494 down vs NEG |
| 11749546_a_at | 25800 | SLC39A6 | solute carrier family 39 (zinc transporter), member 6 | NM_001099406 NM_012319 | 0,0129936 | -3,34079 | 494 down vs NEG |
| 11757587_s_at | 25800 | SLC39A6 | solute carrier family 39 (zinc transporter), member 6 | NM_001099406 NM_012319 | 0,021003 | -3,2894 | 494 down vs NEG |
| 11746111_a_at | 10146 | G3BP1 | GTPase activating protein (SH3 domain) binding protein 1 | NM_005754 NM_198395 | 0,0220517 | -3,2345 | 494 down vs NEG |
| 11717726_s_at | 51155 | HN1 | hematological and neurological expressed 1 | NM_001002032 NM_001002033 NM_016185 | 0,00575927 | -3,13797 | 494 down vs NEG |
| 11717150_s_at | 79650 | C16orf57 | chromosome 16 open reading frame 57 | NM_024598 | 0,00248123 | -3,07335 | 494 down vs NEG |
| 11716468_s_at | 389293 81689 | ISCA1 ISCA1P1 | iron-sulfur cluster assembly 1 homolog (S. cerevisiae) iron-sulfur cluster assembly | NM_001080540 NM_030940 | 0,0025797 | -3,05682 | 494 down vs NEG |
| 11758773_at | 201562 | PTPLB | protein tyrosine phosphatase-like (proline instead of catalytic arginine), member b | NM_198402 | 0,00338456 | -2,94341 | 494 down vs NEG |
| 11740656_a_at | 8091 | HMGA2 | high mobility group AT-hook 2 | NM_003483 NM_003484 | 0,00125248 | -2,70439 | 494 down vs NEG |
| 11759612_at | 64778 | FNDC3B | fibronectin type III domain containing 3B | NM_001135095 NM_022763 | 0,0243795 | -2,69384 | 494 down vs NEG |
| 11744286_s_at | 875 | CBS | cystathionine-beta-synthase | NM_000071 NM_001178008 NM_001178009 | 0,0340539 | -2,69366 | 494 down vs NEG |
| 11722213_at | 10360 | NPM3 | nucleophosmin/nucleoplasmin 3 | NM_006993 | 0,00505185 | -2,52063 | 494 down vs NEG |
| 11719690_at | 57045 | TWSG1 | twisted gastrulation homolog 1 (Drosophila) | NM_020648 | 0,0450143 | -2,50419 | 494 down vs NEG |
| 11730111_a_at | 55635 | DEPDC1 | DEP domain containing 1 | NM_001114120 NM_017779 | 0,0461813 | -2,47303 | 494 down vs NEG |
| 11735415_a_at | 25800 | SLC39A6 | solute carrier family 39 (zinc transporter), member 6 | NM_001099406 NM_012319 | 0,00236047 | -2,46496 | 494 down vs NEG |
| 11733055_a_at | 9522 | SCAMP1 | secretory carrier membrane protein 1 | NM_004866 | 0,0123672 | -2,4322 | 494 down vs NEG |
| 11717296_s_at | 10128 | LRPPRC | leucine-rich PPR-motif containing | NM_133259 | 0,00328547 | -2,34072 | 494 down vs NEG |
| 11754598_s_at | 9093 | DNAJA3 | DnaJ (Hsp40) homolog, subfamily A, member 3 | NM_001135110 NM_005147 | 0,00724876 | -2,3243 | 494 down vs NEG |
| 11717727_s_at | 51155 | HN1 | hematological and neurological expressed 1 | NM_001002032 NM_001002033 NM_016185 | 0,000303081 | -2,31609 | 494 down vs NEG |
| 11743480_s_at | 9821 | RB1CC1 | RB1-inducible coiled-coil 1 | NM_001083617 NM_014781 | 0,0151248 | -2,28783 | 494 down vs NEG |
| 11715305_s_at | 3206 | HOXA10 | homeobox A10 | NM_018951 NM_153715 | 0,00332499 | -2,27606 | 494 down vs NEG |
| 11718843_s_at | 2790 552891 | DNAJC25-GNG10 GNG10 | DNAJC25-GNG10 readthrough guanine nucleotide binding protein (G protein), gamma 10 | NM_001017998 NM_004125 | 0,0134402 | -2,27357 | 494 down vs NEG |
| 11716507_at | 8452 | CUL3 | cullin 3 | NM_003590 | 0,0477862 | -2,26788 | 494 down vs NEG |
| 11733825_s_at | 84886 | C1orf198 | chromosome 1 open reading frame 198 | NM_001136494 NM_001136495 NM_032800 | 0,0470083 | -2,25617 | 494 down vs NEG |
| 11715546_s_at | 10951 | CBX1 | chromobox homolog 1 (HP1 beta homolog Drosophila ) | NM_001127228 NM_006807 | 0,0183678 | -2,24088 | 494 down vs NEG |
| 11733077_a_at | 4659 | PPP1R12A | protein phosphatase 1, regulatory (inhibitor) subunit 12A | NM_001143885 NM_001143886 NM_002480 | 0,00146827 | -2,21641 | 494 down vs NEG |
| 11739302_a_at | 8323 | FZD6 | frizzled homolog 6 (Drosophila) | NM_001164615 NM_001164616 NM_003506 | 0,0127674 | -2,18973 | 494 down vs NEG |
| 11727426_a_at | 821 | CANX | calnexin | NM_001024649 NM_001746 | 0,0162314 | -2,18747 | 494 down vs NEG |
| 11753379_a_at | 5594 | MAPK1 | mitogen-activated protein kinase 1 | NM_002745 NM_138957 | 0,00667809 | -2,13374 | 494 down vs NEG |
| 11723930_at | 22948 | CCT5 | chaperonin containing TCP1, subunit 5 (epsilon) | NM_012073 | 0,0312457 | -2,13175 | 494 down vs NEG |
| 11737879_s_at | 2590 | GALNT2 | UDP-N-acetyl-alpha-D-galactosamine:polypeptide N-acetylgalactosaminyltransferase 2 (Gal | NM_004481 | 0,00869708 | -2,0845 | 494 down vs NEG |
| 11738871_a_at | 5594 | MAPK1 | mitogen-activated protein kinase 1 | NM_002745 NM_138957 | 0,0131439 | -2,07406 | 494 down vs NEG |
| 11721955_at | 64864 | RFX7 | regulatory factor X, 7 | NM_022841 | 0,0232618 | -2,05998 | 494 down vs NEG |
| 11738872_x_at | 5594 | MAPK1 | mitogen-activated protein kinase 1 | NM_002745 NM_138957 | 0,00723796 | -2,04718 | 494 down vs NEG |
| 11758491_s_at | 8540 | AGPS | alkylglycerone phosphate synthase | NM_003659 | 0,0239197 | -2,03707 | 494 down vs NEG |
| 11722410_at | 51809 | GALNT7 | UDP-N-acetyl-alpha-D-galactosamine:polypeptide N-acetylgalactosaminyltransferase 7 (Gal | NM_017423 | 0,00552232 | -2,01511 | 494 down vs NEG |
| 11729600_a_at | 5537 | PPP6C | protein phosphatase 6, catalytic subunit | NM_001123355 NM_001123369 NM_002721 | 0,0435534 | -1,99474 | 494 down vs NEG |
| 11719198_s_at | 894 | CCND2 | cyclin D2 | NM_001759 | 0,00778626 | -1,97127 | 494 down vs NEG |
| 11758199_s_at | 5887 | RAD23B | RAD23 homolog B (S. cerevisiae) | NM_002874 | 0,045216 | -1,96462 | 494 down vs NEG |
| 11751223_a_at | 5594 | MAPK1 | mitogen-activated protein kinase 1 | NM_002745 NM_138957 | 0,0142436 | -1,95926 | 494 down vs NEG |
| 11733261_a_at | 10659 | CELF2 | CUGBP, Elav-like family member 2 | NM_001025076 NM_001025077 NM_001083591 NM_006561 | 0,00500318 | -1,94387 | 494 down vs NEG |
| 11726136_a_at | 286527 | TMSB15B | thymosin beta 15B | NM_194324 | 0,0162099 | -1,92813 | 494 down vs NEG |
| 11751309_a_at | 9093 | DNAJA3 | DnaJ (Hsp40) homolog, subfamily A, member 3 | NM_001135110 NM_005147 | 0,00182625 | -1,9194 | 494 down vs NEG |
| 11752183_s_at | 9093 | DNAJA3 | DnaJ (Hsp40) homolog, subfamily A, member 3 | NM_001135110 NM_005147 | 0,0152677 | -1,91644 | 494 down vs NEG |
| 11717297_at | 10128 | LRPPRC | leucine-rich PPR-motif containing | NM_133259 | 0,00978165 | -1,89992 | 494 down vs NEG |
| 11737878_a_at | 2590 | GALNT2 | UDP-N-acetyl-alpha-D-galactosamine:polypeptide N-acetylgalactosaminyltransferase 2 (Gal | NM_004481 | 0,0160498 | -1,89676 | 494 down vs NEG |
| 11721446_a_at | 8723 | SNX4 | sorting nexin 4 | NM_003794 | 0,00968589 | -1,89598 | 494 down vs NEG |
| 11722408_at | 51809 | GALNT7 | UDP-N-acetyl-alpha-D-galactosamine:polypeptide N-acetylgalactosaminyltransferase 7 (Gal | NM_017423 | 0,0160538 | -1,88665 | 494 down vs NEG |
| 11730760_x_at | 9419 | CRIPT | cysteine-rich PDZ-binding protein | NM_014171 | 0,025926 | -1,88025 | 494 down vs NEG |
| 11722535_a_at | 151195 | CCNYL1 | cyclin Y-like 1 | NM_001142300 NM_152523 | 0,0107836 | -1,87976 | 494 down vs NEG |
| 11715728_a_at | 3688 | ITGB1 | integrin, beta 1 (fibronectin receptor, beta polypeptide, antigen CD29 includes MDF2, M | NM_002211 NM_033666 NM_033667 NM_033668 NM_033669 NM_133376 | 0,00112417 | -1,879 | 494 down vs NEG |
| 11741433_a_at | 372 | ARCN1 | archain 1 | NM_001142281 NM_001655 | 0,0188914 | -1,87572 | 494 down vs NEG |
| 11718158_a_at | 9111 | NMI | N-myc (and STAT) interactor | NM_004688 | 0,018746 | -1,84638 | 494 down vs NEG |
| 11754109_s_at | 332 | BIRC5 | baculoviral IAP repeat-containing 5 | NM_001012270 NM_001012271 NM_001168 | 0,00119447 | -1,83466 | 494 down vs NEG |
| 11718159_at | 9111 | NMI | N-myc (and STAT) interactor | NM_004688 | 0,0160706 | -1,83325 | 494 down vs NEG |
| 11746027_a_at | 9111 | NMI | N-myc (and STAT) interactor | NM_004688 | 0,0176613 | -1,83151 | 494 down vs NEG |
| 11721637_s_at | 79139 | DERL1 | Der1-like domain family, member 1 | NM_001134671 NM_024295 | 0,0190783 | -1,82715 | 494 down vs NEG |
| 11734049_a_at | 81929 | SEH1L | SEH1-like (S. cerevisiae) | NM_001013437 NM_031216 | 0,00914485 | -1,81143 | 494 down vs NEG |
| 11764246_s_at | 201595 | STT3B | STT3, subunit of the oligosaccharyltransferase complex, homolog B (S. cerevisiae) | NM_178862 | 0,0467395 | -1,79998 | 494 down vs NEG |
| 11757599_x_at | 10204 | NUTF2 | nuclear transport factor 2 | NM_005796 | 0,0170315 | -1,79842 | 494 down vs NEG |
| 11723781_at | 8540 | AGPS | alkylglycerone phosphate synthase | NM_003659 | 0,00430987 | -1,79 | 494 down vs NEG |
| 11763303_at | 414327 | PS1TP4 | HBV preS1-transactivated protein 4 | --- | 0,0172003 | -1,78323 | 494 down vs NEG |
| 11721635_a_at | 79139 | DERL1 | Der1-like domain family, member 1 | NM_001134671 NM_024295 | 0,0181348 | -1,78082 | 494 down vs NEG |
| 11717149_a_at | 79650 | C16orf57 | chromosome 16 open reading frame 57 | NM_024598 | 0,0319174 | -1,77904 | 494 down vs NEG |
| 11753758_x_at | 10204 | NUTF2 | nuclear transport factor 2 | NM_005796 | 0,0135596 | -1,77609 | 494 down vs NEG |
| 11727433_s_at | 10204 | NUTF2 | nuclear transport factor 2 | NM_005796 | 0,00480657 | -1,77003 | 494 down vs NEG |
| 11731624_a_at | 55088 | C10orf118 | chromosome 10 open reading frame 118 | NM_018017 | 0,0219794 | -1,76931 | 494 down vs NEG |
| 11727432_a_at | 10204 | NUTF2 | nuclear transport factor 2 | NM_005796 | 0,0205634 | -1,76206 | 494 down vs NEG |
| 11758454_s_at | 201627 | FAM116A | family with sequence similarity 116, member A | NM_152678 | 0,0292907 | -1,75955 | 494 down vs NEG |
| 11753757_a_at | 10204 | NUTF2 | nuclear transport factor 2 | NM_005796 | 0,0149449 | -1,7571 | 494 down vs NEG |
| 11719335_a_at | 196513 | DCP1B | DCP1 decapping enzyme homolog B (S. cerevisiae) | NM_152640 | 0,0213528 | -1,74027 | 494 down vs NEG |
| 11758440_s_at | 64864 | RFX7 | regulatory factor X, 7 | NM_022841 | 0,0204278 | -1,73294 | 494 down vs NEG |
| 11757938_s_at | 9111 | NMI | N-myc (and STAT) interactor | NM_004688 | 0,0149308 | -1,73076 | 494 down vs NEG |
| 11737747_a_at | 2764 | GMFB | glia maturation factor, beta | NM_004124 | 0,0445714 | -1,73041 | 494 down vs NEG |
| 11721388_a_at | 5026 | P2RX5 | purinergic receptor P2X, ligand-gated ion channel, 5 | NM_002561 NM_175080 NM_175081 | 0,00194013 | -1,70958 | 494 down vs NEG |
| 11720237_s_at | 5601 | MAPK9 | mitogen-activated protein kinase 9 | NM_001135044 NM_002752 NM_139068 NM_139069 NM_139070 | 0,0229272 | -1,70722 | 494 down vs NEG |
| 11736596_s_at | 84296 | GINS4 | GINS complex subunit 4 (Sld5 homolog) | NM_032336 | 0,0248512 | -1,70688 | 494 down vs NEG |
| 11745196_a_at | 55256 | ADI1 | acireductone dioxygenase 1 | NM_018269 | 0,0274157 | -1,70583 | 494 down vs NEG |
| 11722159_a_at | 23428 | SLC7A8 | solute carrier family 7 (amino acid transporter, L-type), member 8 | NM_012244 NM_182728 | 0,00225844 | -1,6947 | 494 down vs NEG |
| 11717626_a_at | 4718 | NDUFC2 | NADH dehydrogenase (ubiquinone) 1, subcomplex unknown, 2, 14.5kDa | NM_004549 | 0,025229 | -1,69413 | 494 down vs NEG |
| 11725277_a_at | 117854 | TRIM6 | tripartite motif-containing 6 | NM_001003818 NM_058166 | 0,0154867 | -1,69157 | 494 down vs NEG |
| 11757564_x_at | 55256 | ADI1 | acireductone dioxygenase 1 | NM_018269 | 0,041238 | -1,68862 | 494 down vs NEG |
| 11746028_x_at | 9111 | NMI | N-myc (and STAT) interactor | NM_004688 | 0,0415391 | -1,67638 | 494 down vs NEG |
| 11758200_x_at | 1163 | CKS1B | CDC28 protein kinase regulatory subunit 1B | NM_001826 NR_024163 | 0,0492428 | -1,66996 | 494 down vs NEG |
| 11757512_a_at | 55256 | ADI1 | acireductone dioxygenase 1 | NM_018269 | 0,0238725 | -1,66286 | 494 down vs NEG |
| 11720096_a_at | 80774 | LIMD2 | LIM domain containing 2 | NM_030576 | 0,0429653 | -1,66147 | 494 down vs NEG |
| 11758981_a_at | 10204 | NUTF2 | Nuclear transport factor 2 | NM_005796 | 0,0117099 | -1,65958 | 494 down vs NEG |
| 11758088_s_at | 9306 | SOCS6 | suppressor of cytokine signaling 6 | NM_004232 | 0,000645397 | -1,65032 | 494 down vs NEG |
| 11739603_x_at | 332 | BIRC5 | baculoviral IAP repeat-containing 5 | NM_001012270 NM_001012271 NM_001168 | 0,0124399 | -1,64526 | 494 down vs NEG |
| 11751224_x_at | 5594 | MAPK1 | mitogen-activated protein kinase 1 | NM_002745 NM_138957 | 0,0124702 | -1,63973 | 494 down vs NEG |
| 11754110_x_at | 332 | BIRC5 | baculoviral IAP repeat-containing 5 | NM_001012270 NM_001012271 NM_001168 | 0,0011658 | -1,63066 | 494 down vs NEG |
| 11723558_s_at | 9643 | MORF4L2 | mortality factor 4 like 2 | NM_001142418 NM_001142419 NM_001142420 NM_001142421 NM_001142422 NM | 0,0245706 | -1,62738 | 494 down vs NEG |
| 11718678_a_at | 5663 | PSEN1 | presenilin 1 | NM_000021 NM_007318 | 0,008759 | -1,62259 | 494 down vs NEG |
| 11752360_s_at | 55696 | RBM22 | RNA binding motif protein 22 | NM_018047 | 0,00459402 | -1,5987 | 494 down vs NEG |
| 11715897_at | 950 | SCARB2 | scavenger receptor class B, member 2 | NM_005506 | 0,0432837 | -1,58761 | 494 down vs NEG |
| 11749898_a_at | 9522 | SCAMP1 | secretory carrier membrane protein 1 | NM_004866 | 0,00853212 | -1,57072 | 494 down vs NEG |
| 11730469_s_at | 4005 | LMO2 | LIM domain only 2 (rhombotin-like 1) | NM_001142315 NM_001142316 NM_005574 | 0,00840777 | -1,56918 | 494 down vs NEG |
| 11755289_x_at | 9643 | MORF4L2 | mortality factor 4 like 2 | NM_001142418 NM_001142419 NM_001142420 NM_001142421 NM_001142422 NM | 0,0416482 | -1,56855 | 494 down vs NEG |
| 11747720_a_at | 332 | BIRC5 | baculoviral IAP repeat-containing 5 | NM_001012270 NM_001012271 NM_001168 | 0,014465 | -1,56319 | 494 down vs NEG |
| 11751145_a_at | 23376 | KIAA0776 | KIAA0776 | NM_015323 | 0,00872394 | -1,56279 | 494 down vs NEG |
| 11722721_a_at | 201161 | CENPV | centromere protein V | NM_181716 | 0,0360991 | -1,55898 | 494 down vs NEG |
| 11721944_x_at | 11158 | RABL2B | RAB, member of RAS oncogene family-like 2B | NM_001003789 NM_001130919 NM_001130920 NM_001130921 NM_001130922 NM | 0,0161071 | -1,55792 | 494 down vs NEG |
| 11723343_at | 23087 | TRIM35 | tripartite motif-containing 35 | NM_171982 | 0,00137506 | -1,5504 | 494 down vs NEG |
| 11715813_at | 25994 | HIGD1A | HIG1 hypoxia inducible domain family, member 1A | NM_001099668 NM_001099669 NM_014056 | 0,0046263 | -1,54673 | 494 down vs NEG |
| 11731919_a_at | 7112 | TMPO | thymopoietin | NM_001032283 NM_001032284 NM_003276 | 0,0436653 | -1,54158 | 494 down vs NEG |
| 11715550_at | 1605 | DAG1 | dystroglycan 1 (dystrophin-associated glycoprotein 1) | NM_001165928 NM_001177634 NM_001177635 NM_001177636 NM_001177637 NM | 0,00224903 | -1,54073 | 494 down vs NEG |
| 11747498_s_at | 6652 | SORD | sorbitol dehydrogenase | NM_003104 | 0,034365 | -1,5384 | 494 down vs NEG |
| 11751051_a_at | 57062 | DDX24 | DEAD (Asp-Glu-Ala-Asp) box polypeptide 24 | NM_020414 | 0,0277671 | -1,5364 | 494 down vs NEG |
| 11730112_a_at | 55635 | DEPDC1 | DEP domain containing 1 | NM_001114120 NM_017779 | 0,0362702 | -1,53625 | 494 down vs NEG |
| 11733864_a_at | 332 | BIRC5 | baculoviral IAP repeat-containing 5 | NM_001012270 NM_001012271 NM_001168 | 0,0107935 | -1,53605 | 494 down vs NEG |
| 11717908_s_at | 84908 | FAM136A | family with sequence similarity 136, member A | NM_032822 | 0,0366619 | -1,53242 | 494 down vs NEG |
| 11717841_at | 55500 | ETNK1 | ethanolamine kinase 1 | NM_001039481 NM_018638 | 0,0363622 | -1,53029 | 494 down vs NEG |
| 11757793_s_at | 7035 | TFPI | tissue factor pathway inhibitor (lipoprotein-associated coagulation inhibitor) | NM_001032281 NM_006287 | 0,0193375 | -1,53005 | 494 down vs NEG |
| 11741556_x_at | 79647 | AKIRIN1 | akirin 1 | NM_001136275 NM_024595 | 0,00478824 | -1,52545 | 494 down vs NEG |
| 11721612_a_at | 51194 | IPO11 | importin 11 | NM_001134779 NM_016338 | 0,0385234 | -1,52198 | 494 down vs NEG |
| 11742740_at | 25929 | GEMIN5 | gem (nuclear organelle) associated protein 5 | NM_015465 | 0,0470382 | -1,51787 | 494 down vs NEG |
| 11723360_x_at | 5871 | MAP4K2 | mitogen-activated protein kinase kinase kinase kinase 2 | NM_004579 | 0,00885676 | -1,51635 | 494 down vs NEG |
| 11728405_s_at | 375387 | LRRC33 | leucine rich repeat containing 33 | NM_198565 | 0,00541394 | -1,51417 | 494 down vs NEG |
| 11763073_x_at | 332 | BIRC5 | baculoviral IAP repeat-containing 5 | NM_001012270 NM_001012271 NM_001168 | 0,0292786 | -1,51388 | 494 down vs NEG |
| 11725523_at | 1829 | DSG2 | desmoglein 2 | NM_001943 | 0,0161341 | -1,51238 | 494 down vs NEG |
| 11731506_a_at | 5887 | RAD23B | RAD23 homolog B (S. cerevisiae) | NM_002874 | 0,0159837 | -1,50651 | 494 down vs NEG |
| 11722398_s_at | 201965 | RWDD4A | RWD domain containing 4A | NM_152682 | 0,0149195 | -1,50292 | 494 down vs NEG |
| 11733899_a_at | 6738 | TROVE2 | TROVE domain family, member 2 | NM_001042369 NM_001042370 NM_001173524 NM_001173525 NM_004600 NR_03 | 0,0100684 | -1,50046 | 494 down vs NEG |
| 11763585_s_at | 7112 | TMPO | thymopoietin | NM_001032283 NM_001032284 NM_003276 | 0,0148551 | -1,50011 | 494 down vs NEG |
| 11730090_a_at | 407977 8741 | TNFSF12-TNFSF13 TNFSF13 | TNFSF12-TNFSF13 readthrough tumor necrosis factor (ligand) superfamily, member 13 | NM_003808 NM_172087 NM_172088 NM_172089 | 0,00892159 | 1,50087 | 494 up vs NEG |
| 11729201_at | 387263 | C6orf120 | chromosome 6 open reading frame 120 | NM_001029863 | 0,00650627 | 1,50653 | 494 up vs NEG |
| 11739367_at | 444 | ASPH | aspartate beta-hydroxylase | NM_001164750 NM_001164751 NM_001164752 NM_001164753 NM_001164754 NM | 0,0170838 | 1,50815 | 494 up vs NEG |
| 11723214_a_at | 4208 | MEF2C | myocyte enhancer factor 2C | NM_001131005 NM_002397 | 0,00854499 | 1,5093 | 494 up vs NEG |
| 11723177_at | 80144 | FRAS1 | Fraser syndrome 1 | NM_001166133 NM_025074 | 0,045502 | 1,50988 | 494 up vs NEG |
| 11752549_x_at | 216 | ALDH1A1 | aldehyde dehydrogenase 1 family, member A1 | NM_000689 | 0,0427626 | 1,51205 | 494 up vs NEG |
| 11723863_a_at | 55619 | DOCK10 | dedicator of cytokinesis 10 | NM_014689 | 0,0368101 | 1,52029 | 494 up vs NEG |
| 11722209_a_at | 5742 | PTGS1 | prostaglandin-endoperoxide synthase 1 (prostaglandin G/H synthase and cyclooxygenase) | NM_000962 NM_080591 | 0,00624599 | 1,52074 | 494 up vs NEG |
| 11758081_s_at | 2029 | ENSA | endosulfine alpha | NM_004436 NM_207042 NM_207043 NM_207044 NM_207045 NM_207046 NM_ | 0,0110171 | 1,52471 | 494 up vs NEG |
| 11726806_s_at | 114971 | PTPMT1 | protein tyrosine phosphatase, mitochondrial 1 | NM_001143984 NM_175732 | 0,0292736 | 1,53178 | 494 up vs NEG |
| 11723562_a_at | 4013 | VWA5A | von Willebrand factor A domain containing 5A | NM_001130142 NM_014622 NM_198315 | 0,0305192 | 1,53422 | 494 up vs NEG |
| 11721790_s_at | 5789 | PTPRD | protein tyrosine phosphatase, receptor type, D | NM_001040712 NM_001171025 NM_002839 NM_130391 NM_130392 NM_130393 | 0,00535403 | 1,53681 | 494 up vs NEG |
| 11724118_a_at | 219285 | SAMD9L | sterile alpha motif domain containing 9-like | NM_152703 | 0,0125193 | 1,53721 | 494 up vs NEG |
| 11753730_a_at | 6622 | SNCA | synuclein, alpha (non A4 component of amyloid precursor) | NM_000345 NM_001146054 NM_001146055 NM_007308 | 0,019955 | 1,53721 | 494 up vs NEG |
| 11726329_x_at | 2633 | GBP1 | guanylate binding protein 1, interferon-inducible, 67kDa | NM_002053 | 0,0417226 | 1,53947 | 494 up vs NEG |
| 11719662_s_at | 80013 | FAM188A | family with sequence similarity 188, member A | NM_024948 | 0,0312984 | 1,54004 | 494 up vs NEG |
| 11720653_a_at | 56987 | BBX | bobby sox homolog (Drosophila) | NM_001142568 NM_020235 | 0,00688338 | 1,5426 | 494 up vs NEG |
| 11746031_s_at | 114971 | PTPMT1 | protein tyrosine phosphatase, mitochondrial 1 | NM_001143984 NM_175732 | 0,0203092 | 1,54289 | 494 up vs NEG |
| 11743339_s_at | 79157 | MFSD11 | major facilitator superfamily domain containing 11 | NM_024311 | 0,0192464 | 1,5478 | 494 up vs NEG |
| 11757775_s_at | 387263 | C6orf120 | chromosome 6 open reading frame 120 | NM_001029863 | 0,00532956 | 1,54998 | 494 up vs NEG |
| 11722355_s_at | 1601 | DAB2 | disabled homolog 2, mitogen-responsive phosphoprotein (Drosophila) | NM_001343 | 0,0109181 | 1,55586 | 494 up vs NEG |
| 11716754_a_at | 25941 | C18orf10 | chromosome 18 open reading frame 10 | NM_015476 | 0,0102769 | 1,558 | 494 up vs NEG |
| 11751946_a_at | 57584 | ARHGAP21 | Rho GTPase activating protein 21 | NM_020824 | 0,0322268 | 1,56379 | 494 up vs NEG |
| 11743847_x_at | 3075 | CFH | complement factor H | NM_000186 NM_001014975 | 0,041399 | 1,56596 | 494 up vs NEG |
| 11748507_a_at | 4013 | VWA5A | von Willebrand factor A domain containing 5A | NM_001130142 NM_014622 NM_198315 | 0,0144275 | 1,5875 | 494 up vs NEG |
| 11747841_a_at | 90 | ACVR1 | activin A receptor, type I | NM_001105 NM_001111067 | 0,047846 | 1,59426 | 494 up vs NEG |
| 11727994_at | 8676 | STX11 | syntaxin 11 | NM_003764 | 0,0349612 | 1,59836 | 494 up vs NEG |
| 11743828_s_at | 753 | C18orf1 | chromosome 18 open reading frame 1 | NM_001003674 NM_001003675 NM_004338 NM_181481 NM_181482 NM_181483 | 0,000680081 | 1,60036 | 494 up vs NEG |
| 11722352_s_at | 59 | ACTA2 | actin, alpha 2, smooth muscle, aorta | NM_001141945 NM_001613 | 0,0487308 | 1,60721 | 494 up vs NEG |
| 11758661_s_at | 1031 | CDKN2C | cyclin-dependent kinase inhibitor 2C (p18, inhibits CDK4) | NM_001262 NM_078626 | 0,0355035 | 1,60735 | 494 up vs NEG |
| 11749045_a_at | 202018 | TAPT1 | transmembrane anterior posterior transformation 1 | NM_153365 | 0,0031333 | 1,60804 | 494 up vs NEG |
| 11738047_a_at | 79993 | ELOVL7 | ELOVL family member 7, elongation of long chain fatty acids (yeast) | NM_001104558 NM_024930 | 0,00900182 | 1,61132 | 494 up vs NEG |
| 11733894_x_at | 93624 | TADA2B | transcriptional adaptor 2B | NM_152293 | 0,0298001 | 1,62208 | 494 up vs NEG |
| 11752931_x_at | 2633 | GBP1 | guanylate binding protein 1, interferon-inducible, 67kDa | NM_002053 | 0,0426038 | 1,62768 | 494 up vs NEG |
| 11721294_a_at | 9815 | GIT2 | G protein-coupled receptor kinase interacting ArfGAP 2 | NM_001135213 NM_001135214 NM_014776 NM_057169 NM_057170 NM_139201 | 0,0273856 | 1,63446 | 494 up vs NEG |
| 11755804_a_at | 10507 | SEMA4D | sema domain, immunoglobulin domain (Ig), transmembrane domain (TM) and short cytoplasmi | NM_001142287 NM_006378 | 0,00710385 | 1,64799 | 494 up vs NEG |
| 11736647_s_at | 1398 | CRK | v-crk sarcoma virus CT10 oncogene homolog (avian) | NM_005206 NM_016823 | 0,00994746 | 1,6517 | 494 up vs NEG |
| 11715952_a_at | 8720 | MBTPS1 | membrane-bound transcription factor peptidase, site 1 | NM_003791 | 0,000899147 | 1,67404 | 494 up vs NEG |
| 11743265_a_at | 2029 | ENSA | endosulfine alpha | NM_004436 NM_207042 NM_207043 NM_207044 NM_207045 NM_207046 NM_ | 0,00498515 | 1,67515 | 494 up vs NEG |
| 11735383_s_at | 753 | C18orf1 | chromosome 18 open reading frame 1 | NM_001003674 NM_001003675 NM_004338 NM_181481 NM_181482 NM_181483 | 0,00512772 | 1,70628 | 494 up vs NEG |
| 11747139_a_at | 5921 | RASA1 | RAS p21 protein activator (GTPase activating protein) 1 | NM_002890 NM_022650 | 0,0407711 | 1,70862 | 494 up vs NEG |
| 11729200_at | 387263 | C6orf120 | chromosome 6 open reading frame 120 | NM_001029863 | 0,00888695 | 1,71059 | 494 up vs NEG |
| 11722924_a_at | 22915 | MMRN1 | multimerin 1 | NM_007351 | 0,0272231 | 1,73549 | 494 up vs NEG |
| 11719373_at | 6990 | DYNLT3 | dynein, light chain, Tctex-type 3 | NM_006520 | 0,00663662 | 1,74584 | 494 up vs NEG |
| 11729737_a_at | 91433 | RCCD1 | RCC1 domain containing 1 | NM_001017919 NM_033544 | 0,0188334 | 1,74763 | 494 up vs NEG |
| 11715569_at | 928 | CD9 | CD9 molecule | NM_001769 | 0,0230178 | 1,77549 | 494 up vs NEG |
| 11723215_s_at | 4208 | MEF2C | myocyte enhancer factor 2C | NM_001131005 NM_002397 | 0,00853077 | 1,77551 | 494 up vs NEG |
| 11754319_at | 54434 | SSH1 | slingshot homolog 1 (Drosophila) | NM_001161330 NM_001161331 NM_018984 | 0,0182871 | 1,7996 | 494 up vs NEG |
| 11726062_at | 5739 | PTGIR | prostaglandin I2 (prostacyclin) receptor (IP) | NM_000960 | 0,042897 | 1,80225 | 494 up vs NEG |
| 11721789_a_at | 5789 | PTPRD | protein tyrosine phosphatase, receptor type, D | NM_001040712 NM_001171025 NM_002839 NM_130391 NM_130392 NM_130393 | 0,00640205 | 1,81653 | 494 up vs NEG |
| 11758577_s_at | 79993 | ELOVL7 | ELOVL family member 7, elongation of long chain fatty acids (yeast) | NM_001104558 NM_024930 | 0,0129852 | 1,81889 | 494 up vs NEG |
| 11748235_a_at | 22915 | MMRN1 | multimerin 1 | NM_007351 | 0,0182505 | 1,85083 | 494 up vs NEG |
| 11731446_s_at | 6622 | SNCA | synuclein, alpha (non A4 component of amyloid precursor) | NM_000345 NM_001146054 NM_001146055 NM_007308 | 0,00132609 | 1,8546 | 494 up vs NEG |
| 11754764_a_at | 338382 | RAB7B | RAB7B, member RAS oncogene family | NM_001164522 NM_177403 | 0,00795273 | 1,86223 | 494 up vs NEG |
| 11753731_x_at | 6622 | SNCA | synuclein, alpha (non A4 component of amyloid precursor) | NM_000345 NM_001146054 NM_001146055 NM_007308 | 0,00731478 | 1,8749 | 494 up vs NEG |
| 11731447_x_at | 6622 | SNCA | synuclein, alpha (non A4 component of amyloid precursor) | NM_000345 NM_001146054 NM_001146055 NM_007308 | 0,0079012 | 1,88397 | 494 up vs NEG |
| 11763855_x_at | 928 | CD9 | CD9 molecule | NM_001769 | 0,000723985 | 1,89063 | 494 up vs NEG |
| 11757744_s_at | 221061 | FAM171A1 | family with sequence similarity 171, member A1 | NM_001010924 | 0,0436027 | 1,89597 | 494 up vs NEG |
| 11715570_x_at | 928 | CD9 | CD9 molecule | NM_001769 | 0,0172858 | 1,94956 | 494 up vs NEG |
| 11739782_a_at | 6622 | SNCA | synuclein, alpha (non A4 component of amyloid precursor) | NM_000345 NM_001146054 NM_001146055 NM_007308 | 0,0082021 | 1,95308 | 494 up vs NEG |
| 11722925_x_at | 22915 | MMRN1 | multimerin 1 | NM_007351 | 0,0198681 | 1,96614 | 494 up vs NEG |
| 11761497_a_at | 5532 | PPP3CB | protein phosphatase 3, catalytic subunit, beta isozyme | NM_001142353 NM_001142354 NM_021132 | 0,00890877 | 2,01132 | 494 up vs NEG |
| 11763854_a_at | 928 | CD9 | CD9 molecule | NM_001769 | 0,0231684 | 2,03991 | 494 up vs NEG |
| 11746940_a_at | 928 | CD9 | CD9 molecule | NM_001769 | 0,0238536 | 2,05599 | 494 up vs NEG |
| 11751905_a_at | 4811 | NID1 | nidogen 1 | NM_002508 | 0,0344418 | 2,10388 | 494 up vs NEG |
| 11715568_a_at | 928 | CD9 | CD9 molecule | NM_001769 | 0,00972343 | 2,11424 | 494 up vs NEG |
| 11760342_a_at | 5532 | PPP3CB | protein phosphatase 3, catalytic subunit, beta isozyme | NM_001142353 NM_001142354 NM_021132 | 0,0037151 | 2,12169 | 494 up vs NEG |
| 11735964_at | 94235 | GNG8 | guanine nucleotide binding protein (G protein), gamma 8 | NM_033258 | 0,030287 | 2,15032 | 494 up vs NEG |
| 11720541_at | 29094 | HSPC159 | galectin-related protein | NM_014181 | 0,0016077 | 2,28414 | 494 up vs NEG |
| 11720051_at | 6695 | SPOCK1 | sparc/osteonectin, cwcv and kazal-like domains proteoglycan (testican) 1 | NM_004598 | 0,0319864 | 2,37102 | 494 up vs NEG |

**Table 4 : miR-494-3p downregulated predicted targets.** This is the list of downregulated transcripts upon miR-494-3p overexpression with their context+ score according to TargetScanHuman database.

| **Column ID** | **Entrez Gene** | **Gene Symbol** | **Gene Title** | **RefSeq Transcript ID** | **Fold-Change (494 vs. NEG)** | **TargetScanHuman (context+ score)** | **Site** |
| --- | --- | --- | --- | --- | --- | --- | --- |
| 11758088_s_at | 9306 | SOCS6 | suppressor of cytokine signaling 6 | NM_004232 | -1,65032 | -0,46 | conserved |
| 11719335_a_at | 196513 | DCP1B | DCP1 decapping enzyme homolog B (S. cerevisiae) | NM_152640 | -1,74027 | -0,44 | conserved |
| 11723930_at | 22948 | CCT5 | chaperonin containing TCP1, subunit 5 (epsilon) | NM_012073 | -2,13175 | -0,41 | all PCT |
| 11757793_s_at | 7035 | TFPI | tissue factor pathway inhibitor (lipoprotein-associated coagulation inhibitor) | NM_001032281 NM_006287 | -1,53005 | -0,37 | poorly conserved |
| 11715897_at | 950 | SCARB2 | scavenger receptor class B, member 2 | NM_005506 | -1,58761 | -0,29 | conserved |
| 11735415_a_at | 25800 | SLC39A6 | solute carrier family 39 (zinc transporter), member 6 | NM_001099406 NM_012319 | -2,46496 | -0,29 | poorly conserved |
| 11749546_a_at | 25800 | SLC39A6 | solute carrier family 39 (zinc transporter), member 6 | NM_001099406 NM_012319 | -3,34079 | -0,29 | poorly conserved |
| 11757587_s_at | 25800 | SLC39A6 | solute carrier family 39 (zinc transporter), member 6 | NM_001099406 NM_012319 | -3,2894 | -0,29 | poorly conserved |
| 11725523_at | 1829 | DSG2 | desmoglein 2 | NM_001943 | -1,51238 | -0,28 | conserved |
| 11758440_s_at | 64864 | RFX7 | regulatory factor X, 7 | NM_022841 | -1,73294 | -0,25 | poorly conserved |
| 11721955_at | 64864 | RFX7 | regulatory factor X, 7 | NM_022841 | -2,05998 | -0,25 | poorly conserved |
| 11754110_x_at | 332 | BIRC5 | baculoviral IAP repeat-containing 5 | NM_001012270 NM_001012271 NM_001168 | -1,63066 | -0,24 | all PCT |
| 11754109_s_at | 332 | BIRC5 | baculoviral IAP repeat-containing 5 | NM_001012270 NM_001012271 NM_001168 | -1,83466 | -0,24 | all PCT |
| 11733864_a_at | 332 | BIRC5 | baculoviral IAP repeat-containing 5 | NM_001012270 NM_001012271 NM_001168 | -1,53605 | -0,24 | all PCT |
| 11739603_x_at | 332 | BIRC5 | baculoviral IAP repeat-containing 5 | NM_001012270 NM_001012271 NM_001168 | -1,64526 | -0,24 | all PCT |
| 11747720_a_at | 332 | BIRC5 | baculoviral IAP repeat-containing 5 | NM_001012270 NM_001012271 NM_001168 | -1,56319 | -0,24 | all PCT |
| 11763073_x_at | 332 | BIRC5 | baculoviral IAP repeat-containing 5 | NM_001012270 NM_001012271 NM_001168 | -1,51388 | -0,24 | all PCT |
| 11723558_s_at | 9643 | MORF4L2 | mortality factor 4 like 2 | NM_001142418 NM_001142419 NM_001142420 NM_001142421 NM_001142422 NM | -1,62738 | -0,24 | conserved |
| 11755289_x_at | 9643 | MORF4L2 | mortality factor 4 like 2 | NM_001142418 NM_001142419 NM_001142420 NM_001142421 NM_001142422 NM | -1,56855 | -0,24 | conserved |
| 11733077_a_at | 4659 | PPP1R12A | protein phosphatase 1, regulatory (inhibitor) subunit 12A | NM_001143885 NM_001143886 NM_002480 | -2,21641 | -0,24 | all PCT |
| 11736596_s_at | 84296 | GINS4 | GINS complex subunit 4 (Sld5 homolog) | NM_032336 | -1,70688 | -0,21 | all PCT |
| 11751145_a_at | 23376 | KIAA0776 | KIAA0776 | NM_015323 | -1,56279 | -0,21 | conserved |
| 11758200_x_at | 1163 | CKS1B | CDC28 protein kinase regulatory subunit 1B | NM_001826 NR_024163 | -1,66996 | -0,2 | conserved |
| 11717908_s_at | 84908 | FAM136A | family with sequence similarity 136, member A | NM_032822 | -1,53242 | -0,2 | poorly conserved |
| 11727433_s_at | 10204 | NUTF2 | nuclear transport factor 2 | NM_005796 | -1,77003 | -0,19 | conserved |
| 11758981_a_at | 10204 | NUTF2 | Nuclear transport factor 2 | NM_005796 | -1,65958 | -0,19 | conserved |
| 11753758_x_at | 10204 | NUTF2 | nuclear transport factor 2 | NM_005796 | -1,77609 | -0,19 | conserved |
| 11753757_a_at | 10204 | NUTF2 | nuclear transport factor 2 | NM_005796 | -1,7571 | -0,19 | conserved |
| 11757599_x_at | 10204 | NUTF2 | nuclear transport factor 2 | NM_005796 | -1,79842 | -0,19 | conserved |
| 11727432_a_at | 10204 | NUTF2 | nuclear transport factor 2 | NM_005796 | -1,76206 | -0,19 | conserved |
| 11757938_s_at | 9111 | NMI | N-myc (and STAT) interactor | NM_004688 | -1,73076 | -0,18 | all PCT |
| 11718159_at | 9111 | NMI | N-myc (and STAT) interactor | NM_004688 | -1,83325 | -0,18 | all PCT |
| 11746027_a_at | 9111 | NMI | N-myc (and STAT) interactor | NM_004688 | -1,83151 | -0,18 | all PCT |
| 11718158_a_at | 9111 | NMI | N-myc (and STAT) interactor | NM_004688 | -1,84638 | -0,18 | all PCT |
| 11746028_x_at | 9111 | NMI | N-myc (and STAT) interactor | NM_004688 | -1,67638 | -0,18 | all PCT |
| 11739302_a_at | 8323 | FZD6 | frizzled homolog 6 (Drosophila) | NM_001164615 NM_001164616 NM_003506 | -2,18973 | -0,17 | all PCT |
| 11737879_s_at | 2590 | GALNT2 | UDP-N-acetyl-alpha-D-galactosamine:polypeptide N-acetylgalactosaminyltransferase 2 (Gal | NM_004481 | -2,0845 | -0,17 | conserved |
| 11737878_a_at | 2590 | GALNT2 | UDP-N-acetyl-alpha-D-galactosamine:polypeptide N-acetylgalactosaminyltransferase 2 (Gal | NM_004481 | -1,89676 | -0,17 | conserved |
| 11730760_x_at | 9419 | CRIPT | cysteine-rich PDZ-binding protein | NM_014171 | -1,88025 | -0,16 | all PCT |
| 11730759_s_at | 9419 | CRIPT | cysteine-rich PDZ-binding protein | NM_014171 | -3,56291 | -0,16 | all PCT |
| 11721635_a_at | 79139 | DERL1 | Der1-like domain family, member 1 | NM_001134671 NM_024295 | -1,78082 | -0,16 | all PCT |
| 11721637_s_at | 79139 | DERL1 | Der1-like domain family, member 1 | NM_001134671 NM_024295 | -1,82715 | -0,16 | all PCT |
| 11722410_at | 51809 | GALNT7 | UDP-N-acetyl-alpha-D-galactosamine:polypeptide N-acetylgalactosaminyltransferase 7 (Gal | NM_017423 | -2,01511 | -0,16 | conserved |
| 11722408_at | 51809 | GALNT7 | UDP-N-acetyl-alpha-D-galactosamine:polypeptide N-acetylgalactosaminyltransferase 7 (Gal | NM_017423 | -1,88665 | -0,16 | conserved |
| 11743480_s_at | 9821 | RB1CC1 | RB1-inducible coiled-coil 1 | NM_001083617 NM_014781 | -2,28783 | -0,16 | poorly conserved |
| 11754418_s_at | 10146 | G3BP1 | GTPase activating protein (SH3 domain) binding protein 1 | NM_005754 NM_198395 | -4,40926 | -0,15 | poorly conserved |
| 11722586_a_at | 10146 | G3BP1 | GTPase activating protein (SH3 domain) binding protein 1 | NM_005754 NM_198395 | -3,41808 | -0,15 | poorly conserved |
| 11746111_a_at | 10146 | G3BP1 | GTPase activating protein (SH3 domain) binding protein 1 | NM_005754 NM_198395 | -3,2345 | -0,15 | poorly conserved |
| 11749898_a_at | 9522 | SCAMP1 | secretory carrier membrane protein 1 | NM_004866 | -1,57072 | -0,15 | all PCT |
| 11733055_a_at | 9522 | SCAMP1 | secretory carrier membrane protein 1 | NM_004866 | -2,4322 | -0,15 | all PCT |
| 11733899_a_at | 6738 | TROVE2 | TROVE domain family, member 2 | NM_001042369 NM_001042370 NM_001173524 NM_001173525 NM_004600 NR_03 | -1,50046 | -0,15 | poorly conserved |
| 11744286_s_at | 875 | CBS | cystathionine-beta-synthase | NM_000071 NM_001178008 NM_001178009 | -2,69366 | -0,14 | conserved |
| 11723360_x_at | 5871 | MAP4K2 | mitogen-activated protein kinase kinase kinase kinase 2 | NM_004579 | -1,51635 | -0,14 | all PCT |
| 11733825_s_at | 84886 | C1orf198 | chromosome 1 open reading frame 198 | NM_001136494 NM_001136495 NM_032800 | -2,25617 | -0,13 | all PCT |
| 11758454_s_at | 201627 | FAM116A | family with sequence similarity 116, member A | NM_152678 | -1,75955 | -0,13 | conserved |
| 11740656_a_at | 8091 | HMGA2 | high mobility group AT-hook 2 | NM_003483 NM_003484 | -2,70439 | -0,13 | all PCT |
| 11715305_s_at | 3206 | HOXA10 | homeobox A10 | NM_018951 NM_153715 | -2,27606 | -0,13 | poorly conserved |
| 11763585_s_at | 7112 | TMPO | thymopoietin | NM_001032283 NM_001032284 NM_003276 | -1,50011 | -0,13 | all PCT |
| 11731919_a_at | 7112 | TMPO | thymopoietin | NM_001032283 NM_001032284 NM_003276 | -1,54158 | -0,13 | all PCT |
| 11717841_at | 55500 | ETNK1 | ethanolamine kinase 1 | NM_001039481 NM_018638 | -1,53029 | -0,12 | all PCT |
| 11728405_s_at | 375387 | LRRC33 | leucine rich repeat containing 33 | NM_198565 | -1,51417 | -0,12 | all PCT |
| 11718678_a_at | 5663 | PSEN1 | presenilin 1 | NM_000021 NM_007318 | -1,62259 | -0,12 | all PCT |
| 11734049_a_at | 81929 | SEH1L | SEH1-like (S. cerevisiae) | NM_001013437 NM_031216 | -1,81143 | -0,12 | all PCT |
| 11723781_at | 8540 | AGPS | alkylglycerone phosphate synthase | NM_003659 | -1,79 | -0,11 | conserved |
| 11758491_s_at | 8540 | AGPS | alkylglycerone phosphate synthase | NM_003659 | -2,03707 | -0,11 | conserved |
| 11717626_a_at | 4718 | NDUFC2 | NADH dehydrogenase (ubiquinone) 1, subcomplex unknown, 2, 14.5kDa | NM_004549 | -1,69413 | -0,11 | conserved |
| 11717150_s_at | 79650 | C16orf57 | chromosome 16 open reading frame 57 | NM_024598 | -3,07335 | -0,1 | conserved |
| 11717149_a_at | 79650 | C16orf57 | chromosome 16 open reading frame 57 | NM_024598 | -1,77904 | -0,1 | conserved |
| 11719690_at | 57045 | TWSG1 | twisted gastrulation homolog 1 (Drosophila) | NM_020648 | -2,50419 | -0,1 | poorly conserved |
| 11731624_a_at | 55088 | C10orf118 | chromosome 10 open reading frame 118 | NM_018017 | -1,76931 | -0,09 | all PCT |
| 11726136_a_at | 286527 | TMSB15B | thymosin beta 15B | NM_194324 | -1,92813 | -0,09 | all PCT |
| 11741433_a_at | 372 | ARCN1 | archain 1 | NM_001142281 NM_001655 | -1,87572 | -0,08 | conserved |
| 11742740_at | 25929 | GEMIN5 | gem (nuclear organelle) associated protein 5 | NM_015465 | -1,51787 | -0,08 | all PCT |
| 11717296_s_at | 10128 | LRPPRC | leucine-rich PPR-motif containing | NM_133259 | -2,34072 | -0,08 | all PCT |
| 11717297_at | 10128 | LRPPRC | leucine-rich PPR-motif containing | NM_133259 | -1,89992 | -0,08 | all PCT |
| 11722535_a_at | 151195 | CCNYL1 | cyclin Y-like 1 | NM_001142300 NM_152523 | -1,87976 | -0,07 | poorly conserved |
| 11733261_a_at | 10659 | CELF2 | CUGBP, Elav-like family member 2 | NM_001025076 NM_001025077 NM_001083591 NM_006561 | -1,94387 | -0,07 | all PCT |
| 11721446_a_at | 8723 | SNX4 | sorting nexin 4 | NM_003794 | -1,89598 | -0,07 | all PCT |
| 11723343_at | 23087 | TRIM35 | tripartite motif-containing 35 | NM_171982 | -1,5504 | -0,07 | all PCT |
| 11737747_a_at | 2764 | GMFB | glia maturation factor, beta | NM_004124 | -1,73041 | -0,06 | all PCT |
| 11747498_s_at | 6652 | SORD | sorbitol dehydrogenase | NM_003104 | -1,5384 | -0,06 | all PCT |
| 11720096_a_at | 80774 | LIMD2 | LIM domain containing 2 | NM_030576 | -1,66147 | -0,05 | all PCT |
| 11731506_a_at | 5887 | RAD23B | RAD23 homolog B (S. cerevisiae) | NM_002874 | -1,50651 | -0,05 | conserved |
| 11758199_s_at | 5887 | RAD23B | RAD23 homolog B (S. cerevisiae) | NM_002874 | -1,96462 | -0,05 | conserved |
| 11716620_a_at | 3842 | TNPO1 | transportin 1 | NM_002270 NM_153188 | -1,76296 | -0,05 | poorly conserved |
| 11751051_a_at | 57062 | DDX24 | DEAD (Asp-Glu-Ala-Asp) box polypeptide 24 | NM_020414 | -1,5364 | -0,04 | all PCT |
| 11727426_a_at | 821 | CANX | calnexin | NM_001024649 NM_001746 | -2,18747 | -0,03 | poorly conserved |
| 11751309_a_at | 9093 | DNAJA3 | DnaJ (Hsp40) homolog, subfamily A, member 3 | NM_001135110 NM_005147 | -1,9194 | -0,03 | all PCT |
| 11754598_s_at | 9093 | DNAJA3 | DnaJ (Hsp40) homolog, subfamily A, member 3 | NM_001135110 NM_005147 | -2,3243 | -0,03 | all PCT |
| 11752183_s_at | 9093 | DNAJA3 | DnaJ (Hsp40) homolog, subfamily A, member 3 | NM_001135110 NM_005147 | -1,91644 | -0,03 | all PCT |
| 11715728_a_at | 3688 | ITGB1 | integrin, beta 1 (fibronectin receptor, beta polypeptide, antigen CD29 includes MDF2, M | NM_002211 NM_033666 NM_033667 NM_033668 NM_033669 NM_133376 | -1,879 | -0,03 | all PCT |
| 11715546_s_at | 10951 | CBX1 | chromobox homolog 1 (HP1 beta homolog Drosophila ) | NM_001127228 NM_006807 | -2,24088 | -0,02 | all PCT |
| 11730469_s_at | 4005 | LMO2 | LIM domain only 2 (rhombotin-like 1) | NM_001142315 NM_001142316 NM_005574 | -1,56918 | -0,01 | all PCT |
| 11757512_a_at | 55256 | ADI1 | acireductone dioxygenase 1 | NM_018269 | -1,66286 | / | / |
| 11745196_a_at | 55256 | ADI1 | acireductone dioxygenase 1 | NM_018269 | -1,70583 | / | / |
| 11757564_x_at | 55256 | ADI1 | acireductone dioxygenase 1 | NM_018269 | -1,68862 | / | / |
| 11741556_x_at | 79647 | AKIRIN1 | akirin 1 | NM_001136275 NM_024595 | -1,52545 | / | / |
| 11722721_a_at | 201161 | CENPV | centromere protein V | NM_181716 | -1,55898 | / | / |
| 11718843_s_at | 2790 552891 | DNAJC25-GNG10 GNG10 | DNAJC25-GNG10 readthrough guanine nucleotide binding protein (G protein), gamma 10 | NM_001017998 NM_004125 | -2,27357 | / | / |
| 11759612_at | 64778 | FNDC3B | fibronectin type III domain containing 3B | NM_001135095 NM_022763 | -2,69384 | / | / |
| 11717727_s_at | 51155 | HN1 | hematological and neurological expressed 1 | NM_001002032 NM_001002033 NM_016185 | -2,31609 | / | / |
| 11717726_s_at | 51155 | HN1 | hematological and neurological expressed 1 | NM_001002032 NM_001002033 NM_016185 | -3,13797 | / | / |
| 11721612_a_at | 51194 | IPO11 | importin 11 | NM_001134779 NM_016338 | -1,52198 | / | / |
| 11716468_s_at | 389293 81689 | ISCA1 ISCA1P1 | iron-sulfur cluster assembly 1 homolog (S. cerevisiae) iron-sulfur cluster assembly | NM_001080540 NM_030940 | -3,05682 | / | / |
| 11722213_at | 10360 | NPM3 | nucleophosmin/nucleoplasmin 3 | NM_006993 | -2,52063 | / | / |
| 11721388_a_at | 5026 | P2RX5 | purinergic receptor P2X, ligand-gated ion channel, 5 | NM_002561 NM_175080 NM_175081 | -1,70958 | / | / |
| 11729600_a_at | 5537 | PPP6C | protein phosphatase 6, catalytic subunit | NM_001123355 NM_001123369 NM_002721 | -1,99474 | / | / |
| 11763303_at | 414327 | PS1TP4 | HBV preS1-transactivated protein 4 | --- | -1,78323 | / | / |
| 11758773_at | 201562 | PTPLB | protein tyrosine phosphatase-like (proline instead of catalytic arginine), member b | NM_198402 | -2,94341 | / | / |
| 11752360_s_at | 55696 | RBM22 | RNA binding motif protein 22 | NM_018047 | -1,5987 | / | / |
| 11722398_s_at | 201965 | RWDD4A | RWD domain containing 4A | NM_152682 | -1,50292 | / | / |
| 11722159_a_at | 23428 | SLC7A8 | solute carrier family 7 (amino acid transporter, L-type), member 8 | NM_012244 NM_182728 | -1,6947 | / | / |
| 11764246_s_at | 201595 | STT3B | STT3, subunit of the oligosaccharyltransferase complex, homolog B (S. cerevisiae) | NM_178862 | -1,79998 | / | / |
| 11725277_a_at | 117854 | TRIM6 | tripartite motif-containing 6 | NM_001003818 NM_058166 | -1,69157 | / | / |
| 11715550_at | 1605 | DAG1 | dystroglycan 1 (dystrophin-associated glycoprotein 1) | NM_001165928 NM_001177634 NM_001177635 NM_001177636 NM_001177637 NM | -1,54073 | >-0,01 | all PCT |
| 11730112_a_at | 55635 | DEPDC1 | DEP domain containing 1 | NM_001114120 NM_017779 | -1,53625 | >-0,01 | all PCT |
| 11730111_a_at | 55635 | DEPDC1 | DEP domain containing 1 | NM_001114120 NM_017779 | -2,47303 | >-0,01 | all PCT |
| 11715813_at | 25994 | HIGD1A | HIG1 hypoxia inducible domain family, member 1A | NM_001099668 NM_001099669 NM_014056 | -1,54673 | >-0,01 | all PCT |
| 11753379_a_at | 5594 | MAPK1 | mitogen-activated protein kinase 1 | NM_002745 NM_138957 | -2,13374 | >-0,01 | all PCT |
| 11738872_x_at | 5594 | MAPK1 | mitogen-activated protein kinase 1 | NM_002745 NM_138957 | -2,04718 | >-0,01 | all PCT |
| 11751224_x_at | 5594 | MAPK1 | mitogen-activated protein kinase 1 | NM_002745 NM_138957 | -1,63973 | >-0,01 | all PCT |
| 11738871_a_at | 5594 | MAPK1 | mitogen-activated protein kinase 1 | NM_002745 NM_138957 | -2,07406 | >-0,01 | all PCT |
| 11751223_a_at | 5594 | MAPK1 | mitogen-activated protein kinase 1 | NM_002745 NM_138957 | -1,95926 | >-0,01 | all PCT |
| 11720237_s_at | 5601 | MAPK9 | mitogen-activated protein kinase 9 | NM_001135044 NM_002752 NM_139068 NM_139069 NM_139070 | -1,70722 | >-0,01 | all PCT |
| 11721944_x_at | 11158 | RABL2B | RAB, member of RAS oncogene family-like 2B | NM_001003789 NM_001130919 NM_001130920 NM_001130921 NM_001130922 NM | -1,55792 | >-0,01 | all PCT |
| 11719198_s_at | 894 | CCND2 | cyclin D2 | NM_001759 | -1,97127 | >-0,04 | conserved |
| 11716507_at | 8452 | CUL3 | cullin 3 | NM_003590 | -2,26788 | >-0,05 | conserved |
